# Kinetically matched C–N coupling toward efficient urea electrosynthesis enabled on copper single-atom alloy

**DOI:** 10.1038/s41467-023-42794-2

**Published:** 2023-11-01

**Authors:** Mengqiu Xu, Fangfang Wu, Ye Zhang, Yuanhui Yao, Genping Zhu, Xiaoyu Li, Liang Chen, Gan Jia, Xiaohong Wu, Youju Huang, Peng Gao, Wei Ye

**Affiliations:** 1 College of Material, Chemistry and Chemical Engineering, Key Laboratory of Organosilicon Chemistry and Material Technology, Ministry of Education, Hangzhou Normal University, 311121 Hangzhou, Zhejiang China; 2 College of Materials Science and Engineering, Zhejiang University of Technology, 310014 Hangzhou, Zhejiang China; 3 School of Chemistry and Chemical Engineering, Harbin Institute of Technology, 150001 Harbin, Heilongjiang P. R. China

**Keywords:** Materials for energy and catalysis, Electrocatalysis

## Abstract

Chemical C–N coupling from CO_2_ and NO_3_^–^, driven by renewable electricity, toward urea synthesis is an appealing alternative for Bosch–Meiser urea production. However, the unmatched kinetics in CO_2_ and NO_3_^–^ reduction reactions and the complexity of C- and N-species involved in the co-reduction render the challenge of C–N coupling, leading to the low urea yield rate and Faradaic efficiency. Here, we report a single-atom copper-alloyed Pd catalyst (Pd_4_Cu_1_) that can achieve highly efficient C–N coupling toward urea electrosynthesis. The reduction kinetics of CO_2_ and NO_3_^–^ is regulated and matched by steering Cu doping level and Pd_4_Cu_1_/FeNi(OH)_2_ interface. Charge-polarized Pd^δ–^-Cu^δ+^ dual-sites stabilize the key *CO and *NH_2_ intermediates to promote C–N coupling. The synthesized Pd_4_Cu_1_-FeNi(OH)_2_ composite catalyst achieves a urea yield rate of 436.9 mmol g_cat._^–1^ h^–1^ and Faradaic efficiency of 66.4%, as well as a long cycling stability of 1000 h. In-situ spectroscopic results and theoretical calculation reveal that atomically dispersed Cu in Pd lattice promotes the deep reduction of NO_3_^–^ to *NH_2_, and the Pd-Cu dual-sites lower the energy barrier of the pivotal C–N coupling between *NH_2_ and *CO.

## Abstract (web summary)

The synthesis of urea by electrochemically converting the waste of nitrate and carbon dioxide is an interesting approach, but currently still restricted to low urea yield rate and Faradaic efficiency. Here, the authors report an efficient copper single-atom alloy electrocatalyst toward urea electrosynthesis.

## Introduction

Urea (CO(NH_2_)_2_) is a vital chemical fertilizer in modern society, which greatly promotes the development of agriculture and contributes to the rapid growth of world’s population^1–3^. Industrial urea production relies on the Bosch–Meiser process, in which carbon dioxide (CO_2_) and ammonia (NH_3_) are thermochemically coupled operated at elevated temperatures (~200 °C) and high pressures (~210 bar)^4^. Approximately 80% of industrial NH_3_ produced by the Haber–Bosch process is fed for the urea production^5^. Consequently, the harsh conditions in urea synthesis consume substantial fossil fuels, and which leads to serious CO_2_ release. Urea electrosynthesis from CO_2_ and nitrogenous compounds is an attractive alternative approach by taking advantage of the in situ generated C- and N-intermediates. As the electrolytic reactions can be carried out at room temperature and atmospheric pressure, the energy efficiency can be greatly improved. Nonetheless, restricted by the inert N ≡ N bond (bond energy of 941 kJ mol^–1^) and low solubility of N_2_ in aqueous electrolytes, the urea electrosynthesis from CO_2_ and N_2_ delivers low urea yield rates (typically <5 mmol g_cat._^–1^ h^–1^) and urea Faradaic efficiency (FE, <20%)^6–8^. The nitrate ions (NO_3_^–^) reduction reaction (NO_3_RR) is easier than N_2_ reduction, due to the lower N = O bond energy (206 kJ mol^–1^) and much higher solubility of NO_3_^–^ ^9,10^. Nitrate ions are also an abundant feedstock, mainly come from industrial wastewater, chemical fertilizers, and livestock excrement, which may serve as ideal candidates for the C–N coupling^11^.

Urea yield rate and urea FE in urea electrosynthesis from CO_2_ and NO_3_^–^ are still insufficient compared to the thresholds of economic viability predicted by techno-economic assessments. An efficient C–N coupling electrocatalyst should possess the following features. First, the matched kinetics of NO_3_RR and CO_2_ reduction reaction (CO_2_RR) is the prerequisite to boost urea yield rate and FE (see Supplementary Fig. 1). Second, the adjacent dual-sites are required to stabilize C- and N-intermediates, respectively and lower the energy barrier of C–N coupling. Third, the possible by-products should be effectively restrained to ensure high urea FE as varieties of C- and N-species are inevitably involved in the co-reduction process (e.g., CO, CH_4_, CH_3_OH and HCOOH in CO_2_RR, NO_2_^–^, NH_3_, NH_2_OH, N_2_ in NO_3_RR)^12–16^. Taken these regards, electrocatalyst with tunable dual-sites is an ideal choice to induce the formation and stabilize the pivotal C- and N-intermediates (*CO and *NH_2_, * denotes the active site) for C–N coupling^15,17^. As *CO is electron deficient and *NH_2_ is electron efficient, constructing M_1_^δ–^-M_2_^δ+^ (e.g., M_1_ = Pd, M_2_ = Cu) type dual-sites with charge polarization seems to be effective for stabilization of the key intermediates.

Here, we design the charge-polarized Pd^δ–^-Cu^δ+^ dual-sites in copper single-atom alloy toward efficient electrochemical C–N coupling. Atomically dispersed Cu atoms in Pd lattice accelerate NO_3_RR by promoting the deep reduction of NO_2_^–^ to *NH_2_. Meanwhile, the reduction of CO_2_ to CO is also strengthened, while the desorption process of *CO is restrained on Cu single-atom alloy. Therefore, the kinetics of NO_3_RR and CO_2_RR is well matched with N- and C-intermediates yield rate ratio of 1.5, which is close to the stoichiometric ratio (2:1) in urea. In situ Raman spectroscopic characterizations combined with theoretical calculation reveal that Pd^δ–^-Cu^δ+^ dual-sites stabilize the two key intermediates (*CO and *NH_2_) for C–N coupling, respectively. Benefitting from the matched kinetics and charge-polarized dual-sites in Cu single-atom alloy, Pd_4_Cu_1_-Ni(OH)_2_ catalyst delivers urea yield rate of 60.4 mmol g_cat._^–1^ h^–1^ and urea FE of 64.4% in gas diffusion electrode (GDE, catalyst loading: 0.1 mg cm^–2^). Further optimizing the carrier with Fe-doping in Ni(OH)_2_ to accelerate water dissociation and improve the yield rates of N- and C-intermediates, the Pd_4_Cu_1_-FeNi(OH)_2_ composite catalyst delivers the urea yield rate of 436.9 mmol g_cat._^–1^ h^–1^ and FE of 66.4%, together with the high catalytic stability up to 1000 h in GDE.

## Results

### Synthesis and structural characterization of electrocatalysts

**Fig. 1:**
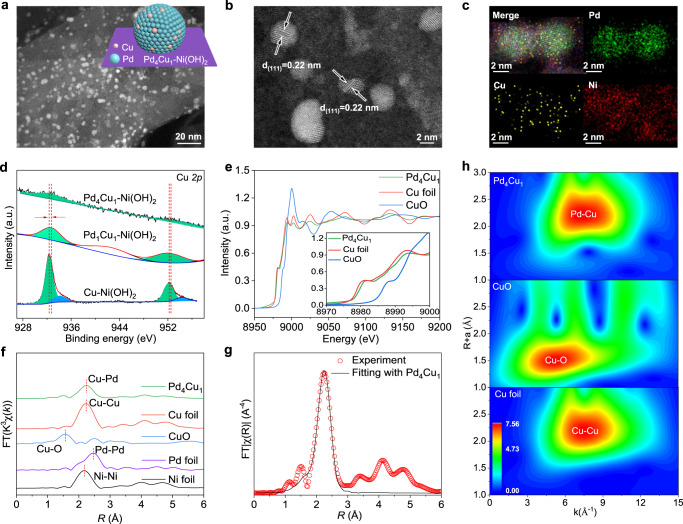
Characterization of Pd_4_Cu_1_-Ni(OH)_2_ sample. **a** HAADF-STEM image, **b** high-resolution HAADF-STEM image, **c** EDS elemental mapping profile of Pd_4_Cu_1_-Ni(OH)_2_ composite structure. **d** Cu *2p* spectra of Pd_4_Cu_1_-Ni(OH)_2_, Pd_1_Cu_1_-Ni(OH)_2_ and Cu-Ni(OH)_2_. **e** Normalized Cu K-edge XANES spectra of Pd_4_Cu_1_ clusters in reference with Cu foil and CuO, **f** *k*^3^-weighted Fourier-transform Cu K-edge, Pd K-edge and Ni K-edge EXAFS spectra, **g** the experimental Cu K-edge EXAFS spectrum (red circle) and the fitting curve (black line) of Pd_4_Cu_1_. **h** Wavelet transforms of the *k*^*2*^*-*weighted Cu K-edge EXAFS signals for the high-coordination shells in reference with Cu foil and CuO. The inset in **a** shows schematic diagram of Pd_4_Cu_1_-Ni(OH)_2_.

Atomic dispersion of Cu in Pd lattice was synthesized by co-reduction of PdCl_4_^2–^ and Cu^2+^ with NaBH_4_ as a reducing agent. Ultrathin layered α-Ni(OH)_2_ nanosheets were employed to accelerate water splitting to produce more active hydrogen atoms and used as catalyst carrier (Supplementary Fig. 2). The synthetic process of the composite electrocatalyst is demonstrated in Supplementary Fig. 3. Cu doping level in Pd host was controlled by regulating the molar ratios of Pd:Cu precursors. As shown in Supplementary Table 1, the molar ratios of Pd:Cu in the as-synthesized products determined by inductively coupled plasma-mass spectrometry (ICP-MS) are consistent with these of Pd:Cu precursors. Therefore, the samples are denoted as Pd_*x*_Cu_1_-Ni(OH)_2_ (*x* = 1, 2, 3, 4, 5, 6). Among which, solid solution phase alloy, i.e., Pd_1_Cu_1_ clusters, are formed. Atomic dispersion of Cu atoms in Pd lattice is formed by decreasing Cu doping level to Pd:Cu ratio of 4:1^18^. Powder X-ray diffraction (XRD) patterns of the composite samples only display the diffraction patterns of α-Ni(OH)_2_, without face-centered cubic (fcc) phase Pd/Cu (Supplementary Fig. 4). Transmission electron microscopic (TEM, Supplementary Fig. 5) characterization demonstrates that the metal clusters are anchored on Ni(OH)_2_ nanosheets. Taken Pd_4_Cu_1_-Ni(OH)_2_ as an example, aberration-corrected high-angle annular dark-field scanning TEM (HAADF-STEM, Fig. 1a and TEM image in Supplementary Fig. 6) image shows that Pd_4_Cu_1_ clusters with average size of 3.5 ± 0.1 nm are uniformly distributed on α-Ni(OH)_2_ nanosheets. High-resolution HAADF-STEM (Fig. 1b) image indicates the spherical Pd_4_Cu_1_ nanoparticles, where the lattice distance of 0.22 nm can be attributed to (111) plane of fcc Pd/Cu.

The elemental mapping profile (Fig. 1c) indicates a uniform distribution of Pd and Cu across Pd_4_Cu_1_ cluster, manifesting a uniform Cu doping in Pd lattice^19^. Then, X-ray photoelectron spectroscopic (XPS, Supplementary Fig. 7) result confirms the existence of Pd and Cu with molar ratio approaching 4:1, consistent with ICP-MS result. As shown in Fig. 1d, the binding energy of Cu *2p*_3/2_ for metallic Cu shifts from 932.3 eV to higher value of 932.6 eV for Pd_1_Cu_1_ and Pd_4_Cu_1_ clusters. The result indicates that electrons are denoted from Cu to adjacent Pd atoms, due to the larger electronegativity of Pd atoms than Cu, leading to the formation of charge-polarized Pd^δ–^-Cu^δ+^ dual-sites^20,21^. In addition, a satellite peak around 941.4 eV can be assigned to Cu^2+^ in Pd_1_Cu_1_-Ni(OH)_2_ sample^22^.

To decode the exact fine structure of copper single-atom alloy structure, Pd_4_Cu_1_-Ni(OH)_2_ was characterized by synchrotron radiation-based X-ray absorption fine structure (XAFS) spectroscopy. Figure 1e shows Cu K-edge X-ray absorption near edge structure (XANES) spectra of Pd_4_Cu_1_-Ni(OH)_2_ in reference with CuO and Cu foil. The intensity (the insert in Fig. 1e) of Cu K-edge between 8975 and 8995 eV for Pd_4_Cu_1_-Ni(OH)_2_ sample is slightly lower than that of Cu foil. It manifests that the valence of Cu^δ+^ in Pd_4_Cu_1_ is approaching Cu^0^ but slightly higher than Cu^0^, confirming the charge polarization (Cu^δ+^ → Pd^δ–^) between Cu and adjacent Pd atoms^23^. Cu extended XAFS (EXAFS) spectra were obtained through a Fourier transformation of Cu K-edge spectra (Fig. 1f). The fine crystalline structure is confirmed by fitting the *k*^3^-weighted Fourier transformed EXAFS spectra (Fig. 1g and Supplementary Fig. 8). In contrast with Cu foil, Cu–Cu bond is absent in Pd_4_Cu_1_-Ni(OH)_2_ sample. Cu–Pd bond (2.61 Å) is resolved in the first shell with a coordination number (CN, Supplementary Table 2) of 10.7, verifying the isolated Cu atoms in Pd lattice^24,25^. Besides, Cu–O bond (2.05 Å, CN = 3.1) is also observed in Pd_4_Cu_1_-Ni(OH)_2_ sample, revealing partial oxidation of Cu atoms^18^. Then, wavelet transforms (WT) analysis of the Cu K-edge EXAFS oscillations of Pd_4_Cu_1_-Ni(OH)_2_ sample was performed in reference with CuO, Cu foil. Two dimensional contour maps of Pd_4_Cu_1_-Ni(OH)_2_ in Fig. 1h resolve Pd–Cu bond, while Cu–Cu bond is absent determined by the wave vector number (**k**). The fine structure of Pd was also resolved by XAFS (Supplementary Fig. 9). Putting together the above results, we come to a conclusion that Cu is atomically dispersed in Pd lattice, namely Cu single-atom alloy.

### Evaluation of catalytic performance

**Fig. 2:**
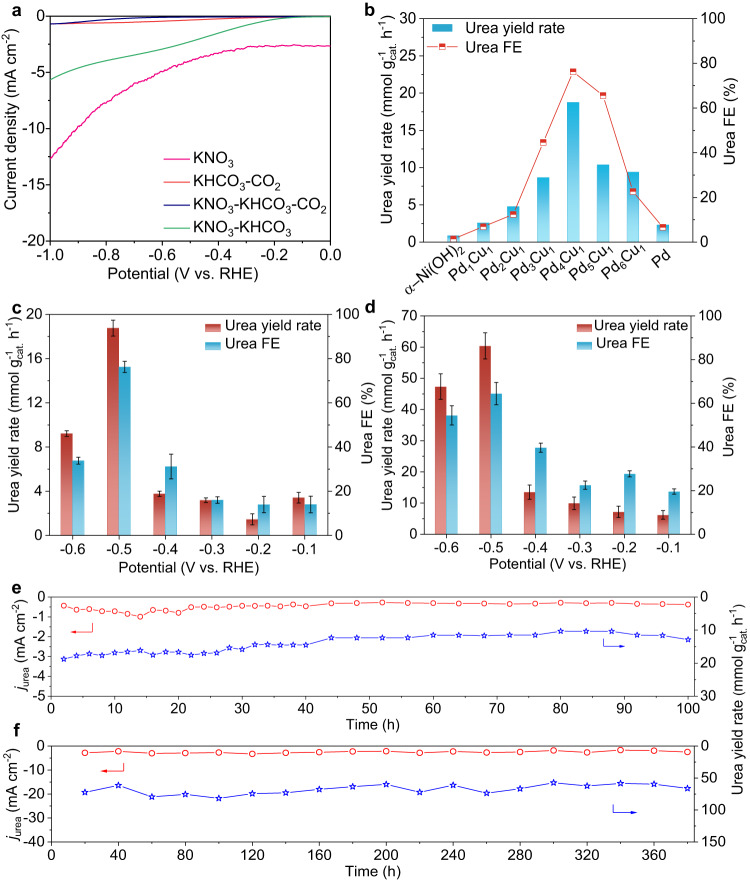
Urea electrosynthesis performance. **a** LSV curves of Pd_4_Cu_1_-Ni(OH)_2_ recorded in the mixture of 0.1 M KHCO_3_ + 0.1 M KNO_3_ (pH=8.4) under CO_2_ flow in reference with that in 0.1 M KNO_3_, 0.1 M KHCO_3_ + CO_2_, 0.1 M KNO_3_ + 0.1 M KHCO_3_. **b** Screening electrocatalysts toward urea electrosynthesis with Pd_x_Cu_1_-Ni(OH)_2_ composite samples. Potential-dependent urea yield rates and FEs of Pd_4_Cu_1_-Ni(OH)_2_ in **c** H-type cell and **d** GDE with catalyst loading: 0.1 mg cm^–2^. Cycling stability of Pd_4_Cu_1_-Ni(OH)_2_ catalyst in urea electrosynthesis assessed **e** in H-type cell and **f** in GDE. **c**, **d** Error bars in accordance with the standard deviation of at least three independent measurements.

Urea electrosynthesis test was carried out in an H-type cell at room temperature with gaseous CO_2_ and KNO_3_ as C- and N-sources, respectively. Linear sweep voltammetry (LSV) test was initially carried out to evaluate current response for Pd_4_Cu_1_-Ni(OH)_2_ sample. As shown in Fig. 2a, the current densities are in the sequence of *I*(KNO_3_) > *I*(KNO_3_ + KHCO_3_) > *I*(KHCO_3_ + CO_2_) > *I*(KNO_3_ + KHCO_3_ + CO_2_). The results indicate that the co-reduction of NO_3_^–^ and CO_2_ toward C–N coupling delivers lower current density than that of solo NO_3_RR or CO_2_RR, suggesting NO_3_RR, CO_2_RR and the competing hydrogen evolution reaction are effectively suppressed in the co-electrolysis^3,12^. Then, we screened the optimal urea yield rate and FE at –0.5 V versus reversible hydrogen electrode (RHE) over Pd_x_Cu_1_-Ni(OH)_2_ composite catalysts in H-type cell, in contrast with bare Ni(OH)_2_ nanosheets or Pd-Ni(OH)_2_ sample. The loading amount of Pd_x_Cu_1_ in the sample toward urea electrosynthesis was firstly optimized (Supplementary Fig. 10). The produced amount of urea in the electrolyte was spectrophotometrically quantified using diacetyl monoxime as chromogenic reagent (Supplementary Fig. 11)^3^. As shown in Fig. 2b, urea yield rates and urea FEs all show a volcano-shape variation trend with Pd:Cu molar ratios (Pd_*x*_Cu_1_, *x* = 1–6). Notably, Pd_*x*_Cu_1_-Ni(OH)_2_ (x = 1–6) composite electrocatalysts all deliver higher urea electrosynthesis performance than that of bare Ni(OH)_2_ nanosheets (0.9 mmol g_cat._^–1^ h^–1^, 1.4%) and Pd-Ni(OH)_2_ (2.3 mmol g_cat._^–1^ h^–1^, 6.6%). The optimal urea yield rate and urea FE are 18.8 mmol g_cat._^–1^ h^–1^ and 76.2% achieved on Pd_4_Cu_1_-Ni(OH)_2_ sample with urea partial current density of 0.68 mA cm^–2^ (Supplementary Fig. 14a). Urea yield rates are about 20.9- and 8.2-fold higher than that of bare Ni(OH)_2_ and Pd-Ni(OH)_2_ counterparts, respectively. The above results indicate that alloying Cu single-atoms in Pd lattice really boosts urea electrosynthesis performance (Supplementary Fig. 12).

Then, potential-dependent urea yield rates and FEs of Pd_4_Cu_1_-Ni(OH)_2_ in H-type cell were also assessed (Supplementary Fig. 13). As indicated in Fig. 2c, urea yield rates are 3.4, 1.5, 3.2, 3.8, 18.8 and 9.2 mmol g_cat._^–1^ h^–1^ at –0.1, –0.2, –0.3, –0.4, –0.5 and –0.6 V, respectively. Correspondingly, urea FEs are 14.0%, 14.0%, 16.0%, 31.1%, 76.2% and 33.8%. To exclude the impact of NO_2_^–^ in the electrolyte derived from NO_3_RR on urea determination, the produced amount of urea in the electrolyte was also quantified through spectrophotometric method with urease and ^1^H-NMR spectroscopy (Supplementary Figs. 15–17)^26^. In addition, N- and C-selectivity reaches 88.6% and 96.1% (Supplementary Fig. 18) in urea electrosynthesis at –0.5 V, respectively. ^15^N isotope labeling experiments (^15^NO_3_^–^ as feeding) were carried out to further confirm the produced urea was rooted from the C–N coupling of NO_3_^–^ and CO_2_ (Supplementary Figs. 19 and 20)^9^. To show the unique promotion role of Cu single-atom alloy, we also screened the transition metals in single-atom alloys (Pd_4_X_1_, X=Fe, Co, Ni, Cu, Zn) for C–N coupling, and the result indicates the best choice of Cu (Supplementary Figs. 21 and 22).

Urea electrosynthesis was further assessed in commercial GDE (Supplementary Fig. 23) to improve mass transfer of CO_2_. Figure 2d shows potential-dependent urea yield rates and FEs of Pd_4_Cu_1_-Ni(OH)_2_ in GDE with CO_2_ flow rate of 20 mL min^–1^. Urea yield rates are 6.2, 7.2, 9.9, 13.5, 60.4 and 47.3 mmol g_cat._^–1^ h^–1^ at –0.1, –0.2, –0.3, –0.4, –0.5 and –0.6 V, respectively, which are obviously higher than that in H-type cell. Urea FEs are 19.6%, 27.7%, 22.5%, 39.6%, 64.4% and 54.5% between –0.1 and –0.6 V. Urea partial current density in GDE increases to 2.3 mA cm^–2^ at –0.5 V (Supplementary Fig. 14b, c). The optimal urea yield rate (60.4 mmol g_cat._^–1^ h^–1^) and FE (64.4%) at –0.5 V exceed the current state-of-the-art electrocatalysts as summarized in Supplementary Table 3.

Apart from urea yield rate and FE, cycling stability is another important parameter in the catalyst evaluation. As shown in Fig. 2e, urea partial current density (*j*_urea_) in H-type cell stabilizes in the initial 40 h, and then slightly declines in the following 60 h. In addition, urea yield rate slightly declines to 12.9 mmol g_cat._^–1^ h^–1^ at 100 h with retention of 68.7%. After durability test (100 h), Pd_4_Cu_1_ still sustains cluster structure on Ni(OH)_2_ nanosheets without obvious size changes, confirming the rigidity of our catalyst (Supplementary Fig. 24). We also assessed cycling stability in GDE (Fig. 2f). Amazingly, Pd_4_Cu_1_-Ni(OH)_2_ composite catalyst can stably sustain continuous 380 h test without obvious urea partial current density and urea yield rate decay. The service life of Pd_4_Cu_1_-Ni(OH)_2_ catalyst is an order of magnitude higher than that of the reported catalysts (Supplementary Table 3, typically ≤30 h).

### Mechanistic study

**Fig. 3:**
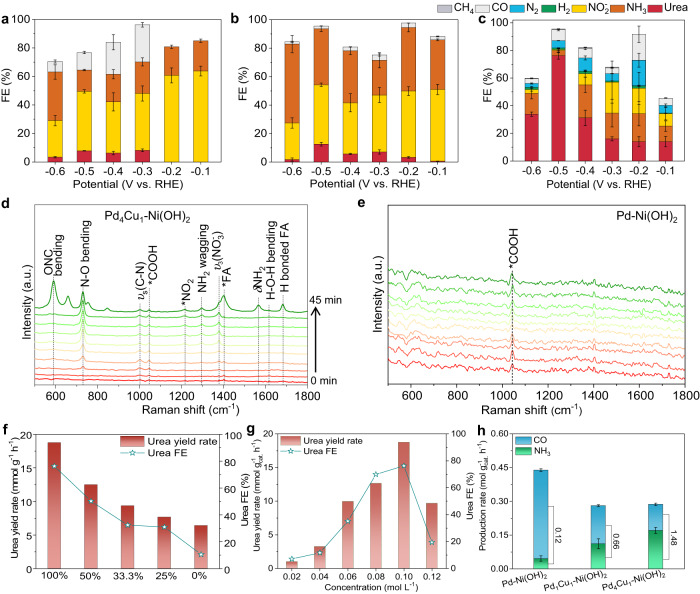
Mechanistic study. FEs of the primary products in urea electrosynthesis for **a** Pd-Ni(OH)_2_, **b** Pd_1_Cu_1_-Ni(OH)_2_, and **c** Pd_4_Cu_1_-Ni(OH)_2_ composite catalysts assessed in 0.1 M KHCO_3_ + 0.1 M KNO_3_ (catalyst loading: 0.1 mg cm^–2^). Time-resolved in situ Raman spectra recorded in urea electrosynthesis at –0.5 V from 0 to 45 min: **d** Pd_4_Cu_1_-Ni(OH)_2_, **e** Pd-Ni(OH)_2_. Urea yield rates and urea FEs **f** at different CO_2_ partial pressure and **g** different concentrations of NO_3_^–^ for Pd_4_Cu_1_-Ni(OH)_2_ at –0.5 V. **h** Production rates of CO and NH_3_ in solo CO_2_RR and NO_3_RR, and the corresponding ratios of NH_3_:CO at –0.5 V. **a**–**c**, **h** Error bars in accordance with the standard deviation of at least three independent measurements.

Upon assessing the performance of urea electrosynthesis, it is essential to decode the unique role of copper single-atom alloy in C–N coupling. Considering the variety of by-products involved in NO_3_RR and CO_2_RR processes, FE is an important indicator to examine the influence of atomically dispersed Cu atoms in Pd host in urea electrosynthesis (Supplementary Figs. 25–28). Electrochemical performance of Pd_4_Cu_1_-Ni(OH)_2_ sample in solo NO_3_RR or CO_2_RR was firstly assessed, NH_3_ and CO were the main products (Supplementary Fig. 29), respectively. Notably, NH_3_ and CO yield rates are much higher than urea yield rates, suggesting C–N coupling toward urea synthesis possesses sluggish kinetics, consistent with LSV curves (Fig. 2a). The results also indicate that the co-reduction of NO_3_^–^ and CO_2_ inhibits the single NO_3_RR or CO_2_RR. Figure 3a–c show the FEs of the primary products for Pd-Ni(OH)_2_, Pd_1_Cu_1_-Ni(OH)_2_, Pd_4_Cu_1_-Ni(OH)_2_ composite catalysts, respectively. NO_2_^–^ FEs are dominated between –0.1 and –0.6 V for Pd-Ni(OH)_2_ sample, suggesting that metallic Pd catalyst enclosed by (111) plane can catalyze the conversion of NO_3_^–^ to NO_2_^–^, and the deep reduction of NO_2_^–^ to NH_3_ process is interrupted (Fig. 3a)^27^. Notably, CO and urea synchronously emerge at –0.3 V, that is because CO_2_RR is triggered at more negative potential^28,29^. The result also indicates that the production of CO is a prerequisite for C–N coupling toward urea formation^14,17^. As shown in Fig. 3b, the formation of CO and urea is synchronously advanced to –0.2 V on Pd_1_Cu_1_-Ni(OH)_2_ sample, further supporting the conclusion. In addition, NH_3_ FEs all increase compared with that of Pd-Ni(OH)_2_ between –0.1 and –0.6 V. That is because Cu is active for NO_3_RR to NH_3_, and alloying Cu atoms in Pd lattice facilitates the deep reduction of NO_2_^–^ to NH_3_^25^. Accordingly, urea FE increases from 7.9% of Pd-Ni(OH)_2_ to 12.6% of Pd_1_Cu_1_-Ni(OH)_2_ at –0.5 V, verifying that the enhanced NO_3_RR facilitates urea synthesis. It is reasonable to infer that the key N-intermediate for C–N coupling comes from the conversion process of NO_2_^–^ to NH_3_, not NO_2_^–^. As Cu doping level in Pd lattice declines to Pd_4_Cu_1_, namely Cu single-atom alloy, urea FEs all greatly increase and the FEs of by-products (e.g., NO_2_^–^, NH_3_, CO) decrease between –0.1 and –0.6 V (Fig. 3c). The optimal urea FE reaches 76.2% at –0.5 V, while NH_3_ FE decreases to 3.7%. Moreover, a very small percentage of methane arises between –0.1 and –0.3 V for Pd_4_Cu_1_-Ni(OH)_2_. From the above results, we can conclude that NO_3_RR is greatly enhanced, and then C–N coupling toward urea formation is boosted.

To figure out the possible C- and N-intermediates for C–N coupling, a list of control experiments were carried out. As shown in Table 1, the possible C-intermediates, e.g., HCOOH, CH_3_OH, HCHO and CO were employed as C-feeding, while NO_3_^–^ was employed as N-feeding. From entry 1-5, urea is obtained using HCOOH and CO as C-feeding. It is generally accepted that CO is the downstream reduction product of CO_2_RR (CO_2_ to *COOH to *CO)^30^. Therefore, we can conclude that *CO is the C-intermediate for C–N coupling toward urea synthesis, consistent with FEs result. Meanwhile, a series of N-intermediates, e.g., NO_2_^–^, NH_2_OH, HCONH_2_ (formamide, FA), NH_3_, NH_4_^+^, were employed to replace NO_3_^–^. From entry 6–10, urea is only detected in the electrolytes with NO_2_^–^, NH_2_OH or HCONH_2_. Obviously, urea is not formed by C–N coupling with NH_3_ or NH_4_^+^ as N-intermediates. From entry 8, we infer that *CONH_2_ may be the possible intermediate in urea synthesis, which is considered to be formed by a nucleophilic attack coupling of *CO and *NH_2_^31^. As such, *NH_2_ and *CO are N-intermediates and C-intermediates for C–N coupling toward urea formation.

**Table 1:** The list of control experiments carried out to elucidate the mechanistic pathway towards urea at –0.5 V for 2 h

Entry	C-source	N-source	Urea?	Electrolyte solution
1	CO_2_	KNO_3_	√	100 mM KNO_3_
2	HCOOH	KNO_3_	√	100 mM KNO_3_ + 20 mM HCOOH
3	HCHO	KNO_3_	×	100 mM KNO_3_ + 20 mM HCHO
4	CH_3_OH	KNO_3_	×	100 mM KNO_3_ + 20 mM CH_3_OH
5	CO	KNO_3_	√	20 mM KNO_3_
6	KHCO_3_ + CO_2_	KNO_2_	√	20 mM KNO_2_ + 100 mM KHCO_3_
7	KHCO_3_ + CO_2_	NH_2_OH	√	20 mM NH_2_OH + 100 mM KHCO_3_
8	KHCO_3_ + CO_2_	HCONH_2_	√	20 mM HCONH_2_ + 100 mM KHCO_3_
9	KHCO_3_ + CO_2_	NH_3_	×	20 mM NH_3_ + 100 mM KHCO_3_
10	KHCO_3_ + CO_2_	NH_4_Cl	×	20 mM NH_4_Cl + 100 mM KHCO_3_

To reveal C–N coupling mechanism on Pd_4_Cu_1_-Ni(OH)_2_ sample, in situ Raman spectroscopic characterization was performed to trace the evolution of C- and N-species. Figure 3d, e and Supplementary Fig. 30 show the time-resolved Raman spectra in urea electrosynthesis at –0.5 V, recorded on Pd_4_Cu_1_-Ni(OH)_2_, Pd-Ni(OH)_2_ and Pd_1_Cu_1_-Ni(OH)_2_, respectively. As shown in Fig. 3d, vibrational peaks located at 730 and 1378 cm^–1^ can be attributed to a N–O bending mode and *ν*_3_ mode of free NO_3_^–^, respectively^32,33^. The intensity of the two peaks gradually increases with reaction time, suggesting the enrichment of NO_3_^–^ on catalyst surface^34^. Two vibrational peaks located at 1216 and 1296 cm^–1^ synchronously appear at 20 min, which are assigned to *NO_2_ and *NH_2_ wagging modes, respectively^35,36^. It suggests that NO_3_^–^ is reduced to *NO_2_, and then to *NH_2_. A vibrational peak located at 1000 cm^–1^ ascribing to *ν*_s_(C–N) mode of urea arises at 10 min, validating the formation of urea^37^. When the reaction proceeded to 45 min, vibrational peaks located at 590, 1402, 1567, 1683 cm^–1^ appeared with high intensity, which can be attributed to OCN bending mode, C–H in-plane bending mode, *δ*NH_2_ of formamide (FA) and H bonded FA signal (Supplementary Table 4), respectively^38^. The emergence of FA signal indicates that FA is really the intermediate product of C–N coupling toward urea formation. Notably, FA usually exhibits stronger Raman signal intensity than urea, which well explains the sudden emergence of a strong FA signal on Pd_4_Cu_1_-Ni(OH)_2_ (Supplementary Fig. 31). Beyond that, a vibrational peak located at 1046 cm^–1^ appears at 10 min, which is assigned to *COOH rooted from CO_2_RR^39^.

For Pd_1_Cu_1_-Ni(OH)_2_ sample, the vibrational signals of *NO_2_ and *NH_2_ arise at 45 min with lower intensity, suggesting that the conversion of NO_3_^–^ to *NO_2_ and *NO_2_ to *NH_2_ possess sluggish kinetics on Pd_1_Cu_1_ alloy (Supplementary Fig. 30). *ν*_s_(C–N) vibrational peak of urea can hardly be observed, suggesting that trace of urea is formed on Pd_1_Cu_1_ clusters. The characteristic vibrational peaks of FA, i.e., OCN bending mode, C–H in-plane bending mode, *δ*NH_2_ and H bonded FA, are also observed. The result indicates that the formation of urea on Pd_1_Cu_1_ alloy undergoes the similar pathway with Cu single-atom alloy. Furthermore, the signal of *COOH appears in the initial 5 min, indicating that CO_2_ reduction to *COOH is not affected on Pd_1_Cu_1_ alloy. As such, the sluggish reduction kinetics of NO_3_^–^ to *NH_2_ is the possible reason for the low urea yield on Pd_1_Cu_1_ alloy. As a stark contrast, only *COOH is observed for Pd-Ni(OH)_2_, no *NO_2_ and *NH_2_ signal appear, suggesting NO_3_RR is inhibited on metallic Pd (Fig. 3e), further verifying single-atom Cu in Pd lattice facilitates NO_3_RR and then urea synthesis.

We further examined the evolution of Raman signal of *CO, which is the key C-intermediates for C–N coupling. As shown in Supplementary Fig. 32, the bridged *CO located at 2080 cm^–1^ on Pd_4_Cu_1_-Ni(OH)_2_ sample exhibits weaker Raman vibrational signal than Pd_1_Cu_1_-Ni(OH)_2_ and Pd-Ni(OH)_2_^40^. That is because the produced *CO is quickly consumed by *NH_2_ for C–N coupling. For metallic Pd catalyst, two vibrational peaks located at 2050 and 2135 cm^–1^ arose at 35 and 40 min, which were assigned to bridge type and linear type *CO, respectively^41^. From the above Raman spectroscopic results, we can conclude that *NO_2_ to NH_3_ in NO_3_RR is inhibited on metallic Pd surface, which could not provide sufficient *NH_2_ species for further C–N coupling. As such, CO and NO_2_^–^ are the primary products in the co-reduction of CO_2_ and NO_3_^–^, well explaining high CO and low urea FEs on Pd-Ni(OH)_2_ sample. When Cu is doped in Pd lattice to form Pd_1_Cu_1_ alloy, NO_3_RR conversion is promoted and urea yield rate increases accordingly. As the Cu doping level is reduced to atomic dispersion, *NO_2_ to NH_3_ and C–N coupling processes are all accelerated, and urea yield rate and FE are boosted.

From the above results, we can infer the kinetics of CO_2_RR and NO_3_RR determines the final urea electrosynthesis. To confirm the conclusion, we further regulated the kinetics of CO_2_RR and NO_3_RR by changing CO_2_ partial pressure or the concentration of NO_3_^–^ to slow down CO_2_RR and NO_3_RR kinetics. As shown in Fig. 3f, g, urea yield rates and urea FEs all show decreasing trend with the CO_2_ partial or NO_3_^–^ concentrations, suggesting that the kinetics of CO_2_RR and NO_3_RR indeed determines urea electrosynthesis. Then, NH_3_ and CO yield rates were obtained to investigate the impact of reduction kinetics (NO_3_RR and CO_2_RR) on urea electrosynthesis. As shown in Fig. 3h, NH_3_ yield rates increase from 0.046 to 0.112 and 0.171 mol g_cat._^–1^ h^–1^, and CO decreases from 0.392 to 0.169 and 0.115 mol g_cat._^–1^ h^–1^ for Pd-Ni(OH)_2_, Pd_1_Cu_1_-Ni(OH)_2_ and Pd_4_Cu_1_-Ni(OH)_2_ at –0.5 V, respectively. Surprisingly, the ratio of NH_3_:CO yield rates for Pd_4_Cu_1_-Ni(OH)_2_ is 1.5, approaching the theoretical value of 2 in urea. The result clarifies the matched kinetics of NO_3_RR and CO_2_RR contributes the high urea yield rate and FE in C–N coupling process.

### Theoretical calculations

**Fig. 4:**
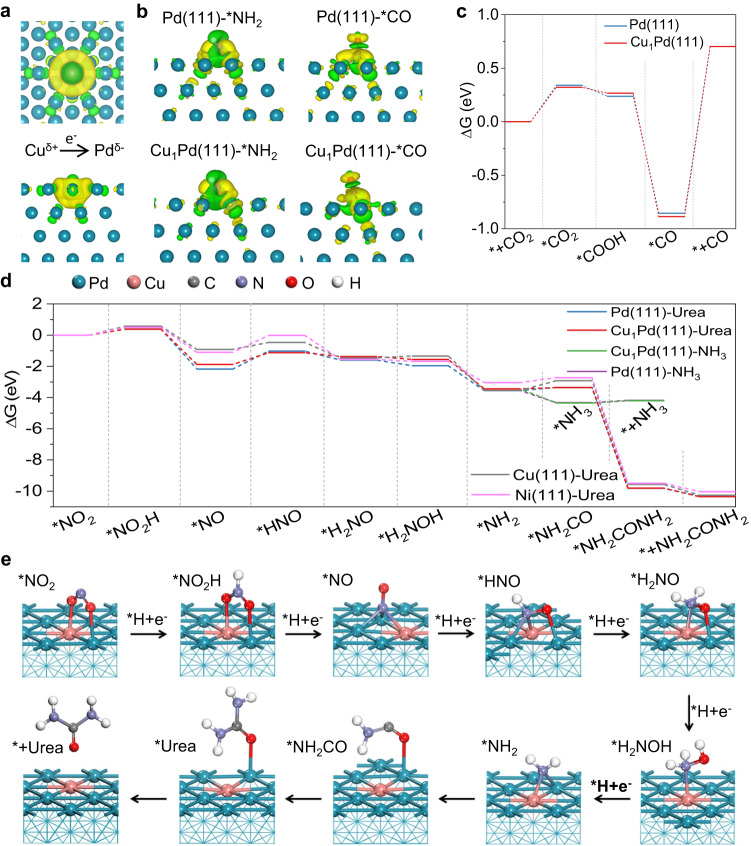
Theoretical calculations. **a** Differential charge density of Cu_1_Pd(111) (top view: top, side view: down). The isosurface value of yellow contour is 0.001 e/bohr^3^. **b** Differential charge density of Cu_1_Pd(111)-*NH_2_, Pd(111)-*NH_2_, Cu_1_Pd(111)-*CO, Pd(111)-*CO. The isosurface values of yellow contour are 0.002 or 0.00157 e/bohr^3^, respectively. **c** Energy profiles of each elementary step in single CO_2_RR catalyzed by Cu_1_Pd(111) and Pd(111) planes. **d** Energy profiles of each elementary step in NO_3_RR with C–N coupling toward urea synthesis catalyzed by Cu_1_Pd(111), Pd(111), Cu(111) and Ni(111) planes. **e** DFT-calculated urea synthesis cycle on Cu_1_Pd(111) surface.

Then, density functional theory calculations were carried out to reveal the promotion effect of Cu single-atom alloy on urea electrosynthesis. According to the HRTEM result, single-atom Cu alloyed Pd(111) (denoted as Cu_1_Pd) and Pd(111) planes were employed as the slabs. Differential charge density plots of Cu_1_Pd(111) (Fig. 4a) indicate that the electrons of Cu are delocalized and donated to Pd atoms around Cu atom due to higher electronegativity of Pd atoms^42^. Bader charge analysis confirms Cu atom denotes 0.21 e^–^ to adjacent Pd atoms on Cu_1_Pd(111) plane, while Pd(111) plane still shows balanced electron distribution (Supplementary Fig. 33). Given C- and N-intermediates for C–N coupling, *NH_2_ is nucleophilic and *CO is electrophilic. Therefore, *NH_2_ prefers to adsorb on Cu sites while *CO on Pd sites. To confirm this conclusion, differential charge density plots of Pd(111)-*NH_2_, Pd(111)-*CO, Cu_1_Pd(111)-*NH_2_ and Cu_1_Pd-*CO were obtained (Fig. 4b). The results indicate that *NH_2_ bonded to Pd-Cu atoms exhibits larger electron transfer, indicating strong tendency to bond. The adsorption energy also supports this conclusion (*NH_2_ on Cu: –2.59 eV, *CO on Cu: –2.16 eV). Similarly, *CO tends to adsorb on adjacent two Pd atoms (Supplementary Figs. 34–36).

To further understand the promotion effect of Cu single-atom alloy on urea electrosynthesis, we firstly derived the free-energy diagram (∆G) of reaction profile for each elementary step in CO_2_RR. As shown in Fig. 4c, CO_2_ adsorption on the catalyst surface and desorption of *CO are two endothermic processes, the later possesses larger energy barrier which is potential-determining step (PDS) for CO_2_RR to CO (Supplementary Table 5). Cu_1_Pd(111) plane lowers energy barrier of CO_2_ adsorption process and lifts the ∆G of *CO desorption process. It means that Cu single-atom alloy facilitates the conversion of CO_2_ to *CO, but restrains *CO desorption from catalyst surface. As such, C–N coupling is promoted and CO FE is declined. Then, the free-energy diagram in electrochemical NO_3_RR was also obtained, in which *NO_2_ was selected as the initial species (Fig. 4d and Supplementary Table 6). *NO_2_ → *NO_2_H, *NO → *HNO and *NH_3_ → * + NH_3_ processes are endothermic processes. *NO → *HNO process exhibits the largest energy barrier, which is PDS step in NO_3_RR. The energy barrier is 0.74 eV on Cu_1_Pd(111) surface, much lower than that on Pd(111) surface (1.15 eV), which accounts for the preference for *NH_2_ formation on Cu single-atom alloy. The first C–N coupling process of *NH_2_ + *CO → *CONH_2_ is typically endothermic reaction. And the second C–N coupling process is exothermic reaction with large energy output up to 6.44 eV on Cu_1_Pd(111) surface. The energy barriers are 0.07 and 0.19 eV on Cu_1_Pd(111) and Pd(111) surface, respectively, which validates Cu single-atom alloy facilitates C–N coupling. The most stable adsorption configurations on Cu_1_Pd(111) and Pd(111) planes are demonstrated in Fig. 4e and Supplementary Fig. 37. Although Cu(111) planes deliver much lower energy barrier of PDS (0.45 eV), ∆G of the first C–N coupling step on Cu(111) planes is the largest, which leads to negligible urea formation on Cu nanosheets (Supplementary Fig. 38).

### Promoting urea electrosynthesis performance by optimizing the carrier

**Fig. 5:**
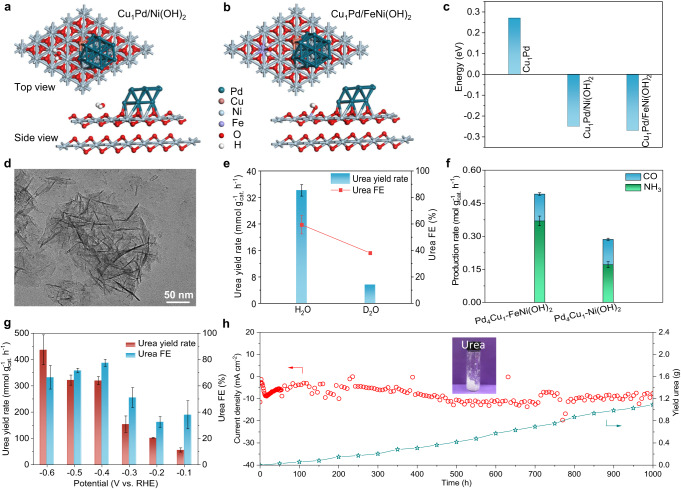
Characterization of Pd_4_Cu_1_-FeNi(OH)_2_ sample. Adsorption configurations of H_2_O on **a** Cu_1_Pd/Ni(OH)_2_ and **b** Cu_1_Pd/FeNi(OH)_2_ interface. **c** The energy barrier of dissociation of H–OH bond on Cu_1_Pd surface, Cu_1_Pd/Ni(OH)_2_ and Cu_1_Pd/FeNi(OH)_2_ interfaces. **d** TEM image of Pd_4_Cu_1_-FeNi(OH)_2_ sample. **e** Urea yield rates and urea FEs with H_2_O or D_2_O as H-source. **f** The comparison of production rates of CO and NH_3_ with Pd_4_Cu_1_-FeNi(OH)_2_ or Pd_4_Cu_1_-Ni(OH)_2_. **g** Potential-dependent urea yield rates and FEs assessed in GDE coupled with oxidation of anisyl alcohol at anode, **h** long-term *I-t* stability test and the time-resolved urea yield amount for Pd_4_Cu_1_-FeNi(OH)_2_ at –0.5 V in the mixture of 0.1 M KHCO_3_ + 0.1 M KNO_3_ using a continuous flow system in GDE with CO_2_ bubbling (20 mL min^–1^) and catalyst loading of 0.025 mg cm^–2^. Insert: the produced urea. **e**–**g** Error bars in accordance with the standard deviation of at least three independent measurements.

Upon clarifying the promotion effect of Cu single-atom alloy on urea electrosynthesis, we further uncovered the role of Ni(OH)_2_ carrier on urea electrosynthesis. First, Pd_4_Cu_1_ anchored on Ni(OH)_2_ nanosheets suppress the aggregation of clusters during long-term electrochemical process, which contributes to the good cycling stability. Second, Pd_4_Cu_1_/Ni(OH)_2_ interface facilitates the dissociation of interfacial water molecules by forming Ni^δ+^‧‧‧O^2–^H‧‧‧Pd_4_Cu_1_ interaction in alkaline electrolyte (Supplementary Fig. 39)^43,44^. As such, more active H atoms are formed on Pd_4_Cu_1_ catalyst surface, and then the following deoxyreduction processes (CO_2_ → *CO, NO_3_^–^ → *NH_2_) in urea formation are accelerated. This conclusion is confirmed by replacing Ni(OH)_2_ nanosheets with good conductors (reduced graphene oxide, rGO and XC-72) or semiconductor (TiO_2_ nanosheets) as carriers (Supplementary Figs. 40 and 41). Given the important promotion role of Ni(OH)_2_ carrier in water splitting, we infer that urea yield rate can be further improved by Fe^3+^ doping in Ni(OH)_2_ nanosheets, as high valence state of Fe^3+^ in Ni(OH)_2_ was proved to improve water splitting^45^. Theoretical calculation results reveal that water molecules indeed tend to adsorb on Ni(OH)_2_ or Fe-doped Ni(OH)_2_ surface by forming Ni–OH_2_ or Fe–OH_2_ interaction (Fig. 5a, b). As such, the energy barrier for breaking H–OH bond declines from 0.27 eV on Cu_1_Pd surface to –0.25 and –0.27 eV on Cu_1_Pd/Ni(OH)_2_ and Cu_1_Pd/FeNi(OH)_2_ interface (Fig. 5c), respectively, suggesting that water splitting is boosted on the interface. Notably, the produced active H atoms on Cu_1_Pd surface tend to combine with the adjacent *NO_3_ and *CO_2_, instead of coupling each other to release H_2_, which well explains the high urea FE for P_4_Cu_1_-Ni(OH)_2_ (Supplementary Figs. 42 and 43). Hence, Pd_4_Cu_1_ single-atom alloy clusters anchored on Fe-doped Ni(OH)_2_ composite sample was synthesized, denoted as Pd_4_Cu_1_-FeNi(OH)_2_ (Fig. 5d and Supplementary Figs. 44–47). The control experiments confirm that Fe-doped Ni(OH)_2_ nanosheets carriers are inert for CO_2_RR and have weak ability to catalyze NO_3_RR and urea formation, further verifying Pd_4_Cu_1_ clusters are the real active sites for C–N coupling (Supplementary Figs. 48–50)^46^. To confirm the enhanced water dissociation speeds up urea formation, D_2_O was employed as D-source which can slow down D-OD dissociation and D transfer processes due to isotope effect^47^. As shown in Fig. 5e, urea yield rate and urea FE are declined to 1/6 with D_2_O as D-source. As such, the kinetics of CO_2_RR and NO_3_RR are enhanced after Fe^3+^ doping in Ni(OH)_2_ nanosheets, which is validated by both improved NH_3_ and CO yield rates (Fig. 5f).

As expected, urea yield rate reaches 63.5 mmol g_cat._^–1^ h^–1^ with FE of 59.7% in H-type cell at –0.6 V (V vs. RHE), it is approximately 3.4-fold larger than that of Pd_4_Cu_1_-Ni(OH)_2_ recorded at –0.5 V (Supplementary Fig. 51). To further maximize energy utilization efficiency, urea electrosynthesis in GDE was also assessed by coupling the oxidation of anisyl alcohol at anode (Supplementary Fig. S52)^48^. As shown in Fig. 5g, the best urea yield rate and FE reach recorded 436.9 mmol g_cat._^–1^ h^–1^ and 66.5% at –0.6 V, it is about an order of magnitude higher than the optimal urea yield rate that has been reported (Supplementary Table 3). Beyond that, Pd_4_Cu_1_-FeNi(OH)_2_ composite catalyst delivers astounding cycling stability, which can sustain continuous 1000 h test without obvious current decay (Fig. 5h). The produced amount of urea in the electrolyte is proportional to the reaction time, further confirming the rigidity of our composite catalyst. Finally, 1.05 g urea was obtained from the electrolyte (Supplementary Fig. S53).

## Discussion

In summary, highly efficient Cu single-atom alloy catalyst is synthesized for urea electrosythesis with CO_2_ and NO_3_^–^ from dynamics and thermodynamics points. In situ Raman spectroscopic results reveal the key coupling pathway of *CO + *NH_2_ → *NH_2_CO + *NH_2_ → NH_2_CONH_2_. Theoretical calculation results indicate that Cu single-atom alloy in Pd lattice facilitates the further reduction of NO_2_^–^ to NH_3_ and lowers the energy barrier for the first C–N coupling. In addition, Cu doping level and the interface of Pd_4_Cu_1_/FeNi(OH)_2_ tunes the kinetics of CO_2_RR and NO_3_RR to achieve the matched formation kinetics of *CO and *NH_2_. Taken together, Pd_4_Cu_1_-FeNi(OH)_2_ composite catalyst achieve a high urea yield rate of 436.9 mmol g_cat._^–1^ h^–1^ and 66.5% in GDE, as well as long cycling stability of 1000 h, far exceeding the reported results. This work provides an insight into catalyst design toward highly efficient, selective and robust C–N coupling from the angle of single-atom alloy.

## Methods

### Synthesis of α-Ni(OH)_2_ nanosheets

Ni(NO_3_)_2_‧6H_2_O (1.45 g) and urea (0.6 g) were firstly dissolved in a mixture of triethylene glycol (40 mL) and DI water (10 mL) to form a light green transparent solution. Then, the solution was transferred and sealed in an autoclave with a Teflon liner and was heated at 120 °C for 24 h. After it was cooled to room temperature, the product was collected by centrifugation and further soaked in ethanol for 24 h. Finally, the product was collected by centrifugation and washed with ethanol for three times, dried in a vacuum oven for 24 h.

### Synthesis of Fe-doped Ni(OH)_2_ nanosheets

Ni(NO_3_)_2_‧6H_2_O (1.45 g), urea (0.6 g) and FeCl_3_‧6H_2_O (405.5 mg) were firstly dissolved in a mixture of triethylene glycol (40 mL) and water (10 mL) to form a light yellow transparent solution. Then, the solution was transferred and sealed in an autoclave with a Teflon liner, and was heated at 120 °C for 24 h. After it was cooled to room temperature, the product was collected by centrifugation and further soaked in ethanol for 24 h. Finally, the product was collected by centrifugation and washed with ethanol for three times, and dried in a vacuum oven for 24 h.

### Synthesis of Pd_x_Cu_1_-Ni(OH)_2_ composite catalysts

In a typical synthesis of Pd_4_Cu_1_-Ni(OH)_2_ composite structure, Ni(OH)_2_ (35.3 mg) nanosheets powder was ultrasonically dispersed in 20 mL DI water for 5 min. Then, K_2_PdCl_4_ (3.13 mg) and CuCl_2_‧2H_2_O (0.4 mg) were dissolved in the above mixture solution. After that, ice water cooled NaBH_4_ solution (1.0 mM, 6 mL) was dropped in the mixture to reduce Pd^2+^ and Cu^2+^ to form Pd_4_Cu_1_ alloy cluster. After stirring for another 1 h, the final product was collected by centrifugation, washed three times with ethanol and water, and dried in a vacuum oven for 24 h. The protocol for the synthesis of Pd_*x*_Cu_1_-Ni(OH)_2_ (x = 1, 2, 3, 5, 6) was similar with that of Pd_4_Cu_1_-Ni(OH)_2_ except with Cu and Pd dosage of 1.0, 0.7, 0.5, 0.34, 0.3 mg and 2.0 2.6, 2.9, 3.3, 3.4 mg, respectively. The protocols for the synthesis of Pd_4_Cu_1_-XC-72 and Pd_4_Cu_1_-TiO_2_ were similar with that of Pd_4_Cu_1_-Ni(OH)_2_ except with XC-72 (35.3 mg) and TiO_2_ nanosheets (35.3 mg) as carriers, respectively. The protocols for the synthesis of Pd_4_Cu_1_-FeNi(OH)_2_ was similar except with FeNi(OH)_2_ nanosheets (35.3 mg) as carrier and NaBH_4_ solution (1.0 mM, 18 mL).

### Electrosynthesis of urea in H-type cell

Pd_4_Cu_1_-Ni(OH)_2_ (2 mg) was ultrasonically dispersed for 30 min in a mixture of H_2_O (0.7 mL), isopropanol (0.25 mL) and Nafion (0.05 mL, 5 wt.%) to form the catalyst ink. Then, a 50-μL aliquot of catalyst ink was coated evenly on carbon paper with an area of 1 × 1 cm^2^ (catalyst loading: 0.1 mg cm^–2^) and dried under infrared lamp, which was used as working electrode. An Ag/AgCl and Pt plate were used as reference electrode and counter electrode, respectively.

All electrochemical tests were performed in an H-type cell using three-electrode system at room temperature, in which cathode chamber and anode chamber were separated by a commercial Nafion 117 membrane. The electrolyte solution for both cathode and anode was the mixture of KHCO_3_ (40 mL, 0.1 M) and KNO_3_ (0.1 M) solution. Prior to the electrochemical test, electrolyte was bubbled with continuous ultra-high purity CO_2_ gas (99.999%) for 30 min. Electrochemical coupling of CO_2_ and NO_3_^–^ was triggered under constant potentials (–0.1, –0.2, –0.3, –0.4, –0.5, and –0.6 V, versus the reversible hydrogen electrode, RHE) with continuous CO_2_ flow. The applied potentials were all converted to the RHE scale according to the following equation:

1 $$E\left({{{{{\rm{vs}}}}}}.{{{{{\rm{RHE}}}}}}\right)=E\left({{{{{\rm{vs}}}}}}.{{{{{{\rm{Ag}}}}}}}/{{{{{{\rm{AgCl}}}}}}}\right)+0.197\,{{{{{\rm{V}}}}}}+0.059\,{{{{{\rm{V}}}}}}\times {{{{{\rm{pH}}}}}}$$

After 2 h of continuous electrolysis, the produced urea in the electrolyte at the cathode chamber was spectrophotometrically quantified with diacetylmonoxime reagent or determined by hydrogen nuclear magnetic resonance (^1^H-NMR) spectroscopy measurement. The possible liquid byproducts, e.g., NH_3_, NO_2_^–^, in the electrolyte were spectrophotometrically quantified with Nessler reagent, indophenol blue and Griess reagent, respectively. The possible gaseous byproducts, e.g., CO, CH_4_, H_2_ and N_2_, were quantified by gas chromatography (GC). The electrochemical performance for other electrocatalysts were also assessed using the similar method with Pd_4_Cu_1_-Ni(OH)_2_.

### Electrosynthesis of urea in GDE

Electrosynthesis of urea in a flow cell was performed to improve carbon dioxide mass transfer kinetics. Anisyl alcohol oxidation was coupled at anode in flow cell to further reduce overpotential. To prepare catalyst ink, Pd_4_Cu_1_-Ni(OH)_2_ (2 mg) was dispersed in a mixture of Nafion (5 wt.%, 50 µL), isopropanol (250 µL) and H_2_O (700 µL), and ultrasound for 30 min. Then the catalyst ink (50 µL) was uniformly coated on the hydrophobic carbon paper with an area of 1 × 1 cm^2^ and catalyst loading of 0.1 mg cm^–2^, which was used as working electrode. An Ag/AgCl and Pt flake were used as reference electrode and counter electrode, respectively. The electrolyte solution used at cathode was the mixture of KNO_3_ (40 mL, 0.1 M) and KHCO_3_ (0.1 M). The electrolyte solution used at anode was the mixture of KOH (50 mL, 0.1 M) and anisyl alcohol (2.5 mL). The flow rates of the electrolyte solution were all 60 mL min^–1^ both for anode and cathode, and CO_2_ was continuous pumped with a flow rate of 20 mL min^–1^. The volume of the cathode and anode chambers was 1 × 1 × 1 cm^3^. When the flow cell was successfully assembled and stably operated, electrosynthesis of urea was triggered by applying a fixed potential versus RHE at cathode. After 1 h of continuous electrolysis, the produced urea in the electrolyte was spectrophotometrically quantified with diacetylmonoxime reagent or determined by ^1^H-NMR spectroscopy measurement. The procedure for Pd_4_Cu_1_-FeNi(OH)_2_ was similar with that of Pd_4_Cu_1_-Ni(OH)_2_ sample, except with the catalyst loading of 0.025 mg cm^–2^.

### Determination of urea

#### Way 1

EDTA (0.1 g) was dissolved in urease solution (10 mL, 5 mg mL^–1^). Then, electrolyte solution (1.8 mL) was added into the above solution (0.2 mL). The final solution was reacted for 40 min at 37 °C in a shaker. The produced NH_3_ was spectrophotometrically quantified with indophenol blue method.

#### Way 2

The produced amount of urea in the electrolyte was determined by diacetylmonoxime method. Typically, 1 mL electrolyte was added into 2 mL acid-ferric solution (100 mL concentrated phosphoric acid, 300 mL concentrated sulfuric acid, 600 mL deionized water and 100 mg ferric chloride). And then 1 mL diacetylmonoxime (DAMO)-thiosemicarbazide (TSC) solution (5 g DAMO and 100 mg TSC were dissolved in 1000 mL deionized water) was added into the mixture. After that, the solution was heated to 100 °C and maintained for 20 min. After it was cooled to room temperature, UV–Vis absorption spectrum was performed and the absorbance at 525 nm was acquired. A series of standard urea solutions were used to obtain working curves for urea determination.

### Determination of ammonia (NH_3_)

#### Way 1

The produced ammonia in the electrolyte solution was spectrophotometrically quantified with Nessler reagent. Typically, the diluted electrolyte solution (5 mL) was added into seignette salt solution (100 μL, 0.2 M) to wipe off the possible metal cations contamination. Commercial Nessler reagent (150 μL) was added into the above mixture for 10 min. Absorbance at 420 nm was acquired from the UV-Vis absorption spectrum. A series of standard NH_3_ solutions were used to obtain working curve for NH_3_ determination.

#### Way 2

Sodium salicylate (5 g) and seignette salt (5 g) were dissolved in NaOH solution (100 mL, 1 M) to obtain solution A. NaClO (3.5 mL, 10–15%) was diluted in 96.5 mL DI water to obtain solution B. Sodium nitroferricyanide (0.2 g) was dissolved in 20 mL DI water to obtain solution C. To quantify NH_3_, solution A, solution B and solution C were added in turn in the diluted electrolyte solution (2 mL). After 2 h in a dark room at room temperature, absorbance at 662 nm was acquired from the UV-vis absorption spectrum. A series of standard NH_3_ solutions were used to obtain working curve for NH_3_ determination.

### Determination of nitrite ions (NO_2_^–^)

Nitrite ions were spectrophotometrically quantified with Griess reagent. Typically, Griess reagent (200 μL) was added into electrolyte solution (5 mL). Then, the solution was heated to 100 °C and maintained for 1 min. After it was cooled to room temperature, UV-Vis absorption spectrum was acquired and the absorbance at 540 nm was obtained. A series of standard NO_2_^–^ solutions were used to obtain working curve for NO_2_^–^ determination.

### Determination of N_2_, H_2_, CO and CH_4_

The amounts of N_2_, H_2_, CO and CH_4_ were quantified by gas chromatograph (GC) equipped with TCD and FID detectors.

The FEs for urea, NO_2_^–^, NH_3_, N_2_, CO, CH_4_, and H_2_ were calculated according to Eqs. (2)–(7):

2 $${{{{{{\rm{FE}}}}}}}_{{{{{{\rm{urea}}}}}}}=\, (16F\times {C}_{{{{{{\rm{urea}}}}}}}\times V)/(60.06\times Q)$$

3 $${{{{{{\rm{FE}}}}}}}_{{{{{{{\rm{NO}}}}}}}_{{2}^{-}}}=\, (2F\times {C}_{{{{{{{\rm{NO}}}}}}}_{{2}^{-}}}\times V)/(47\times Q)$$

4 $${{{{{{\rm{FE}}}}}}}_{{{{{{{\rm{NH}}}}}}}_{3}}=\, (8F\times {C}_{{{{{{{\rm{NH}}}}}}}_{3}}\times V)/(17\times Q)$$

5 $${{{{{{\rm{FE}}}}}}}_{{{{{{{\rm{N}}}}}}}_{2}}=\, (10F\times {C}_{{{{{{{\rm{N}}}}}}}_{2}}\times V/{V}_{m})/(28\times Q)$$

6 $${{{{{{\rm{FE}}}}}}}_{{{{{{\rm{CO}}}}}}}=\, (2F\times {C}_{{{{{{\rm{CO}}}}}}}\times V/{V}_{m})/(28\times Q)$$

7 $${{{{{{\rm{FE}}}}}}}_{{{{{{{\rm{CH}}}}}}}_{4}}=\, (8F\times {C}_{{{{{{{\rm{CH}}}}}}}_{4}}\times V/{V}_{m})/(16\times Q)$$

Where *F* is the Faraday constant (96485.3 C mol^–1^) and *Q* is the total charge passed through the working electrode.

CO_2_-to-urea selectivity and NO_3_^–^-to-urea selectivities were calculated according to Eqs. 8 and 9:

8 $${{{{{{\rm{N}}}}}}}_{{{{{{\rm{urea}}}}}}}-{{{{{\rm{selectivity}}}}}}={{{{{{\rm{n}}}}}}}_{{{{{{\rm{urea}}}}}}}({{{{{\rm{N}}}}}})/{{{{{{\rm{n}}}}}}}_{{{{{{\rm{total}}}}}}}({{{{{\rm{N}}}}}})$$

9 $${{{{{{\rm{C}}}}}}}_{{{{{{\rm{urea}}}}}}}-{{{{{\rm{selectivity}}}}}}={{{{{{\rm{n}}}}}}}_{{{{{{\rm{urea}}}}}}}({{{{{\rm{C}}}}}})/{{{{{{\rm{n}}}}}}}_{{{{{{\rm{total}}}}}}}({{{{{\rm{C}}}}}})$$

### Theoretical calculation

The calculations in this work were performed with the Vienna ab initio Simulation Package (VASP), calculating the exchange-correlation function via the generalized gradient approximation (GGA) within the Perdew–Burke–Ernzerhof (PBE) flavor^49,50^. The Projected Augmented Wave (PAW) method was employed to describe the core-valance electron interaction^51,52^. The kinetic energy cutoff of 400 eV for plane-wave basis was set, and the reciprocal space was sampled by a 3 × 3 × 1 Monkhorst–Pack grid of size. The 4 × 4 Pd(111) surface slabs were constructed with four layers (bottom two layers fixed), with vacuum layers of at least 15 Å to avoid the vertical interactions. The convergence criteria are 10^–5^ eV and 0.05 eV/Å for energy differences and atomic remaining force, respectively.

The binding energy is defined as *E*_Binding_ = *E*_A@Sub_–*E*_Sub_ − *E*_A_, where *E*_*A@Sub*_ is the total energy of an *A* intermediate adsorbed over the substrate, *E*_*Sub*_ and *E*_*A*_ are the entire energy of one single *A* adsorbate and substrate in vacuum. The computational hydrogen electrode (CHE) model was applied for the simulation of the proton-coupled electron (H^+^+e^–^) transfer process via simplified the proton-coupled electron-transfer step to (H^+^+e^–^→1/2H_2_). DFT calculated free energies (*G*) were corrected according to *G* = *E*_DFT_ + *E*_ZPE_ − *TS* (298.15 K), where *E*_DFT_ is the calculated total energy for each step, *E*_ZPE_ is the zero-point energy and *S* is the entropic contribution.

### Sample characterizations

Prior to electron microscopy characterizations, a drop of the suspension of nanostructures in ethanol was placed on a piece of carbon-coated copper grid and dried under ambient conditions. Transmission electron microscopy (TEM), high-resolution TEM (HRTEM) images and the corresponding energy-dispersive X-ray spectroscopy (EDS) mapping profiles were taken on a JEOL JEM-2100F field-emission high-resolution transmission electron microscope operated at 200 kV. Powder X-ray diffraction (XRD) patterns were recorded on a Philips X’Pert Pro Super X-ray diffractometer with Cu-Kα radiation (*λ* = 1.5418 Å). X-ray photoelectron spectra (XPS) were collected on an ESCALab 250 X-ray photoelectron spectrometer with nonmonochromatized Al-Kα X-ray as the excitation source. The concentrations of Pd and Cu were measured with a Thermo Scientific PlasmaQuad 3 inductively coupled plasma mass spectrometry (ICP-MS) after dissolving the samples with a mixture of HCl and HNO_3_ (3:1, volume ratio). In situ Raman spectroscopy was performed with the Raman microscopy system (WITEC alpha300 R confocal Raman system) using 633 nm He–Ne laser as the excitation source.

### Supplementary information

Supplementary Information

Peer Review File

## Data Availability

The authors declare that all data supporting the findings of this study are available in the article and its Supplementary Information.
