# Supplementary material for: Kinetically matched C–N coupling toward efficient urea electrosynthesis enabled on copper single-atom alloy
Source: Nat Commun. 2023 Nov 1;14:6994. doi: 10.1038/s41467-023-42794-2 (PMC10620222; doi:10.1038/s41467-023-42794-2)
Supplement: Supplementary file 1 — Supplementary Information [file 41467_2023_42794_MOESM1_ESM.pdf]

Kinetically matched C–N coupling toward efficient urea electrosynthesis enabled on copper single-atom alloy

Mengqiu Xu[1,‡], Fangfang Wu[2,‡], Ye Zhang[1], Yuanhui Yao[1], Genping Zhu[1], Xiaoyu Li[1], Liang Chen[1*], Gan Jia[1], Xiaohong Wu[3*], Youju Huang[1], Peng Gao[1*] and Wei Ye[1*]

[1]College of Material, Chemistry and Chemical Engineering, Key Laboratory of Organosilicon Chemistry and Material Technology, Ministry of Education, Hangzhou Normal University, Hangzhou, Zhejiang 311121, China.

[2]College of Materials Science and Engineering, Zhejiang University of Technology, Hangzhou, Zhejiang 310014, China.

[3]School of Chemistry and Chemical Engineering, Harbin Institute of Technology, Harbin, Heilongjiang 150001, P. R. China.

[‡]These authors contributed equally.

Chemicals.

Nickel(II) nitrate hexahydrate ($Ni(NO_3)_2·6H_2O$, A.R.), copper(II) chloride dihydrate ($CuCl_2·2H_2O$, A.R.), potassium palladium(II) chloride (K_2PdCl_4, A.R.), iron(III) trichloride hexahydrate ($FeCl_3·6H_2O$, A.R.), tetrabutyl titanate ($C_{16}H_{36}O_4Ti$, A.R.), diacetylmonoxime ($C_4H_7NO_2$, A.R.), thiosemicarbaxide (CH_5N_3S, A.R.), urease, urea (CH_4N_2O, A.R.), carbon black, sodium borohydride ($NaBH_4$, A.R.), sodium salicylate ($NaC_7H_5O_3$, A.R.), Monopotassium monosodium tartrate tetrahydrate ($KNaC_4H_{12}O_{10}$, A.R.), sodium hydroxide (NaOH, A.R.), sodium hypochlorite (NaClO, A.R.), EDTA disodium salt dehydrate ($C_{10}H_{20}N_2Na_2O_{10}$, A.R.), sodium nitroprusside ($FeNa_2C_5H_4N_6O_3$, A.R.), 4-methoxybenzyl alcohol ($C_8H_{10}O_2$, A.R.), triethylene glycol ($C_6H_{14}O_4$, A.R.), ammonium chloride (NH_4Cl, A.R.), sodium nitrite ($NaNO_2$, A.R.), ethanol (EtOH, A.R.), ^{15}N-labeled potassium nitrate ($K^{15}NO_3$, ≥99.5%), potassium nitrate (KNO_3, A.R.), potassium hydroxide (KOH, A.R.), potassium bicarbonate ($KHCO_3$, A.R.), ultra-high purity CO_2 (99.999%), ultra-high purity Ar (99.999%), Nessler reagent and Griess reagent were purchased from Sinopharm Chemical Reagent Co. Ltd. (Shanghai, China). All chemicals were used without further purification. All aqueous solutions were prepared using de-ionized (DI) water with a resistivity of 18.25 $M\Omega·cm^{-1}$.

Synthesis of TiO_2 nanosheets

Tetrabutyl titanate (10 mL) was added in an autoclave with a Teflon liner. Then HF solution (4 mL) was added and stirred for 10 min. After that, absolute ethanol (10 mL) was then added into the mixture and stirred for another 10 min. The mixture was transferred and sealed in an autoclave with a Teflon liner, and heated at 180 °C for 16 h. After it was cooled to room temperature, the product was collected with centrifugation and washed with NaOH (0.1 M) solution and DI water at least five times. The product was dried a vacuum oven at 80 °C for 24 h.[S1]

Synthesis of Pd_4Cu_1-rGO composite structure

Graphene oxide (15.35 mL, 2.3 wt.‰) was diluted into 20 mL DI water. Then, K_2PdCl_4 (3.13 mg) and $CuCl_2·2H_2O$ (0.4 mg) were dissolved in the above solution. After ultrasound for 10 min, ice water cooled $NaBH_4$ solution (10 mM, 6 mL) was dropped in the mixture to reduce Pd^{2+} and Cu^{2+} to form Pd_4Cu_1 alloy cluster. After stirring for another 1 h, the mixture was transferred and sealed in an autoclave with a Teflon liner, heated at 110 °C for 3 h. The final product was collected by centrifugation, washed three times with water, and freeze-dried.

Supplementary Figures:

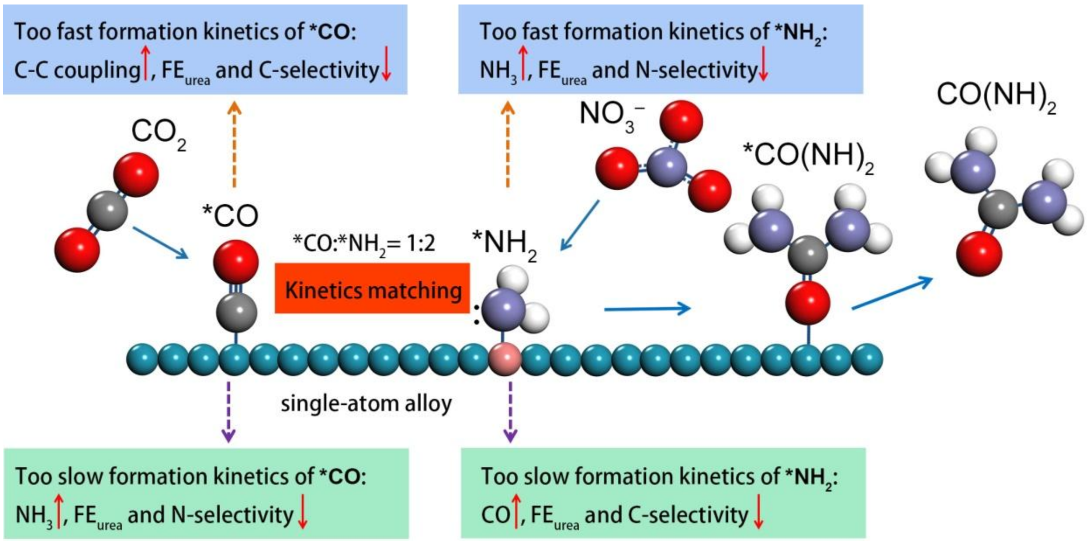

Supplementary Fig. 1. Schematic demonstration of kinetics matching of CO_2RR and NO_3RR in C–N coupling toward urea electrosynthesis.

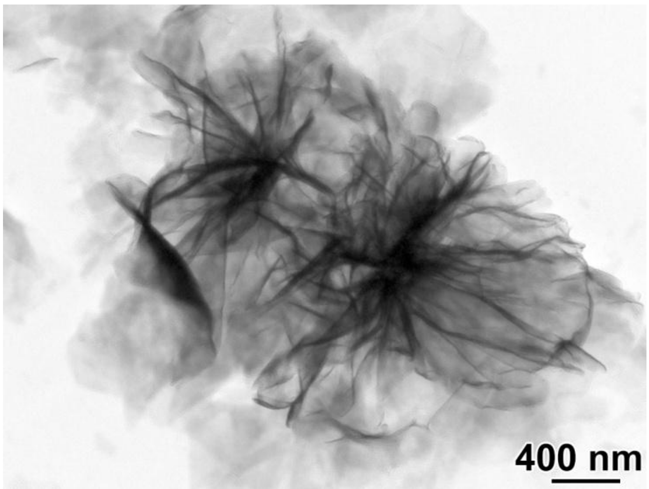

Supplementary Fig. 2. TEM image of layered α-$Ni(OH)_2$ nanosheets.

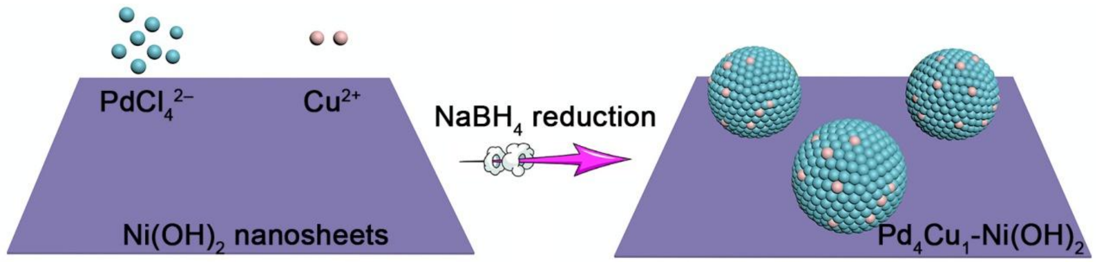

Supplementary Fig. 3. Schematic depiction of the synthesis of Pd_4Cu_1-$Ni(OH)_2$ composite structure.

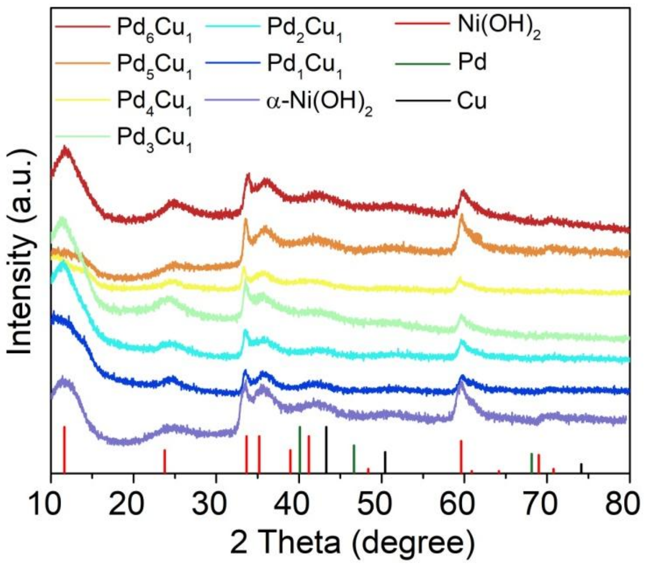

Supplementary Fig. 4. Powder XRD patterns of bare α-$Ni(OH)_2$ nanosheets, Pd_1Cu_1-$Ni(OH)_2$, Pd_2Cu_1-$Ni(OH)_2$, Pd_3Cu_1-$Ni(OH)_2$, Pd_4Cu_1-$Ni(OH)_2$, Pd_5Cu_1-$Ni(OH)_2$, and Pd_6Cu_1-$Ni(OH)_2$ composite samples. The standard diffraction patterns for α-$Ni(OH)_2$ (JCPDS No. 22-0444), *fcc* Pd (JCPDS No. 46-1043) and *fcc* Cu (JCPDS No. 04-0836) are provided as references.

Notes: As shown in Supplementary Fig. 4, the composite samples (Pd_xCu_1-$Ni(OH)_2$, x=1, 2, 3, 4, 5, 6) only display diffraction patterns of α-$Ni(OH)_2$, and the diffraction patterns of metallic Pd/Cu are not emerged. The possible reason may be due to the small size of Pd_xCu_1 clusters.

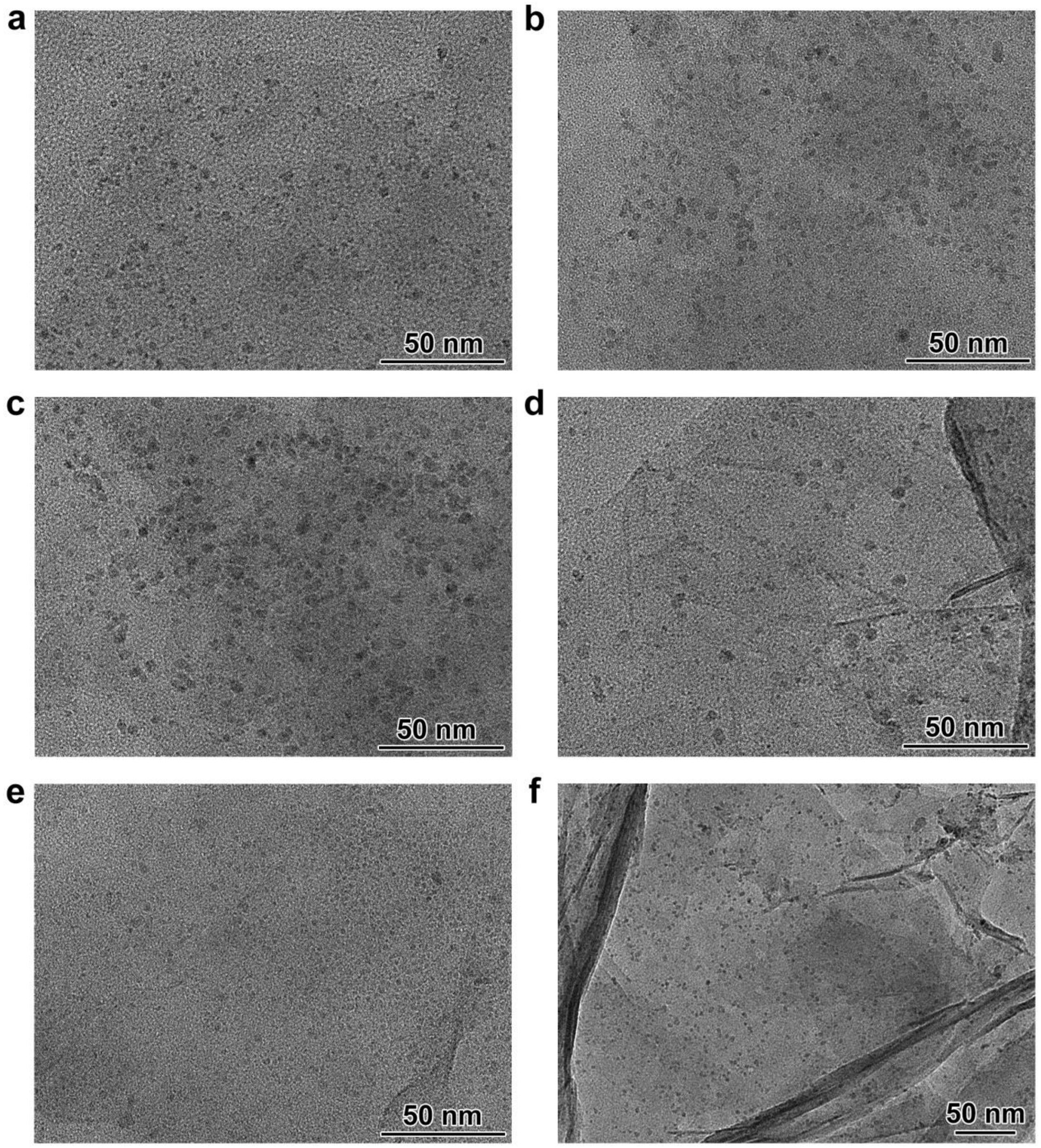

Supplementary Fig. 5. TEM images of (a) Pd_1Cu_1-$Ni(OH)_2$, (b) Pd_2Cu_1-$Ni(OH)_2$, (c) Pd_3Cu_1-$Ni(OH)_2$, (d) Pd_5Cu_1-$Ni(OH)_2$, (e) Pd_6Cu_1-$Ni(OH)_2$ and (f) Pd-$Ni(OH)_2$ composite samples.

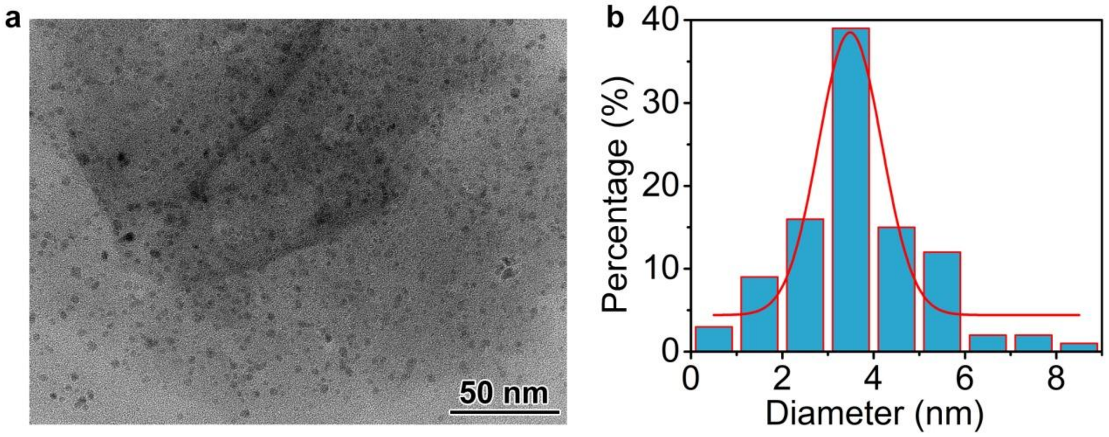

Supplementary Fig. 6. (a) TEM image of Pd_4Cu_1-$Ni(OH)_2$ composite sample. (b) The particle size distribution diagram of Pd_4Cu_1 clusters.

Notes: As shown in Supplementary Fig. 5 and 6, Pd_xCu_1 clusters (x=1, 2, 3, 4, 5, 6) anchored on α-$Ni(OH)_2$ display comparable size distribution, which is a prerequisite for the comparison of catalytic activity.

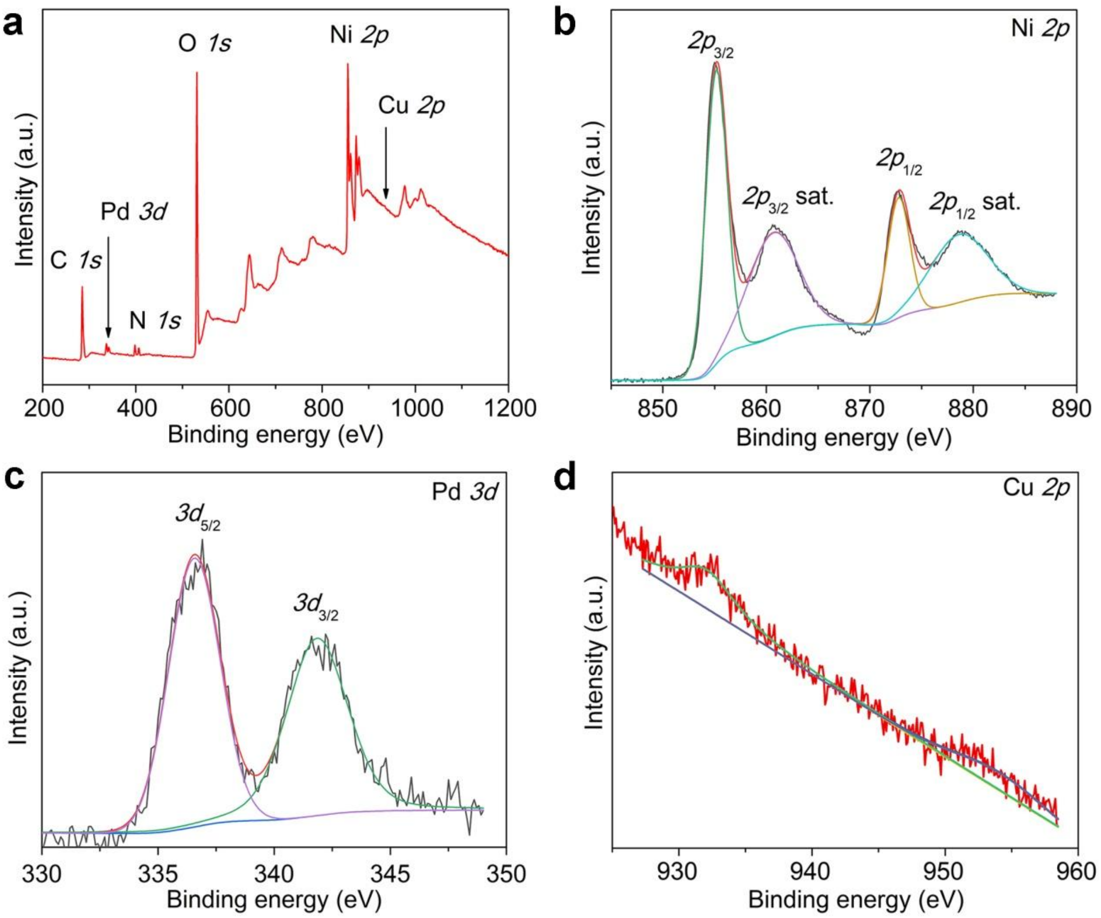

Supplementary Fig. 7. XPS characterizations of Pd_4Cu_1-$Ni(OH)_2$ sample: (a) survey, (b) Ni *2p*, (c) Pd *3d*, (d) Cu *2p*.

Notes: As shown in Supplementary Fig. 7, the survey spectrum confirms the existence of Pd, Cu, Ni and O elements. The binding energies located at 855.2 and 872.9 eV can be assigned to $2p_{3/2}$ and $2p_{1/2}$ of Ni^{2+}, respectively (Supplementary Fig. 7b). Another pairs of binding energies located at 860.8 and 879.0 eV are assigned to the satellite peaks of $2p_{3/2}$ and $2p_{1/2}$, respectively.[S2]

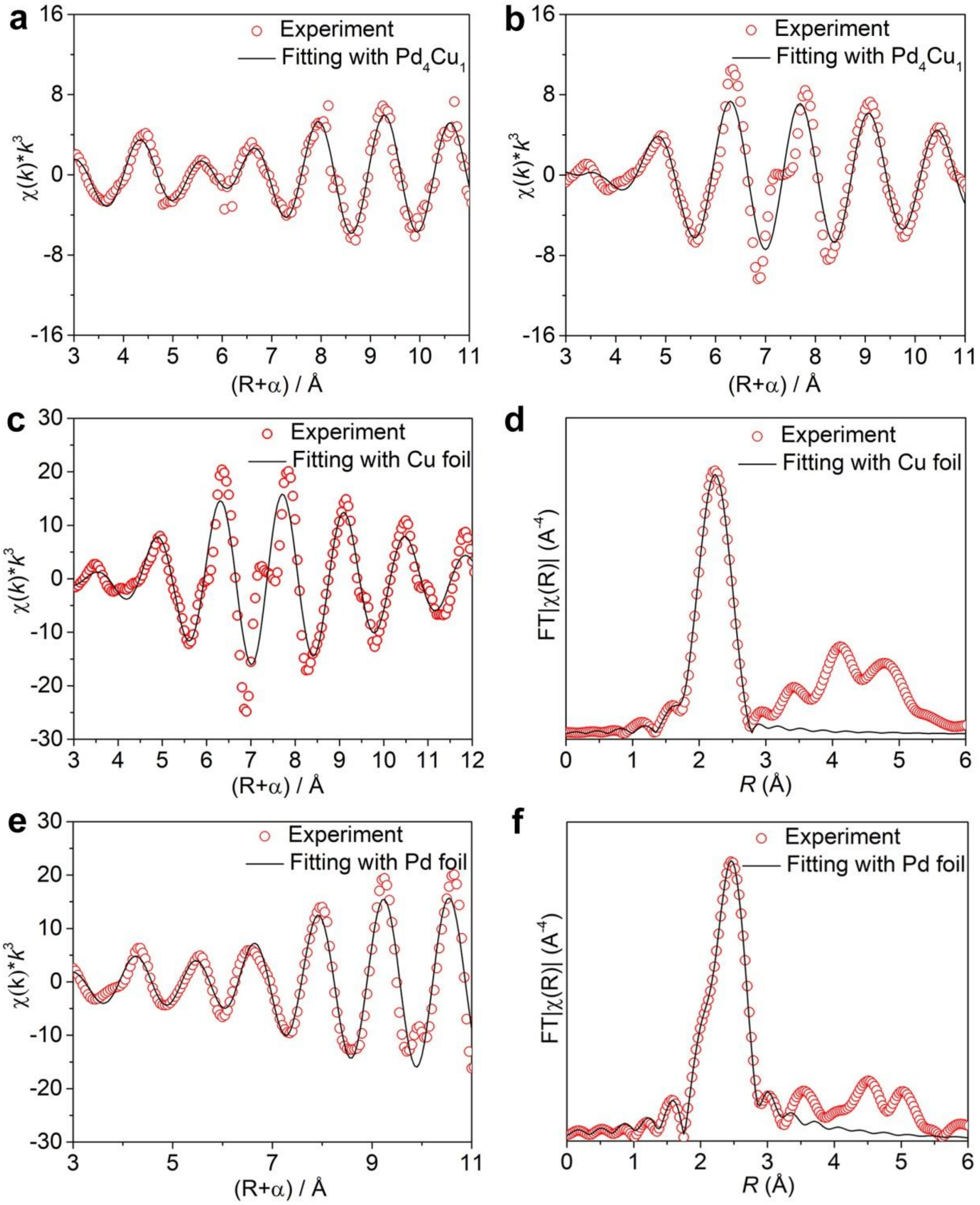

Supplementary Fig. 8. (a,b) The corresponding EXAFS *k* space fitting curves of Pd_4Cu_1-$Ni(OH)_2$ sample. The corresponding EXAFS fitting curves of (c,d) Cu foil and (e,f) Pd foil.

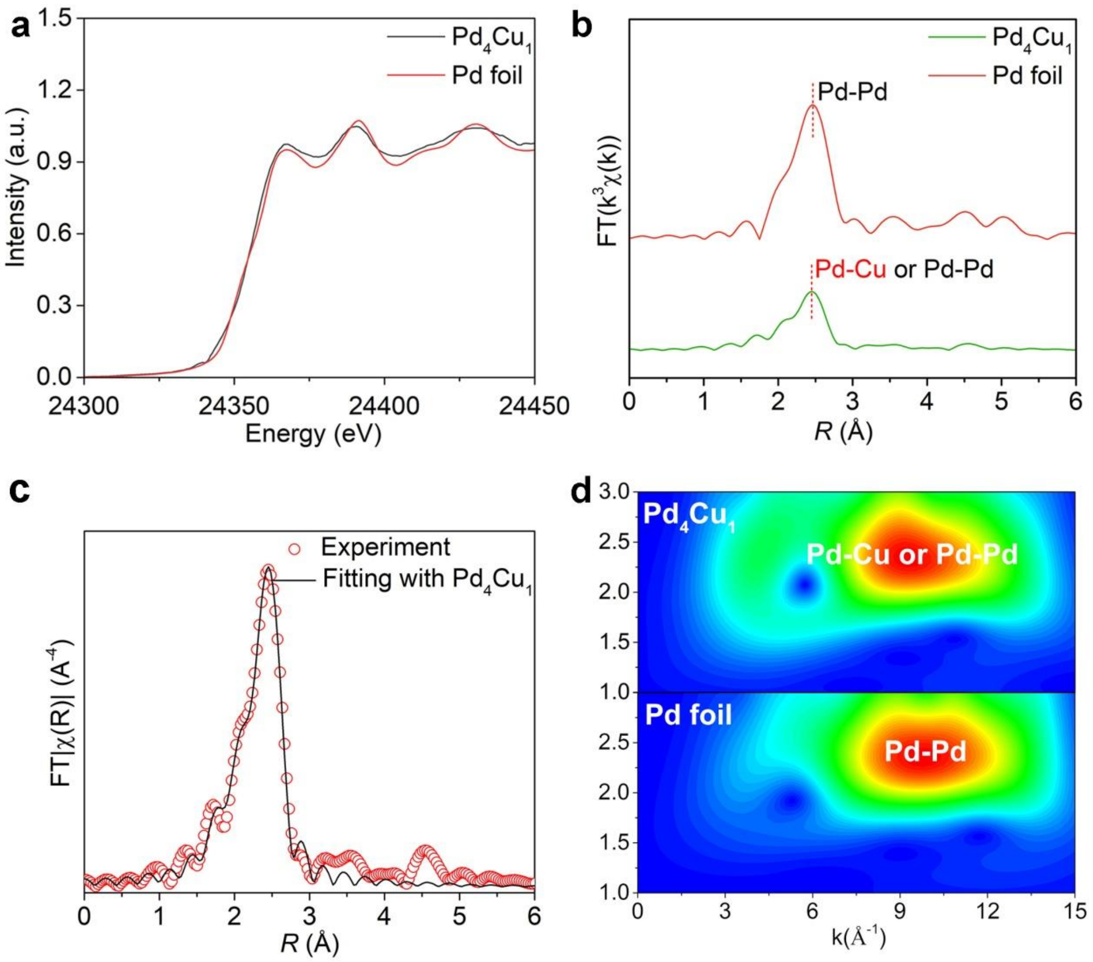

Supplementary Fig. 9. (a) Normalized Pd K-edge XANES spectra of Pd_4Cu_1 in reference with Pd foil, (b) k^3-weighted Fourier-transform Pd K-edge EXAFS spectra, (c) the experimental Pd K-edge EXAFS spectrum (red circle) and the fitting curve (black line) of Pd_4Cu_1. (d) Wavelet transforms of the k^2-weighted Pd K-edge EXAFS signals for the high-coordination shells in reference with Pd foil.

Notes: Pd K-edge XANES spectrum of Pd_4Cu_1-$Ni(OH)_2$ was also obtained in reference with Pd foil (Supplementary Fig. 9). The two curves are almost overlapped, indicating metallic Pd feature in Pd_4Cu_1-$Ni(OH)_2$ sample. Pd–Pd (2.70 Å) and Pd–Cu (2.62 Å) bonds are all resolved with CNs of 7.9 and 1.4 in Pd K-edge EXAFS (Supplementary Table 2), respectively. Consistent with Cu case, the fitting curve is almost overlapped with experiment spectrum, validating the reliability of the fitting result (Supplementary Fig. 8 and 9c). Wavelet transforms (WT) analysis of the Pd K-edge EXAFS oscillations of Pd_4Cu_1-$Ni(OH)_2$ sample resolves Pd–Cu bond (Supplementary Fig. 9d).

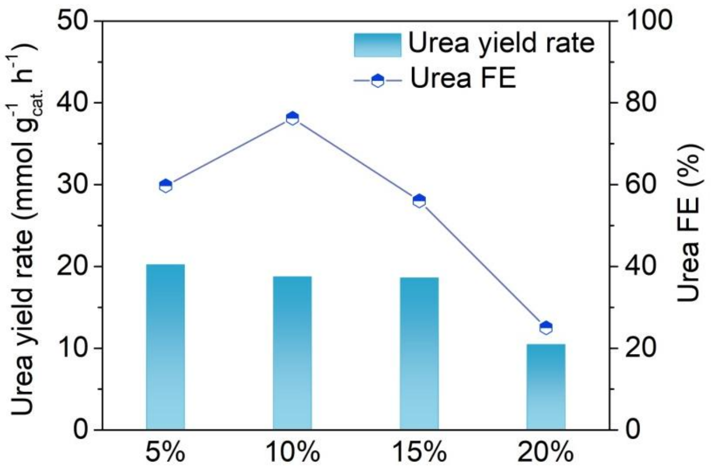

Supplementary Fig. 10. The relationship of urea yield rates and FEs with varied loading amounts of Pd_4Cu_1 in Pd_4Cu_1-$Ni(OH)_2$ composite sample at –0.5 V in H-type cell.

Notes: We investigated the effect of loading amount of Pd_4Cu_1 clusters on urea yield rate and FE at –0.5 V. As shown in Supplementary Fig. 10, urea yield rates are comparable with 5%, 10% and 15% loading amount. Urea FEs show a volcano plot with the loading amounts, and the optimal urea yield rate and urea FE were obtained with Pd_4Cu_1 loading amount of 10%.

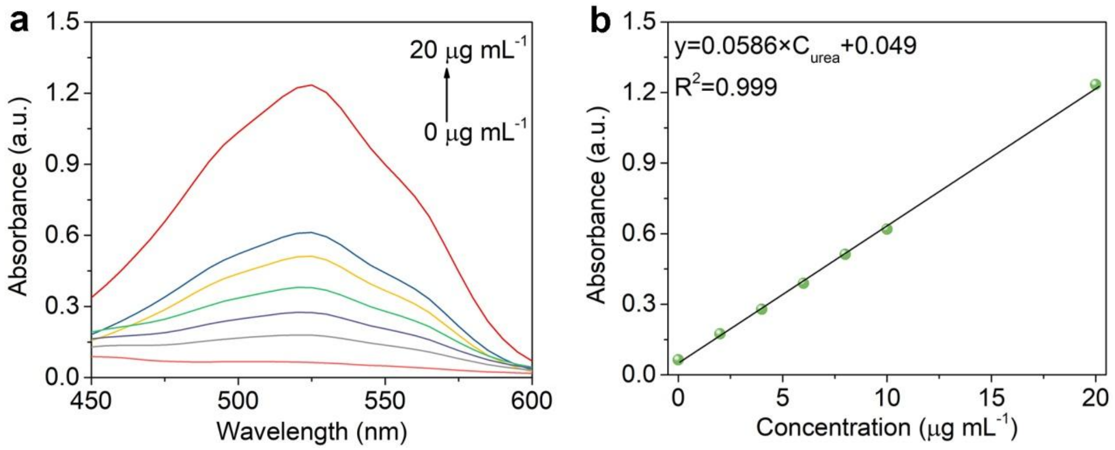

Supplementary Fig. 11. (a) UV-Vis absorption curves of diacetyl monoxime assays with varied concentrations of urea after heating at 100 ℃ for 20 min. (b) Calibration curve used for urea estimation.

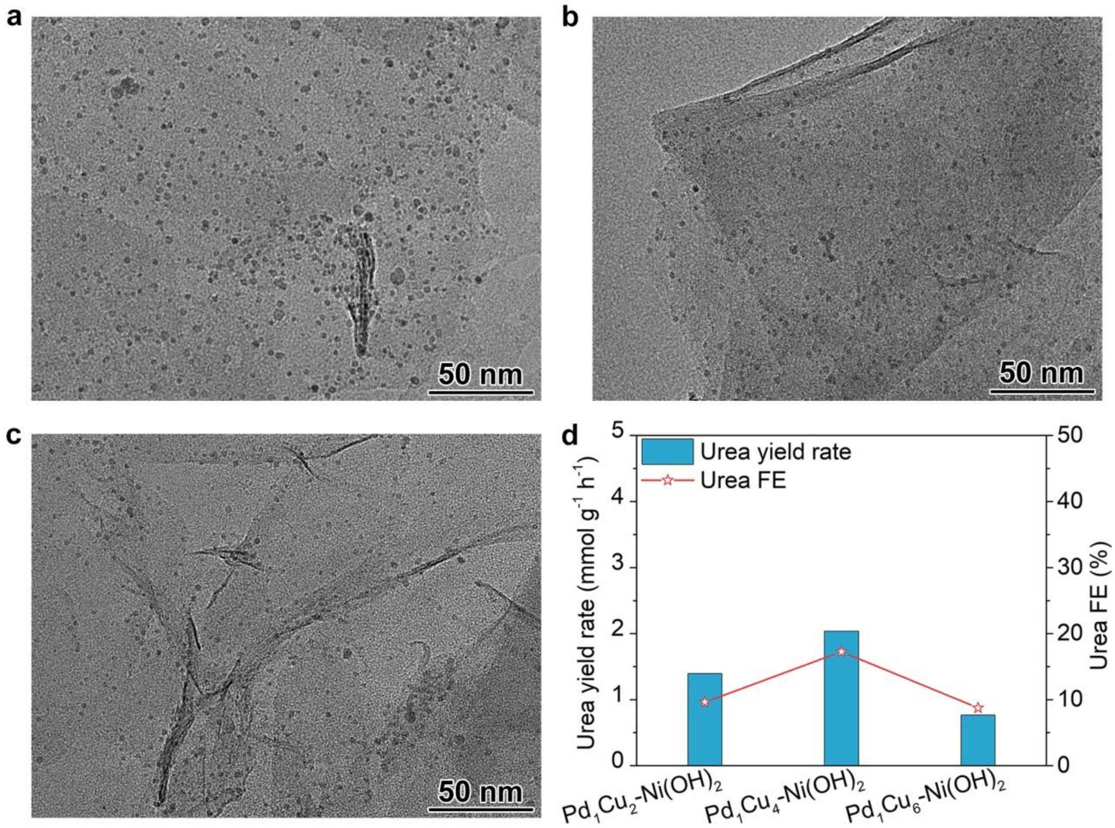

Supplementary Fig. 12. (a-c) TEM images of Pd_1Cu_2-$Ni(OH)_2$, Pd_1Cu_4-$Ni(OH)_2$, and Pd_1Cu_6-$Ni(OH)_2$ samples. (d) Urea yield rates and FEs of the three samples.

Notes: To further demonstrate the unique role of Cu single-atom in Pd host, we also prepared Pd single-atom in Cu lattice as a comparison. As shown in Supplementary Fig. 12a-12c, Pd_1Cu_2, Pd_1Cu_4 and Pd_1Cu_6 clusters are successfully anchored on $Ni(OH)_2$ nanosheets. Urea yield rates and urea FEs are 0.77, 2.03, 1.39 mmol g^{-1} h^{-1} and 8.7%, 17.3%, 9.6% for Pd_1Cu_6-$Ni(OH)_2$, Pd_1Cu_4-$Ni(OH)_2$ and Pd_1Cu_2-$Ni(OH)_2$ composite catalysts (Supplementary Fig. 12d), respectively. Urea yield rates and urea FEs all lower than that of Pd_4Cu_1-$Ni(OH)_2$, suggesting the unique role of Cu single-atom alloy in promoting urea electrosynthesis.

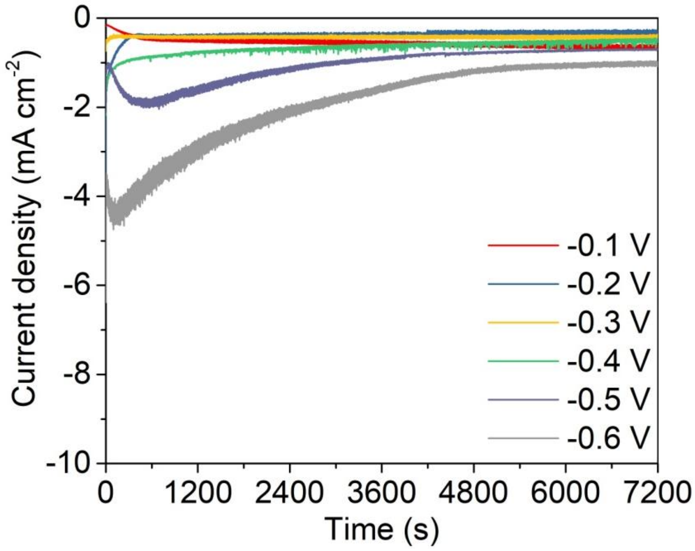

Supplementary Fig. 13. Potential-dependent *I*-*t* curves of Pd_4Cu_1-$Ni(OH)_2$ sample in the mixture of $KHCO_3$ (0.1 M) and KNO_3 (0.1 M) in H-type cell under continuous CO_2 flow.

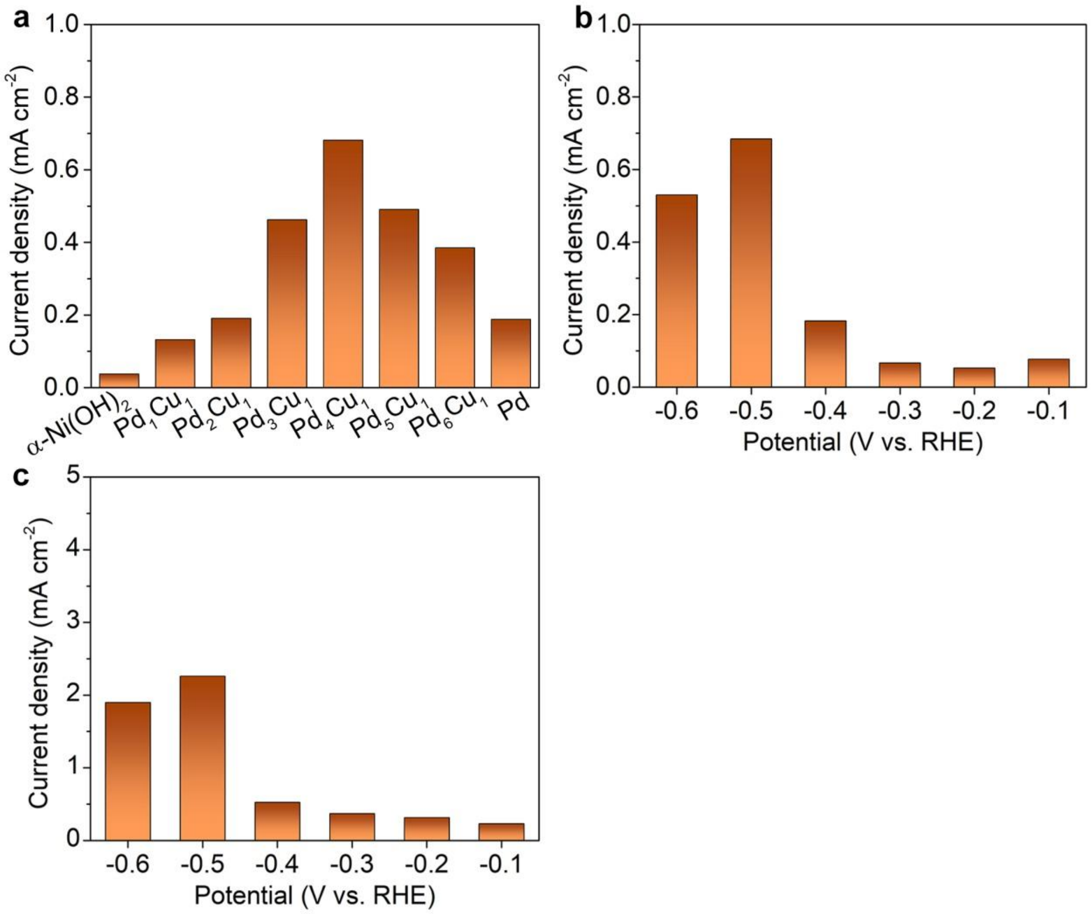

Supplementary Fig. 14. Urea partial current densities for (a) Pd_xCu_1-$Ni(OH)_2$ (x=1-6) composite samples at –0.5 V, (b) Pd_4Cu_1-$Ni(OH)_2$ in H-type cell and (c) in GDE.

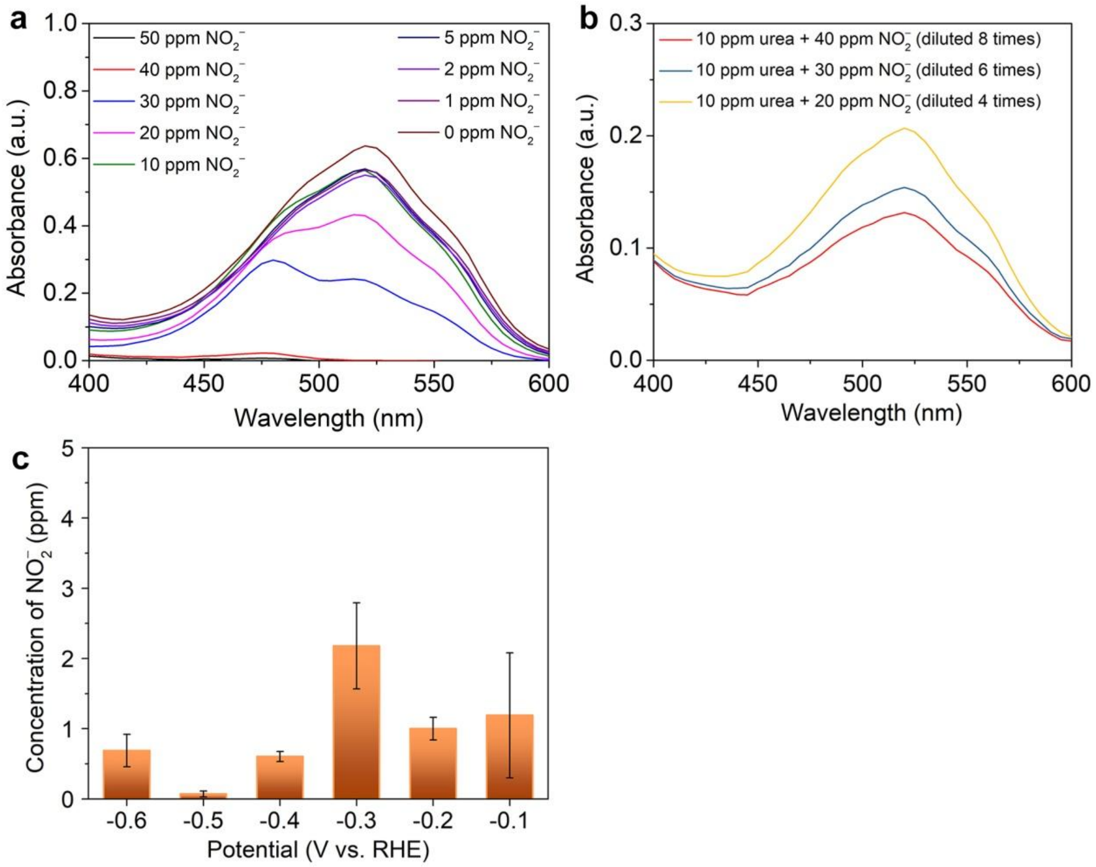

Supplementary Fig. 15. (a) UV-Vis absorption curves of urea (10 ppm) with different concentrations of NO_2^-, (b) UV-Vis absorption curves of the diluted solutions. (c) Potential-dependent concentrations of NO_2^- in the electrolyte. (c) Error bars in accordance with the standard deviation of at least three independent measurements.

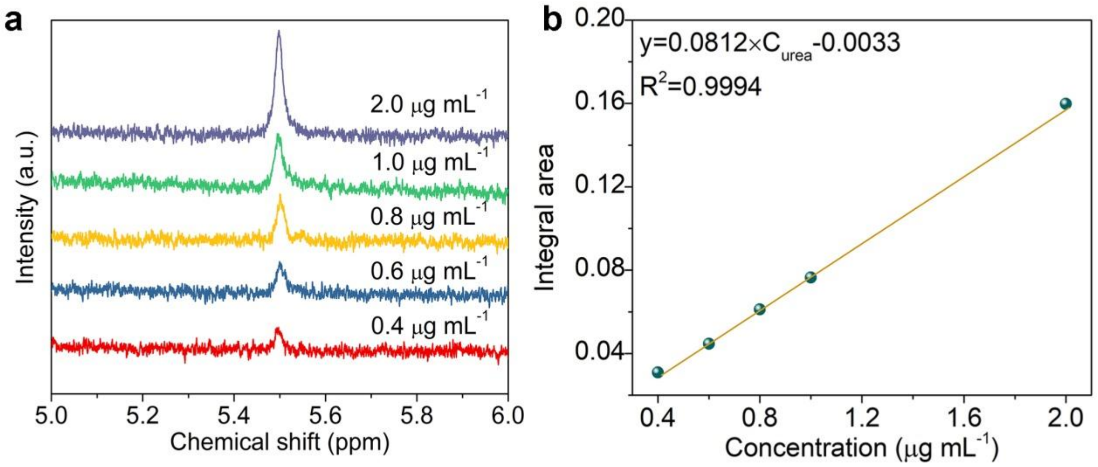

Supplementary Fig. 16. (a) ^{1}H-NMR spectra (500 MHz) recorded in a series of urea solutions with different concentrations. (b) Calibration curve used for urea quantification.

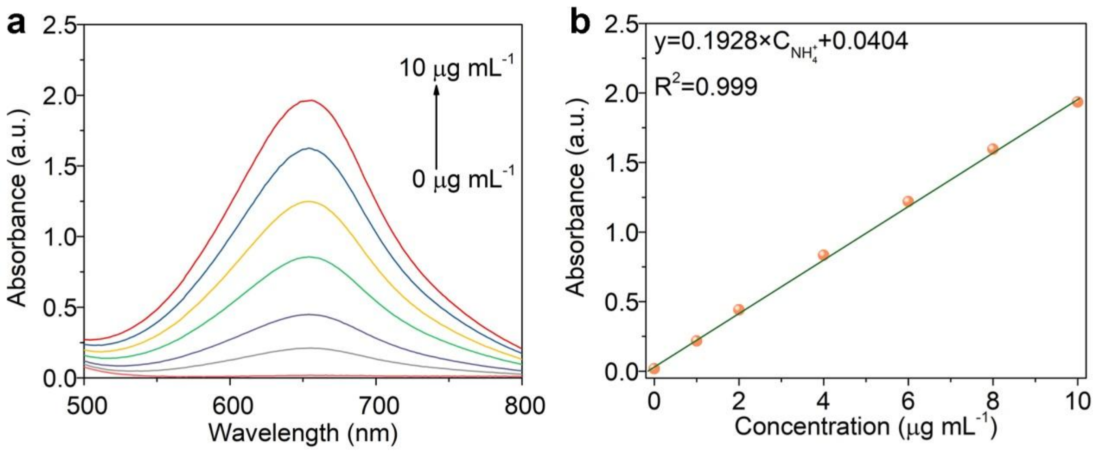

Supplementary Fig. 17. UV-Vis curves of indophenol assays with NH_4^+ ions and (b) calibration curve used for estimation of NH_3.

Notes: In order to investigate the impact of NO_2^- on the quantification of urea in the electrolyte, we firstly obtained UV-Vis absorption curves of 10 ppm urea with different concentrations of NO_2^-. As shown in Supplementary Fig. 15a, the absorbance at 524 nm is really declined when the concentrations of NO_2^- surpass 10 ppm. As the concentrations of NO_2^- decline to 10, 5, 2, 1 ppm, the absorption curves are almost overlapped, but slightly lower than that without NO_2^-. The results indicate that NO_2^- will not greatly affect the quantification of urea when the concentrations are less than 10 ppm. This conclusion is further confirmed by diluting results (Supplementary Fig. 15b). Therefore, we also quantified the concentrations of NO_2^- in the electrolyte. As shown in Supplementary Fig. 15c, the concentrations of NO_2^- in the electrolyte all lower than 3 ppm, suggesting the accuracy of spectrophotometric result. In order to further confirm the concentration of urea in the electrolyte, the quantification of urea were further carried out with ^{1}H-NMR (Supplementary Fig. 16) and urease method (Supplementary Fig. 17). The results for the estimation of urea concentrations are consistent.

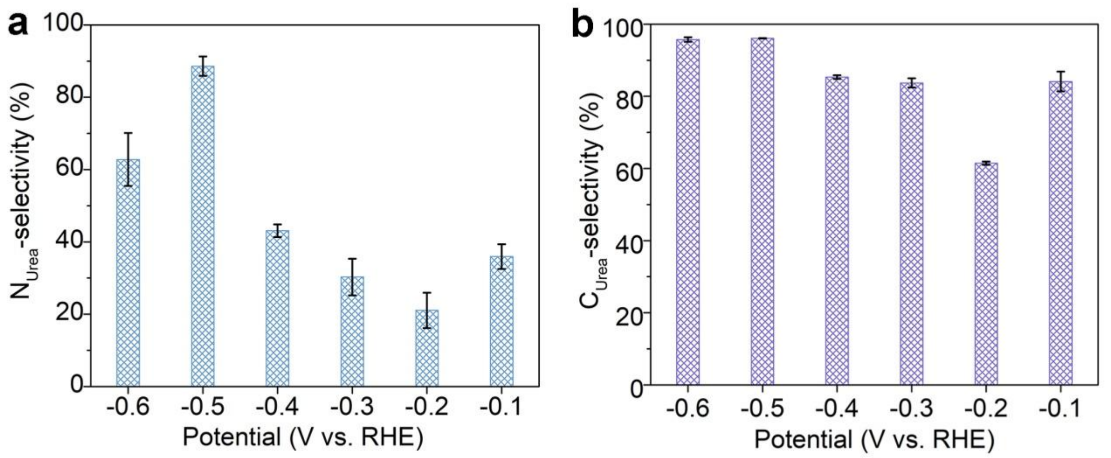

Supplementary Fig. 18. Potential-dependent (a) NO_3^--to-urea and (b) CO_2-to-urea selectivity for Pd_4Cu_1-$Ni(OH)_2$ composite sample in urea electrosynthesis. (a, b) Error bars in accordance with the standard deviation of at least three independent measurements.

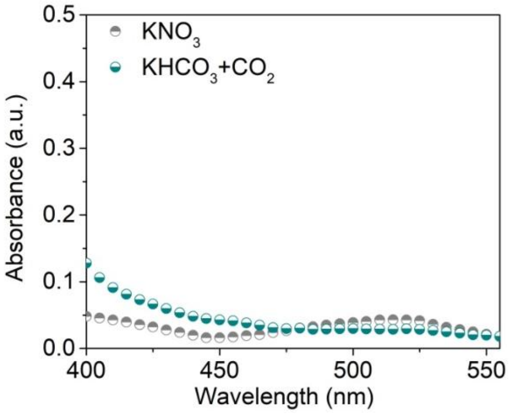

Supplementary Fig. 19. UV-Vis spectra of the electrolytes for Pd_4Cu_1-$Ni(OH)_2$ in the mixture of KNO_3 or $KHCO_3$ and CO_2.

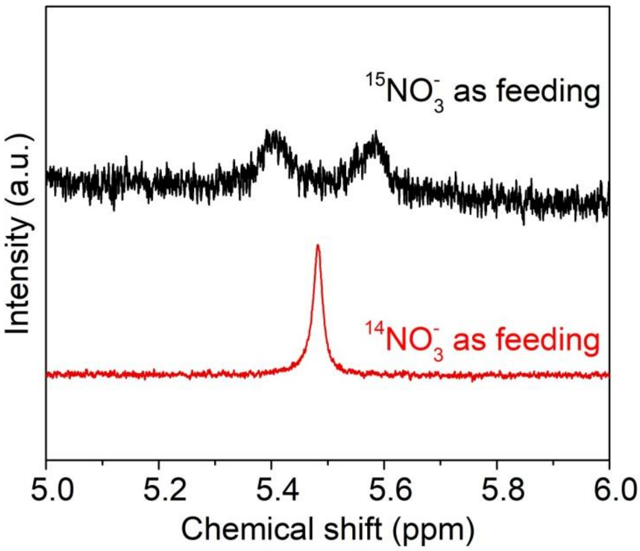

Supplementary Fig. 20. ^{15}N isotope labeling experiments using $^{15}NO_3^-$ (100 mM) and $^{14}NO_3^-$ as N feeding in electrochemical C–N coupling toward urea production.

Notes: To confirm the produced urea rooted from the electrochemical C–N coupling from CO_2 and NO_3^-, we acquired UV-Vis absorption curves for Pd_4Cu_1-$Ni(OH)_2$ recorded in solo KNO_3 or $KHCO_3$ + CO_2 at –0.5 V, which indicates no urea formation (Supplementary Fig. 19). Furthermore, ^{15}N isotope labeling experiments were carried out (Supplementary Fig. 20). ^{1}H-NMR spectra indicate that a single peak located at 5.5 ppm using $^{14}NO_3^-$ as N-source. When feeding $^{15}NO_3^-$ as N-source, a double peak is observed, which is the characteristic peak of $CO(^{15}NH_2)_2$.[S3] All the results verify that urea is really obtained from electrochemical C–N coupling from CO_2 and NO_3^-.

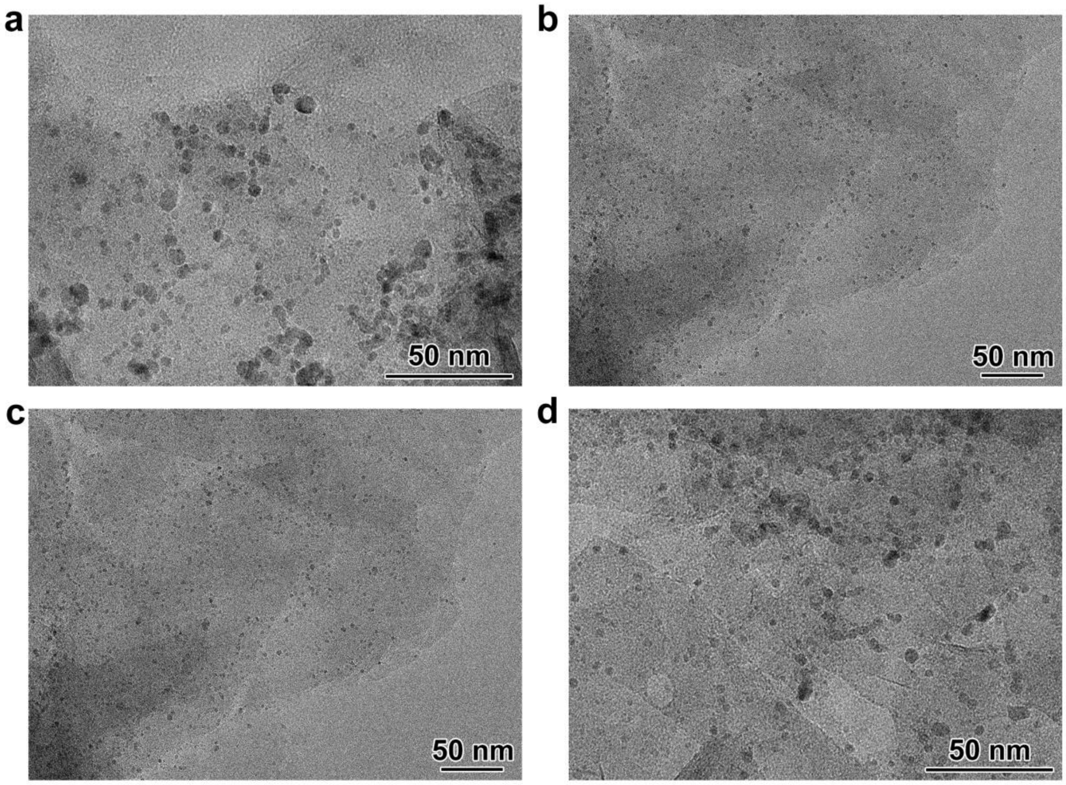

Supplementary Fig. 21. TEM images of (a) Pd_4Fe_1-$Ni(OH)_2$, (b) Pd_4Co_1-$Ni(OH)_2$, (c) Pd_4Ni_1-$Ni(OH)_2$, (d) Pd_4Zn_1-$Ni(OH)_2$.

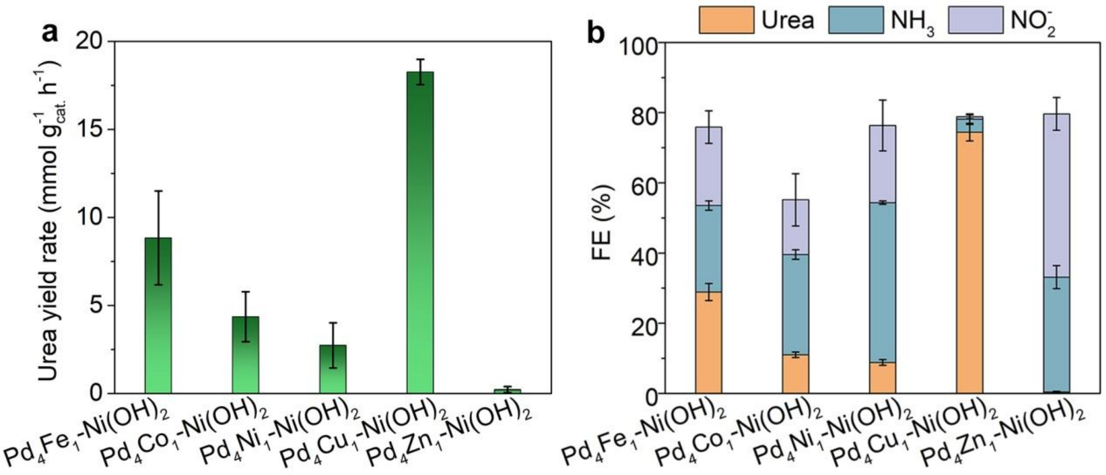

Supplementary Fig. 22. Screening of metal types (Fe, Co, Ni, Cu, Zn) in electrochemical C–N coupling: (a) Urea yield rate and (b) FE. (a, b) Error bars in accordance with the standard deviation of at least three independent measurements.

Notes: In order to screen the optimal transition metals single-atom alloys, we chose typical transition metals, i.e., Fe, Co, Ni, Cu, Zn. As shown in Supplementary Fig. 22, urea yield rates are arranged as follows: Pd_4Cu_1-$Ni(OH)_2$ > Pd_4Fe_1-$Ni(OH)_2$ > Pd_4Co_1-$Ni(OH)_2$ > Pd_4Ni_1-$Ni(OH)_2$ > Pd_4Zn_1-$Ni(OH)_2$. Urea FEs for Pd_4X_1-$Ni(OH)_2$ samples show a similar trend with urea yield rates. The results suggest the best choice of Cu single-atom alloy in urea electrosynthesis. Notably, the process of NO_2^- to *NH_2 is inhibited on Zn single-atom alloy and no urea is formed.

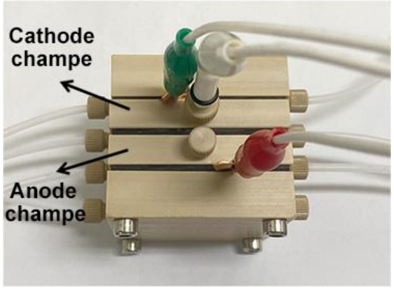

Supplementary Fig. 23. Photograph of an actual GDE for urea electrosynthesis.

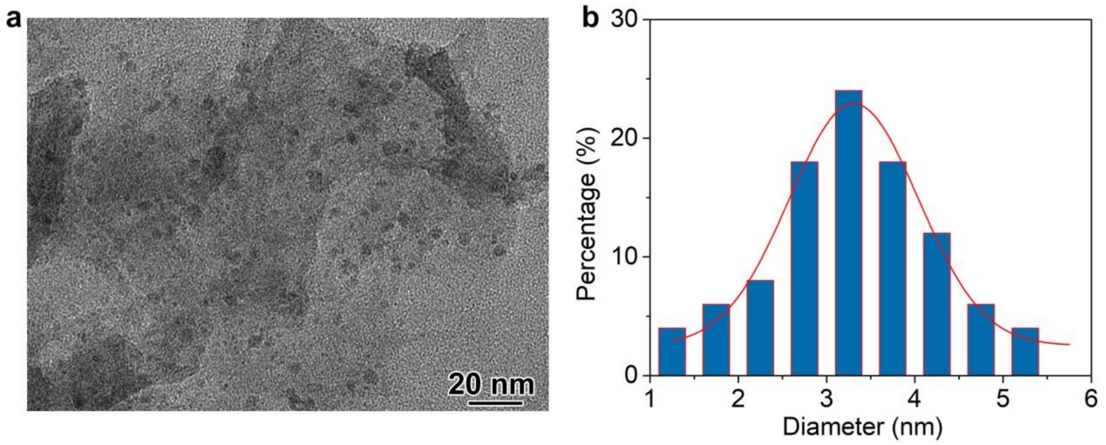

Supplementary Fig. 24. TEM image of Pd_4Cu_1-$Ni(OH)_2$ after durability test, (b) the particle size distribution diagram of Pd_4Cu_1 clusters after durability test.

Notes: Supplementary Fig. 24a shows the TEM image of Pd_4Cu_1-$Ni(OH)_2$ sample after 100 h of durability test in H-type cell. Pd_4Cu_1 still displays cluster structure with size of 3.3 ± 0.9 nm (Supplementary Fig. 24b), suggesting the rigidity of the composite catalyst.

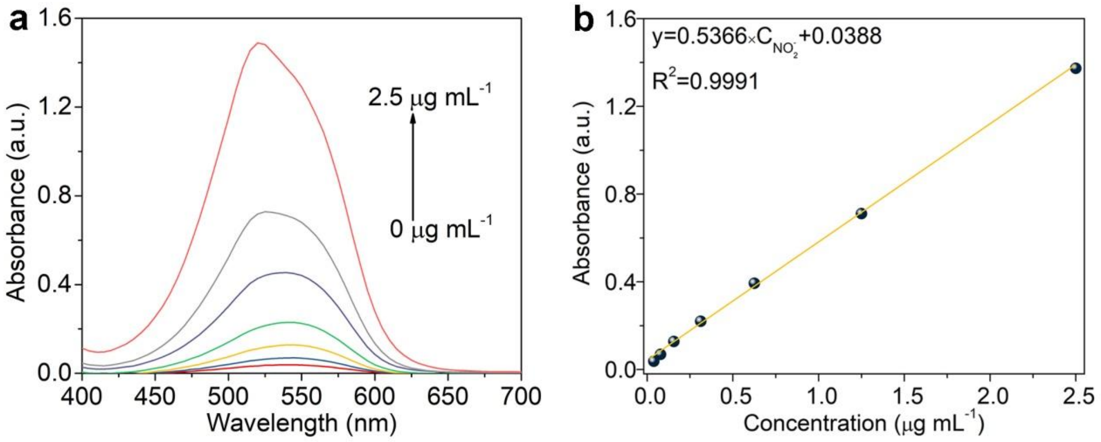

Supplementary Fig. 25. (a) UV-Vis curves of Griess's regent with varied concentrations of NO_2^- at room temperature for 15 min. (b) Calibration curve used for estimation of NO_2^-.

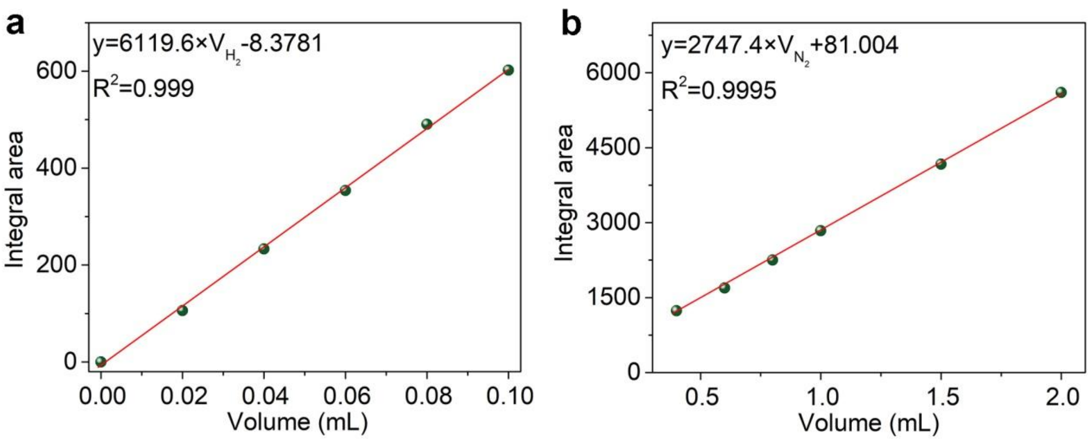

Supplementary Fig. 26. Calibration curves used for estimation of (a) H_2 and (b) N_2.

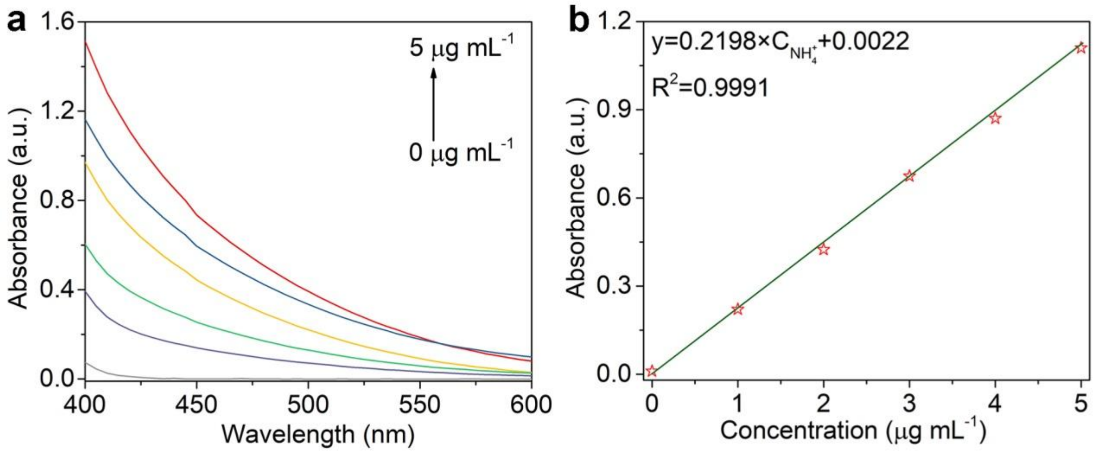

Supplementary Fig. 27. (a) UV-Vis curves of Nessler's agent assays with varied concentrations of NH_4^+. (b) Calibration curve used for the estimation of NH_4^+.

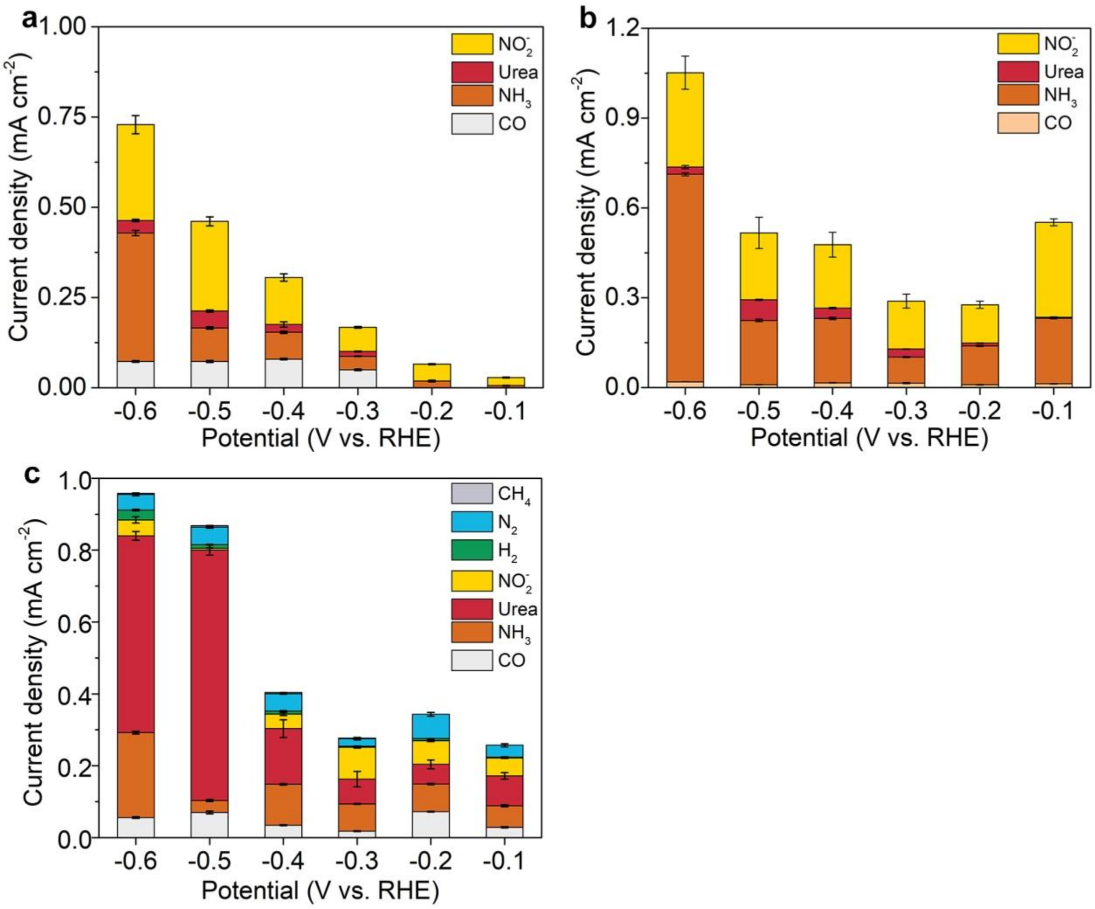

Supplementary Fig. 28. Partial current densities of the possible by-products for (a) Pd-Ni(OH)$_2$, (b) Pd$_1$Cu$_1$-Ni(OH)$_2$, (c) Pd$_4$Cu$_1$-Ni(OH)$_2$. (a-c) Error bars in accordance with the standard deviation of at least three independent measurements.

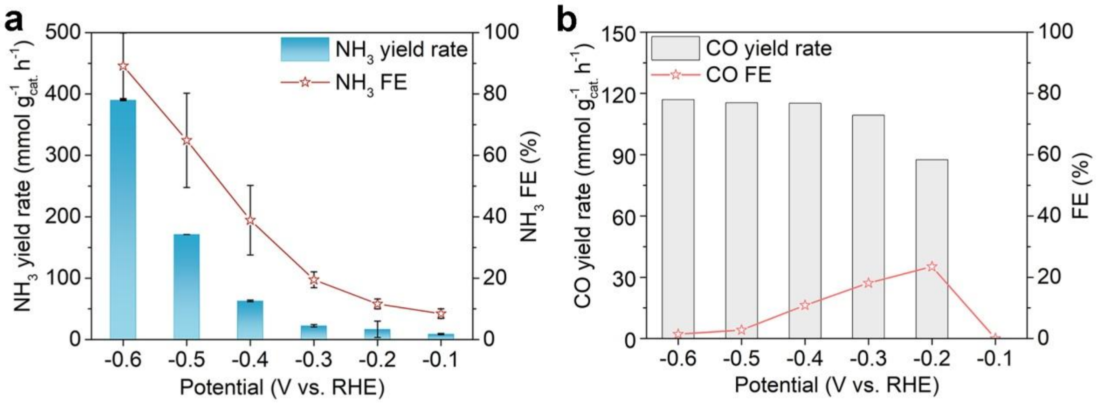

Supplementary Fig. 29. (a) Evaluation of NO_3RR performance in 0.1 M KNO_3 and (b) CO_2RR performance in 0.1 M $KHCO_3$ solution with continuous CO_2 flow using Pd_4Cu_1-$Ni(OH)_2$ sample as a catalyst in H-type cell. (a) Error bars in accordance with the standard deviation of at least three independent measurements.

Notes: Electrochemical NO_3RR performance was assessed in 0.1 M KNO_3 solution (Supplementary Fig. 29a). Ammonia yield rates increase from 9.0, 16.7, 22.4, 62.9, 171.0 to 390.0 mmol $g_{cat.}^{-1}$ h^{-1} as the applied potential negatively shifts from –0.1 to –0.6 V. Accordingly, ammonia FEs increase from 8.5%, 11.6%, 19.5%, 38.9%, 64.9% to 89.2%. Supplementary Fig. 29b shows potential-dependent CO_2RR performance in H-type cell. CO is the main product in CO_2RR for Pd_4Cu_1-$Ni(OH)_2$. The yield rates of CO are 0, 87.6, 109.4, 115.2, 115.4, 117.0 mmol $g_{cat.}^{-1}$ h^{-1} at –0.1, –0.2, –0.3, –0.4, –0.5 and –0.6 V, respectively. The optimal CO FE of 23.5% was achieved at –0.2 V. (a) Error bars in accordance with the standard deviation of at least three independent measurements

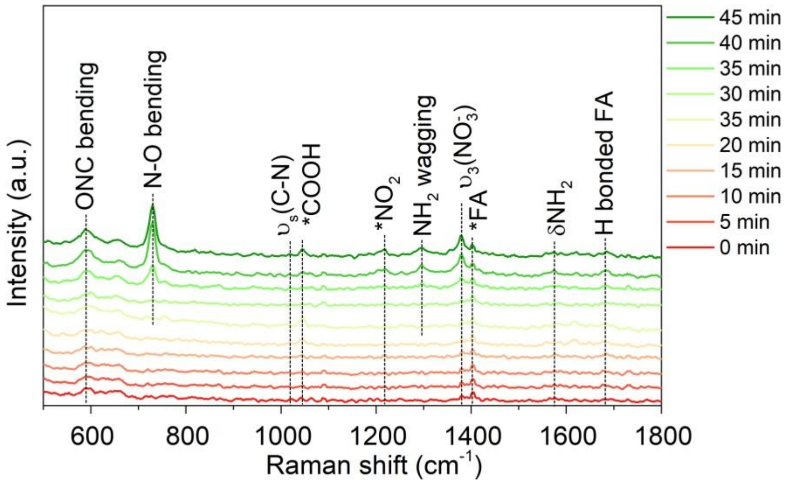

Supplementary Fig. 30. In-situ Raman spectra recorded in urea electrosynthesis at –0.5 V from 0 to 45 min for Pd_1Cu_1-$Ni(OH)_2$.

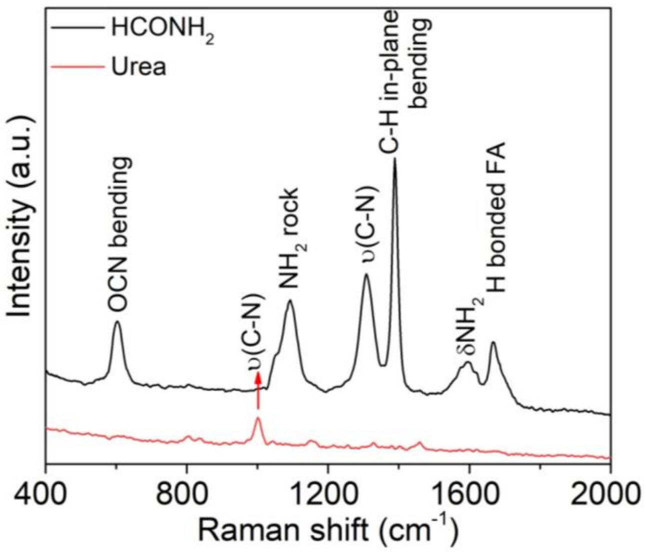

Supplementary Fig. 31. Raman spectra of $HCONH_2$ (FA) and urea standards with same concentrations dissolved in the mixture of KNO_3 and $KHCO_3$.

Notes: Supplementary Fig. 31 shows the Raman spectra of urea and $HCONH_2$ dissolved in the mixture of KNO_3 and $KHCO_3$. The peak located at 1000 cm^{-1} is the characteristic ν_s(C–N) mode of urea. As shown in Supplementary Table 4, the peaks located at 602, 1091, 1308, 1390, 1598, 1668 cm^{-1} are assigned to OCN bending, NH_2 rock, ν(C–N), C–H in-plane bending, δNH_2 modes and H bonded formamide, respectively. It should be noted that formamide displays stronger Raman signal than urea.

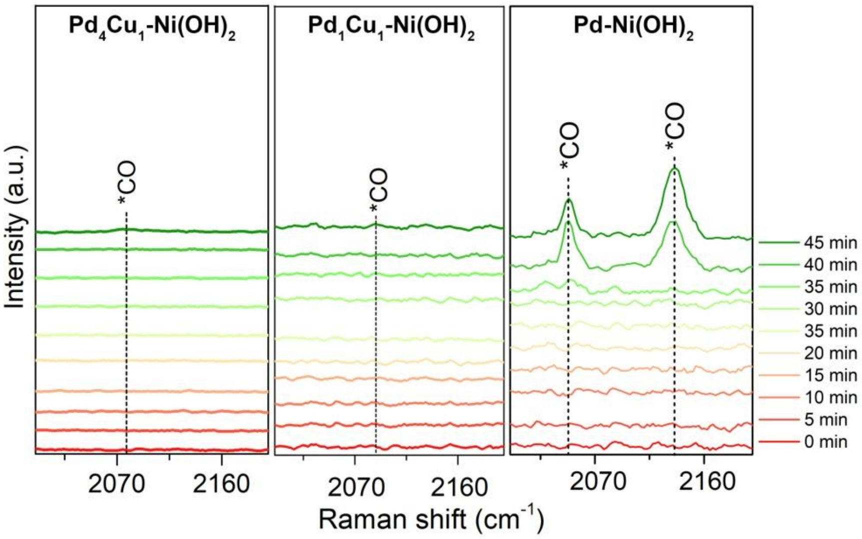

Supplementary Fig. 32. In-situ Raman spectra to trace *CO evolution on Pd-Ni(OH)$_2$, Pd$_1$Cu$_1$-Ni(OH)$_2$ and Pd$_4$Cu$_1$-Ni(OH)$_2$ at –0.5 V.

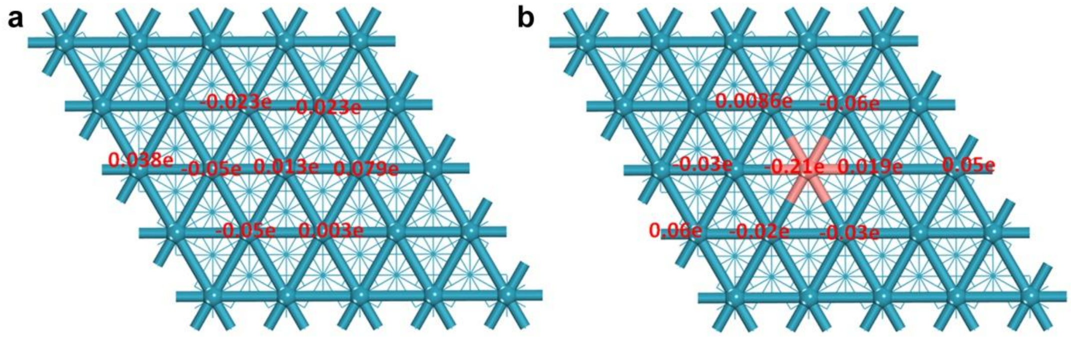

Supplementary Fig. 33. Bader charge analysis of (a) Pd(111) and (b) $Cu_1Pd(111)$ slabs.

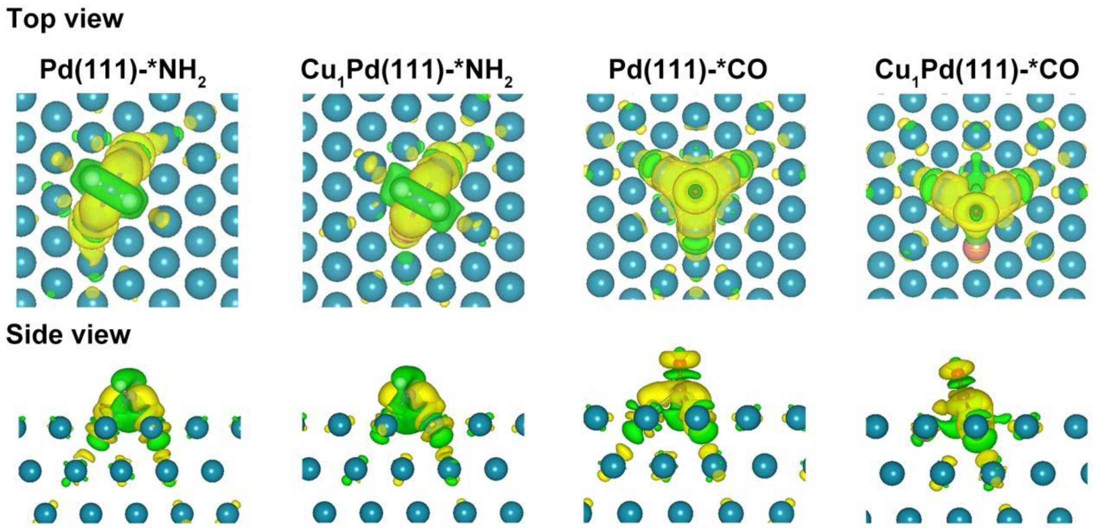

Supplementary Fig. 34. Top view (top) and side views (down) of differential charge densities of $Cu_1Pd(111)$-*NH_2, Pd(111)-*NH_2, $Cu_1Pd(111)$-*CO, Pd(111)-*CO.

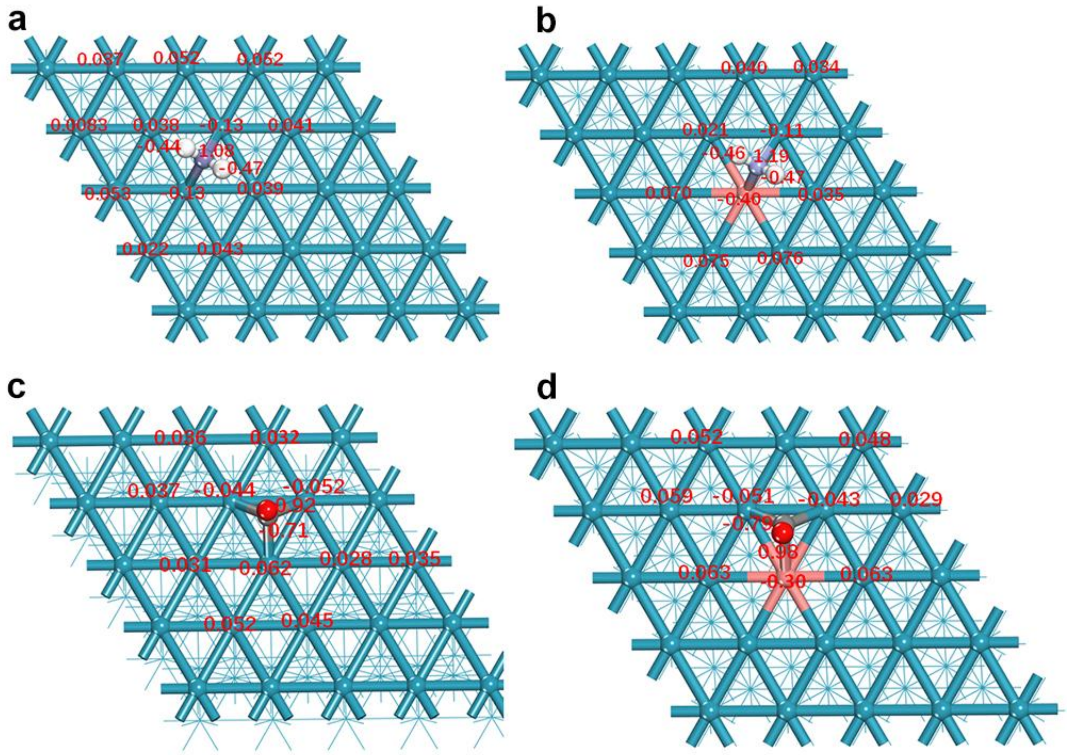

Supplementary Fig. 35. Bader charge analysis of the two main intermediate species (*CO and *NH_2) on Pd(111) and $Cu_1Pd(111)$ surfaces: (a) Pd(111)-*NH_2, (b) $Cu_1Pd(111)$-*NH_2, (c) Pd(111)-*CO, (d) $Cu_1Pd(111)$-*CO.

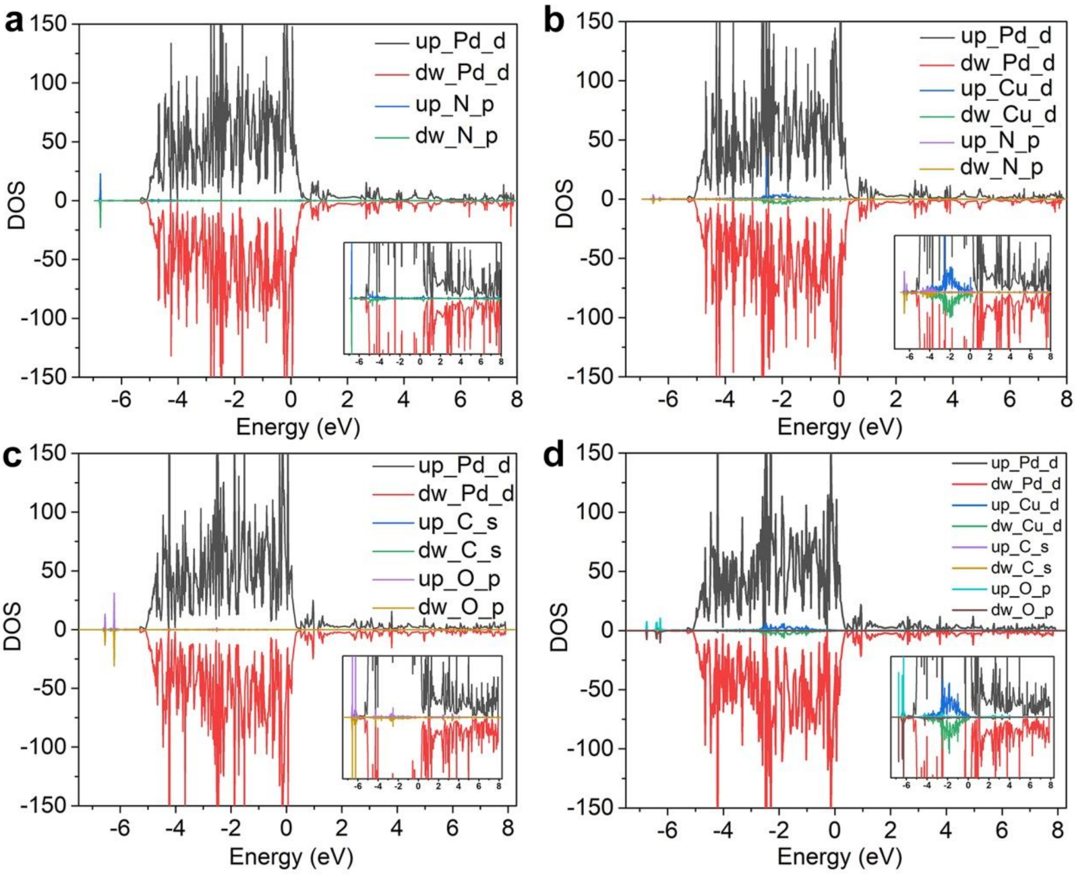

Supplementary Fig. 36. Projected electronic densities of states of the Pd d and Cu d orbitals on Pd(111) and $Cu_1Pd(111)$, and those of the Pd d, Cu d, C s, N p and O p orbitals on $Cu_1Pd(111)$ surfaces.

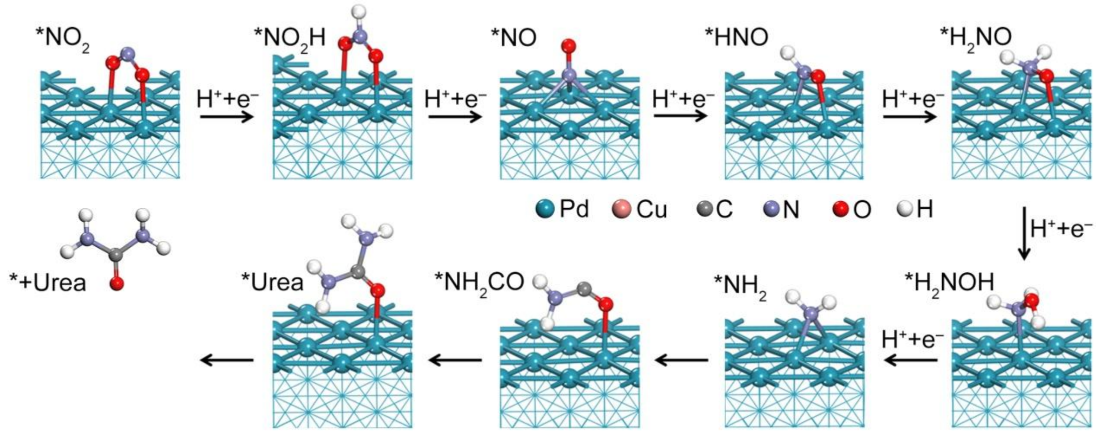

Supplementary Fig. 37. DFT-calculated urea formation cycle on Pd(111) surface.

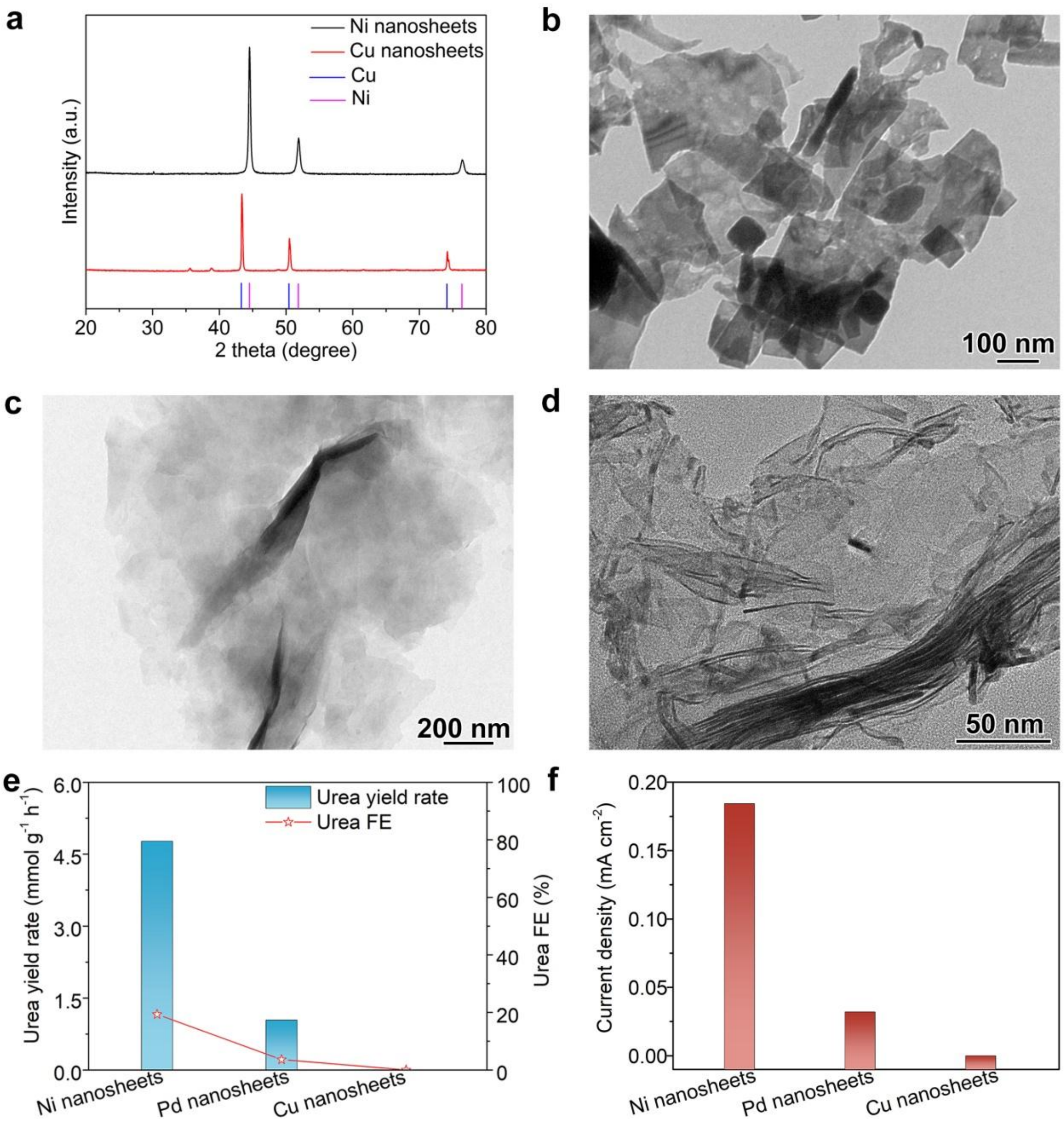

Supplementary Fig. 38. (a) XRD patterns of Cu nanosheets and Ni nanosheets. TEM images of (b) Cu nanosheets, (c) Ni nanosheets and (d) Pd nanosheets. (e) Urea yield rates and urea FEs, (f) urea partial current density.

Notes: Ni nanosheets, Pd nanosheets and Cu nanosheets were synthesized to make a comprehensive comparison. As shown in Supplementary Fig. 38, urea yield rates and urea FEs at –0.5 V are 4.8, 1.0, 0 mmol g^{-1} h^{-1}, 19.3%, 3.6% and 0% for Ni nanosheets, Pd nanosheets and Cu nanosheets, respectively.

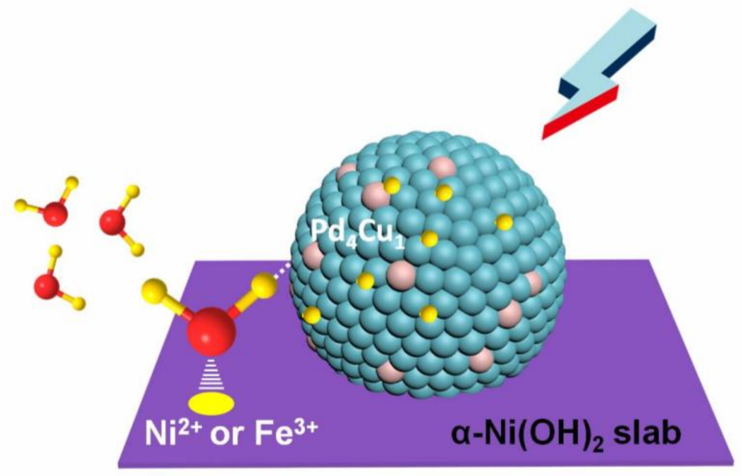

Supplementary Fig. 39. Schematic demonstration the promotion effect of $Pd_4Cu_1/Ni(OH)_2$ interface on water dissociation in alkaline media.

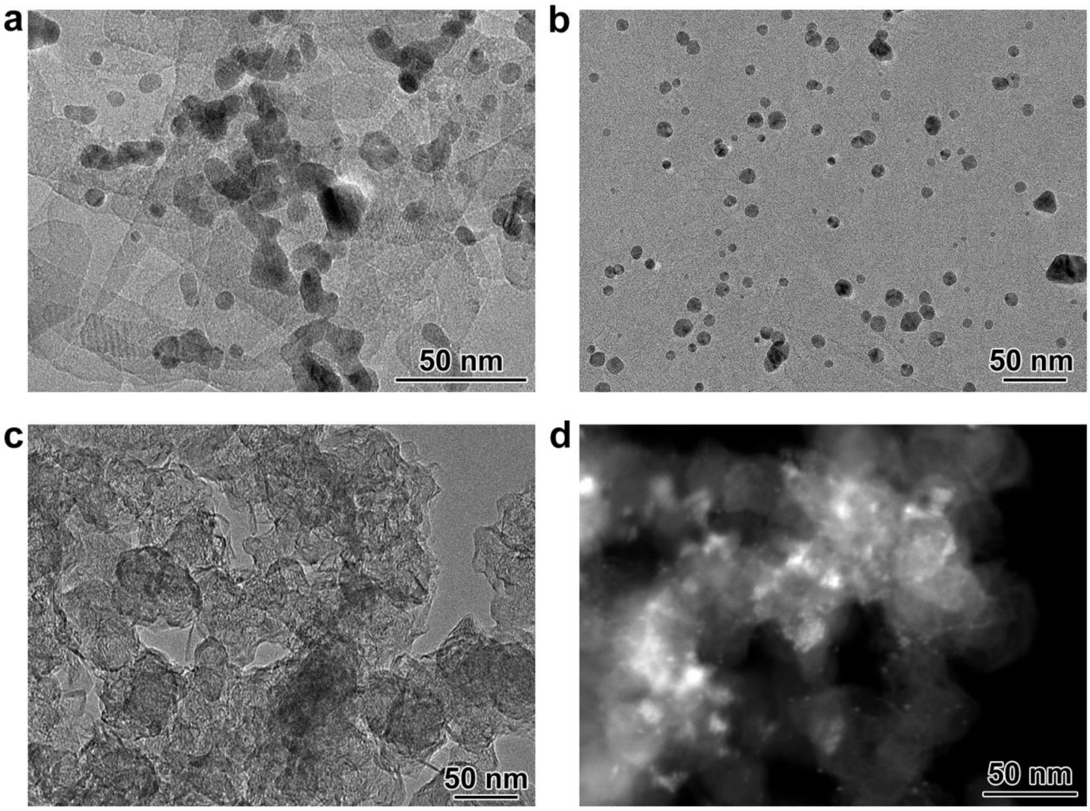

Supplementary Fig. 40. TEM images of (a) Pd_4Cu_1-TiO_2, (b) Pd_4Cu_1-rGO, (c) Pd_4Cu_1-XC-72. (d) HAADF-STEM image of Pd_4Cu_1-XC-72.

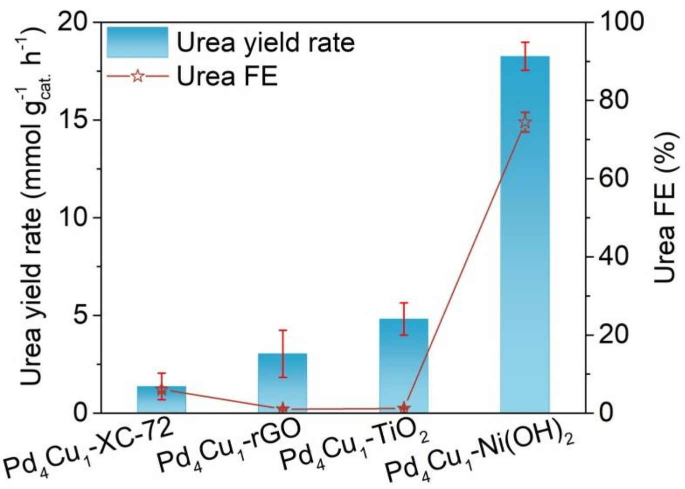

Supplementary Fig. 41. Urea yield rates and FEs for Pd_4Cu_1 clusters with different carriers. Error bars in accordance with the standard deviation of at least three independent measurements.

Notes: $Ni(OH)_2$ nanosheets were replaced by XC-72, rGO and TiO_2 nanosheets to investigate the role of the carrier (Supplementary Fig. 40). As shown in Supplementary Fig. 41, urea yield rates and FEs are 1.4 and 3.0 mmol $g_{cat.}^{-1}$ h^{-1}, 6.1% and 1.0% using XC-72 and rGO as carriers with good electrical conductivity. The results indicate that the conductivity of the carrier is not responsible for the high performance of urea electrosynthesis. Then, semiconductor (TiO_2 nanosheets) were employed as carrier, urea yield rate and FE are still lower than that of $Ni(OH)_2$, suggesting the unique role of $Pd_4Cu_1/Ni(OH)_2$ interface on urea electrosynthesis.

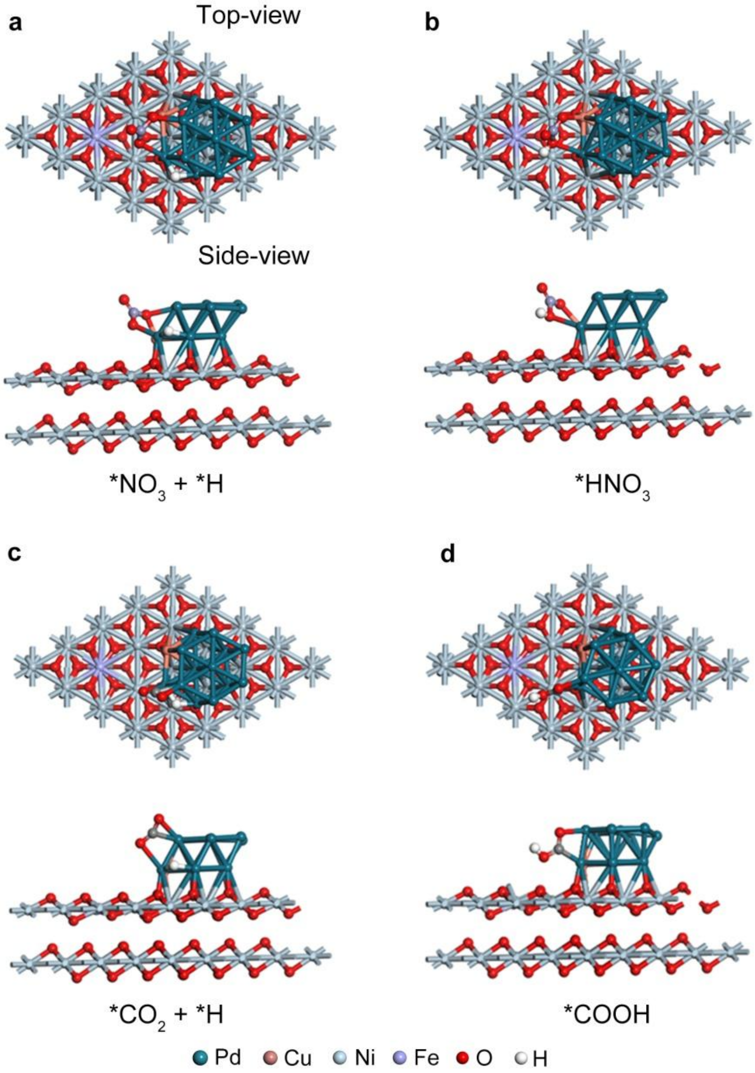

Supplementary Fig. 42. Adsorption configurations of (a) $*NO_3$ + *H, (b) $*HNO_3$, (c) $*CO_2$ + *H and (d) *COOH on $Cu_1Pd\text{-}FeNi(OH)_2$.

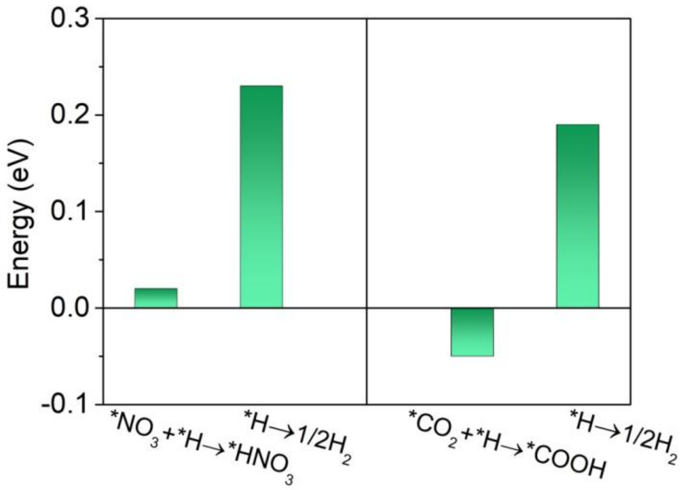

Supplementary Fig. 43. The energy barriers of H–H coupling to form H_2 or the hydrogenation of $*NO_3$ and $*CO_2$ to form $*HNO_3$ and *COOH.

Notes: In view of the enhanced the dissociation of H–OH bond, we then calculated the energy barriers of H–H coupling to form H_2 and the hydrogenation of $*NO_3$ and $*CO_2$ processes in the co-existence of *H, $*NO_3$ and $*CO_2$. As shown in Supplementary Fig. 42, 43, the energy barriers are 0.23 eV for H–H coupling to release H_2 and 0.02 eV for $*NO_3+*H \rightarrow *HNO_3$ process in the co-existence of *H and $*NO_3$, suggesting that the produced active H atoms tend to add to adjacent $*NO_3$, instead of H_2 evolution, which guarantees high urea FE. Similarly, active *H atoms tend to add to adjacent $*CO_2$ to trigger CO_2RR in the co-existence of *H and $*CO_2$.

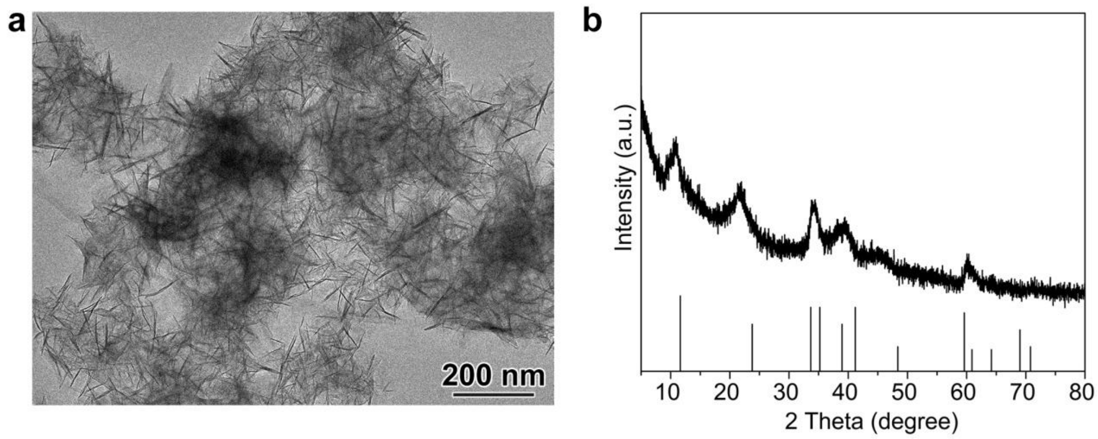

Supplementary Fig. 44. (a) TEM image of Fe-doped $Ni(OH)_2$ nanosheets and (b) XRD pattern of Pd_4Cu_1-$FeNi(OH)_2$.

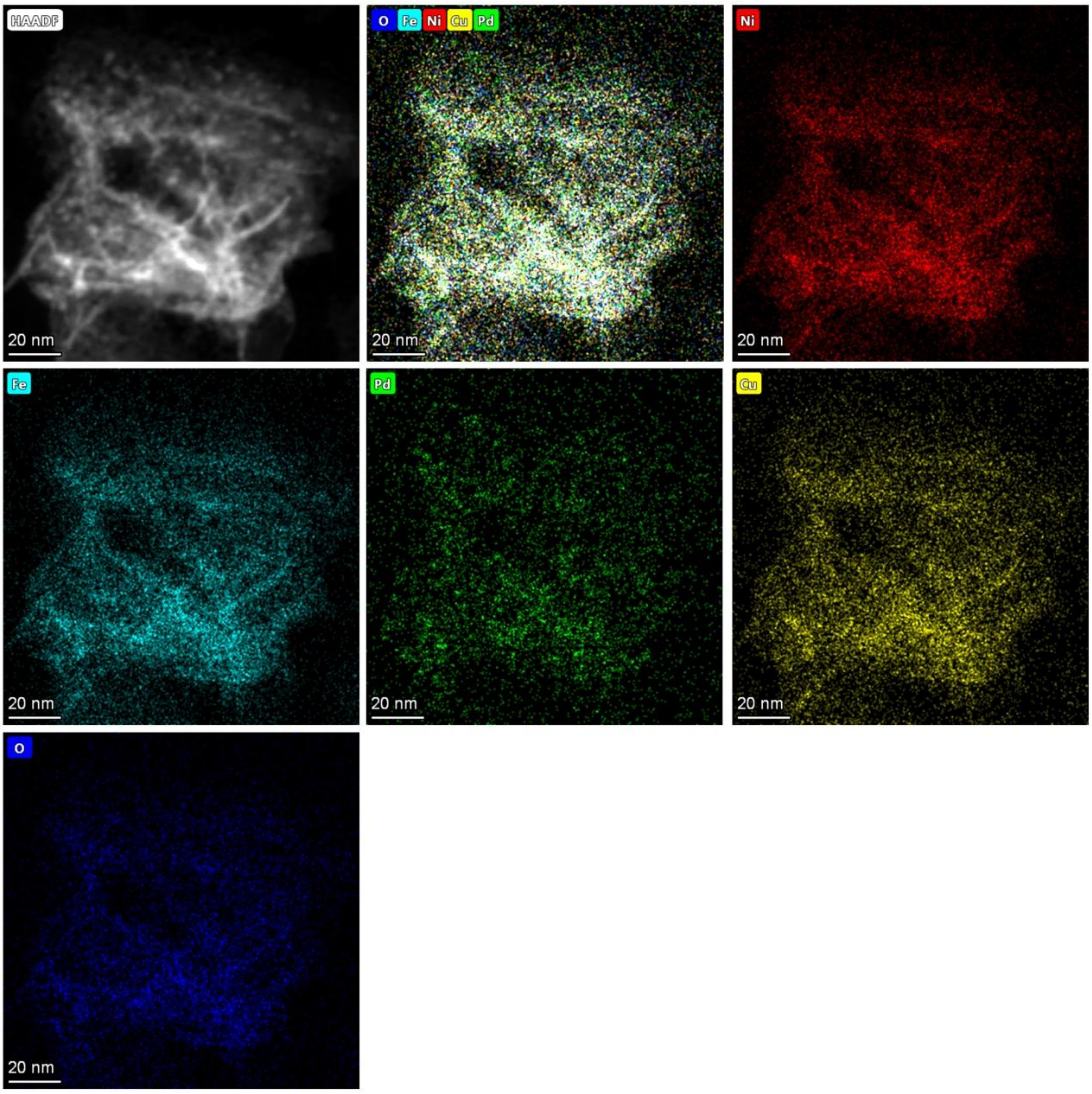

Supplementary Fig. 45. Elemental mapping profiles of Pd_4Cu_1-FeNi$(OH)_2$ composite sample, Ni (red), Fe (cyan), Pd (green), Cu (yellow) and O (blue).

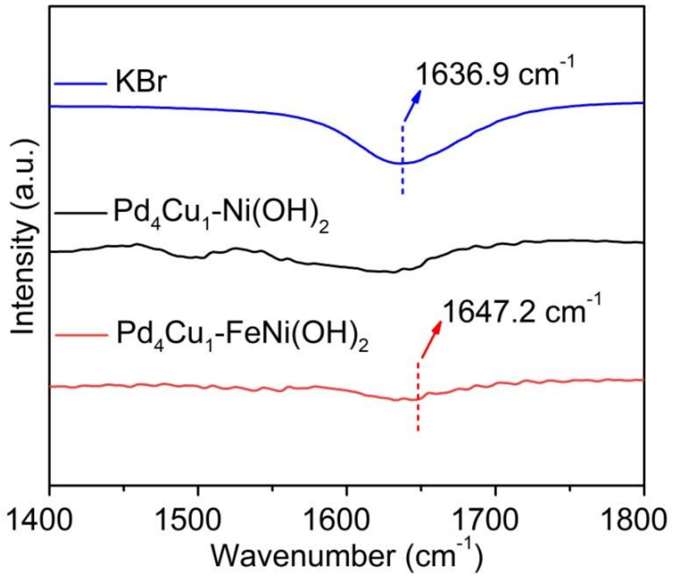

Supplementary Fig. 46. Infrared spectra of KBr, Pd_4Cu_1-$Ni(OH)_2$ and Pd_4Cu_1-$FeNi(OH)_2$ after trace water adsorption.

Notes: As H_2O molecule tends to adsorb on Pd_4Cu_1/$Ni(OH)_2$ interface by forming $Ni^{\delta+}\cdots O^{2-}H\cdots Pd_4Cu_1$ interaction, the bending mode of H–OH shifts to higher energy region. As shown in Supplementary Fig. 46, infrared spectra of adsorbed H_2O on KBr, Pd_4Cu_1-$Ni(OH)_2$ and Pd_4Cu_1-$FeNi(OH)_2$ composite samples confirm this conclusion.

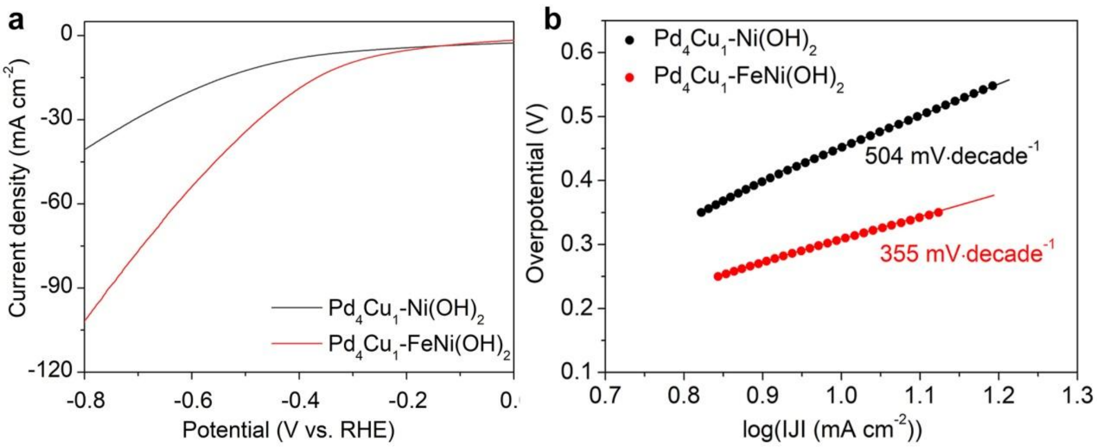

Supplementary Fig. 47. (a) LSV curves and (b) the corresponding Tafel plots of Pd_4Cu_1-$Ni(OH)_2$ and Pd_4Cu_1-$FeNi(OH)_2$ composite samples.

Notes: Supplementary Fig. 47 shows hydrogen evolution reaction (HER) of Pd_4Cu_1-$Ni(OH)_2$ and Pd_4Cu_1-$FeNi(OH)_2$ composite catalysts carried out in 0.1 M KOH. LSV curves and Tafel plots indicate that HER is really promoted by Fe^{3+} doping in $Ni(OH)_2$ carrier.

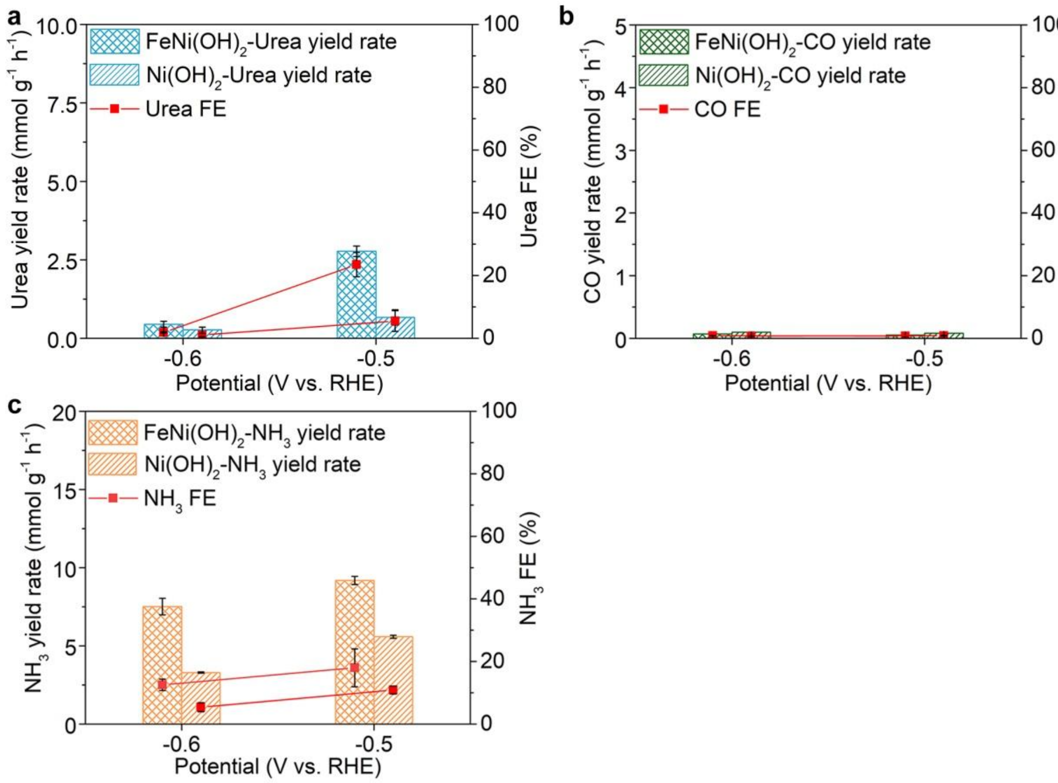

Supplementary Fig. 48. The comparison of $FeNi(OH)_2$ and $Ni(OH)_2$ nanosheets in (a) urea production, (b) solo CO_2RR and (c) NO_3RR. (a-c) Error bars in accordance with the standard deviation of at least three independent measurements.

Notes: In order to reveal the role of Fe doping in $Ni(OH)_2$ nanosheets on the final urea production, some control experiments were performed. As shown in Supplementary Fig. 48, urea yield rates and urea FE for $FeNi(OH)_2$ nanosheets are 2.7 mmol $g_{cat.}^{-1}$ h^{-1} and 23.4% at –0.5 V, respectively. The urea yield rate and FE are comparable with that of $Ni(OH)_2$ nanosheets (0.7 mmol g^{-1} h^{-1}, 5.5%), much lower than that of Pd_4Cu_1-$Ni(OH)_2$ (18.8 mmol $g_{cat.}^{-1}$ h^{-1}, 76.2%). The results indicate that Fe-doped $Ni(OH)_2$ are not the active site for electrochemical C–N coupling toward urea formation. Then, the solo NO_3RR and CO_2RR were also carried out (Supplementary Fig. 48b, 48c). $FeNi(OH)_2$ and $Ni(OH)_2$ nanosheets are inert for CO_2RR to CO. NH_3 yield rate and NH_3 FE are all increased after Fe doping in $Ni(OH)_2$, but still much lower than that of Pd_4Cu_1-$Ni(OH)_2$, suggesting that Pd_4Cu_1 clusters are the real active sites for CO_2RR, NO_3RR and C–N coupling. The results also indicate that Fe doping in $Ni(OH)_2$ has minimal impact on solo CO_2RR, NO_3RR, but promotes water dissociation to produce more active H atoms on Pd_4Cu_1 surface. As such, more $*NH_2$ and *CO are formed on surface and then urea yield rate is greatly improved.

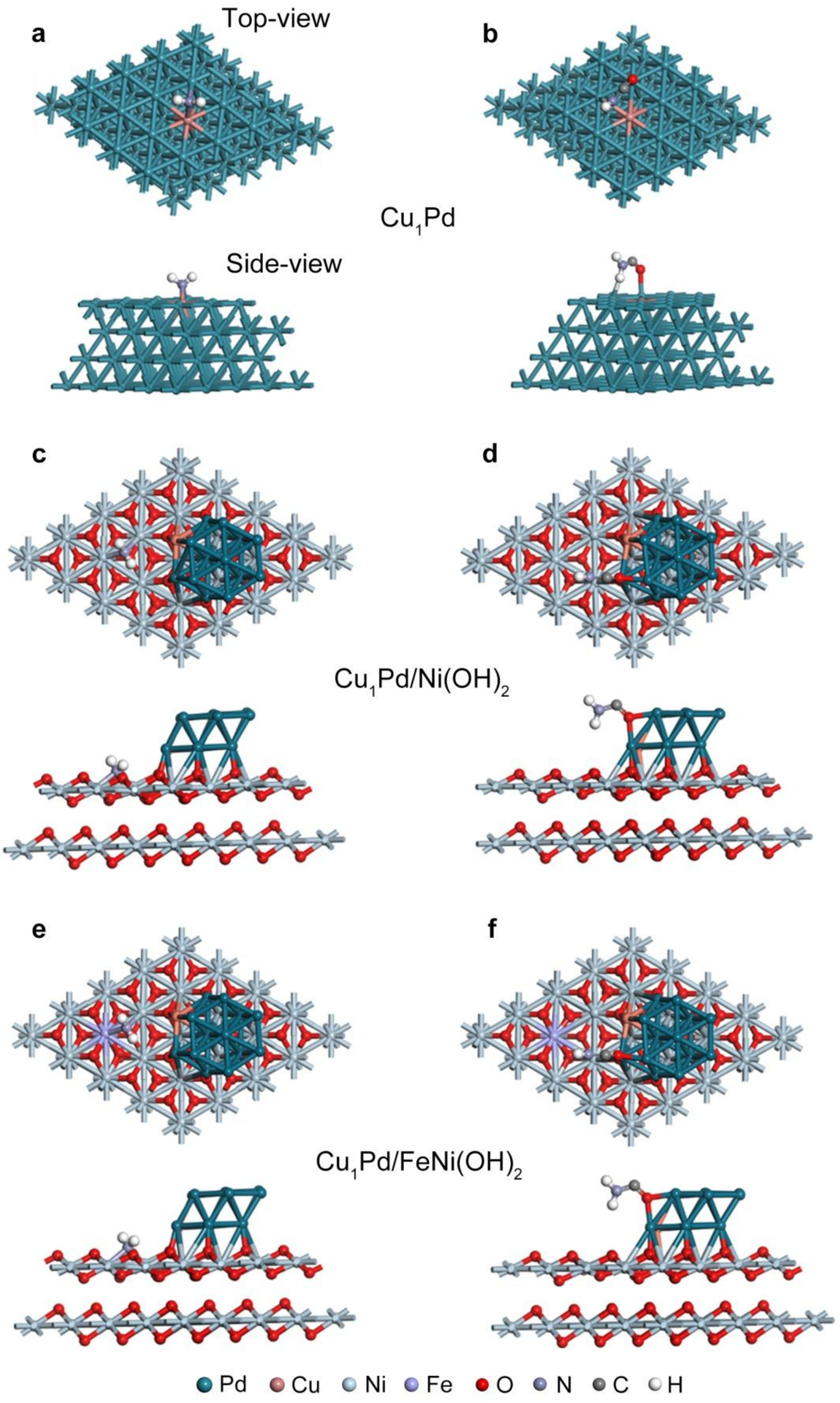

Supplementary Fig. 49. Adsorption configurations of the first C–N coupling of $*NH_2$ and $*CO$ to form $*CONH_2$ on (a,b) Cu_1Pd surface, (c,d) $Cu_1Pd/Ni(OH)_2$ and (e,f) $Cu_1Pd/FeNi(OH)_2$ interface.

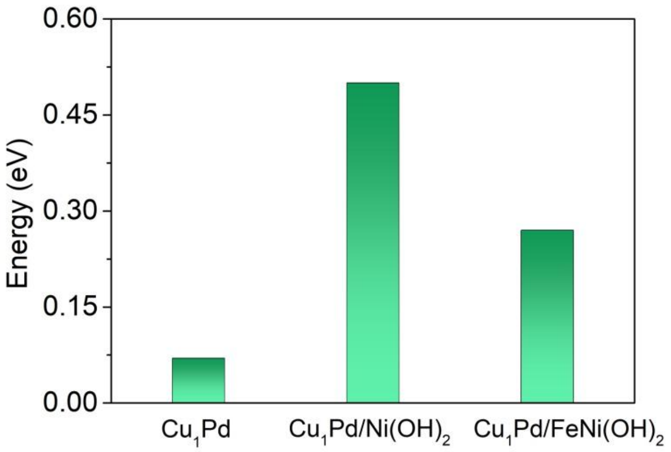

Supplementary Fig. 50. The energy barriers of the first C–N coupling of *NH_2 and *CO to form *$CONH_2$ on Cu_1Pd surface, $Cu_1Pd/Ni(OH)_2$ and $Cu_1Pd/FeNi(OH)_2$ interface.

Notes: To clarify whether the possibility of C–N coupling occurred on $Pd_4Cu_1/Ni(OH)_2$ interface as $Ni(OH)_2$ and Fe-doped $Ni(OH)_2$ have certain catalyze NO_3RR ability. As such, the produced *NH_2 on $Ni(OH)_2$ or $FeNi(OH)_2$ has a possibility to couple with adjacent *CO adsorbed on Pd_4Cu_1 surface on the interface. As shown in Supplementary Fig. 49, 50. The energy barriers for the first C–N coupling of *NH_2 and *CO to form *$CONH_2$ on the $Cu_1Pd/Ni(OH)_2$ and $Cu_1Pd/FeNi(OH)_2$ interface are 0.50 and 0.27 eV, respectively, higher than that on Cu_1Pd surface (0.07 eV). The result indicates that C–N coupling tends to occur on Pd_4Cu_1 surface.

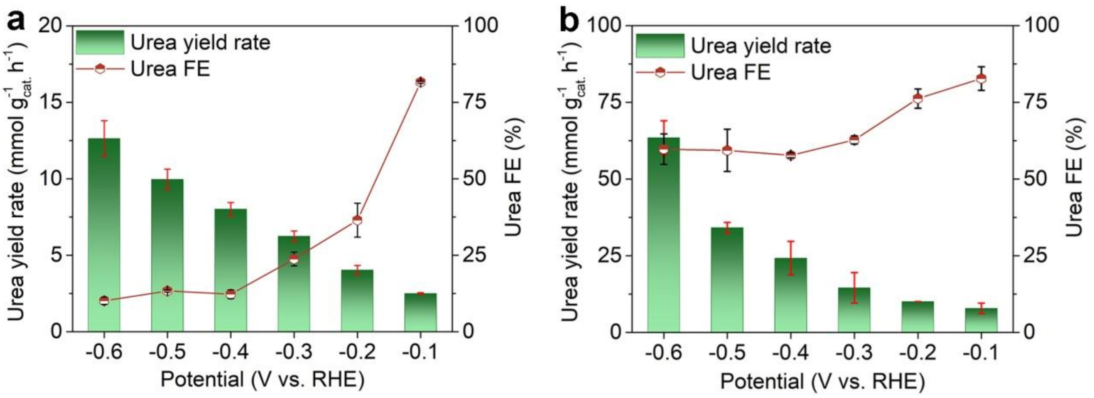

Supplementary Fig. 51. Urea yield rates and FEs using Pd_4Cu_1-$FeNi(OH)_2$ composite catalyst with different amounts of $NaBH_4$ assessed in H-cell, (a) 6 μmol, (b) 18 μmol. (a, b) Error bars in accordance with the standard deviation of at least three independent measurements.

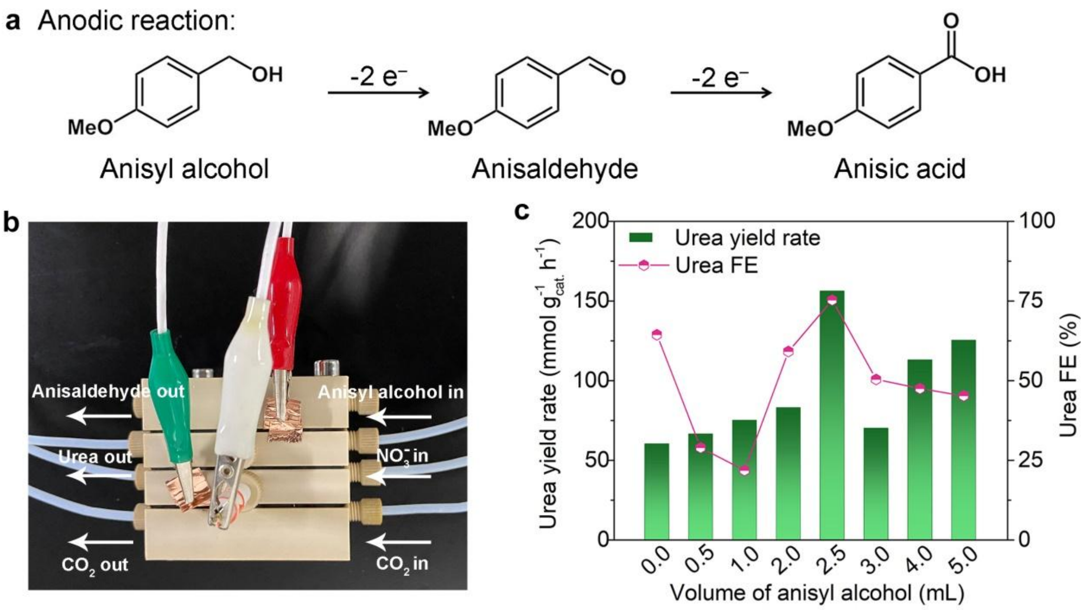

Supplementary Fig. 52. (a) Oxidation of anisyl alcohol at anode, (b) photograph of an actual GDE for urea electrosynthesis coupled with anisyl alcohol oxidation at anode. (c) The relationship of urea yield rates and FEs with different volumes of anisyl alcohol at anode using Pd_4Cu_1-$FeNi(OH)_2$ as a catalyst.

Notes: The oxidation of anisyl alcohol coupled with urea electrosynthesis at anode not only can acquire high-value anisaldehyde, but also lower cell voltage of the whole electrolysis. The total FE for anode and cathode can be greatly enhanced. As shown in Supplementary Fig. 52c, urea yield rate and FE are all enhanced with coupling oxidation of anisyl alcohol (2.5 mL) at anode using Pd_4Cu_1-$FeNi(OH)_2$ as a catalyst.

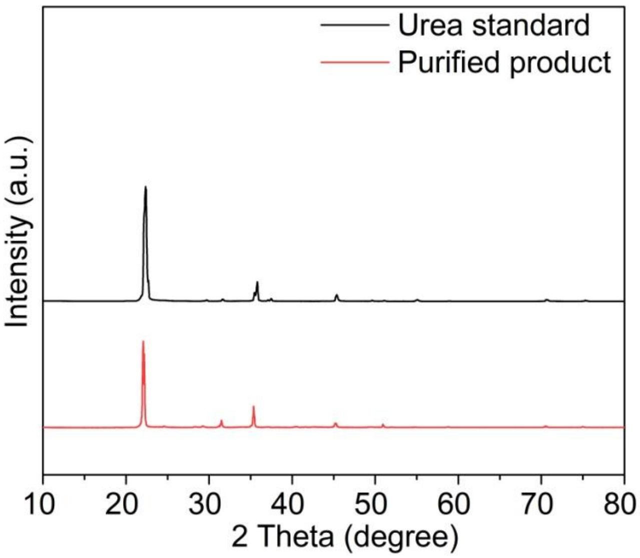

Supplementary Fig. 53. XRD pattern of purified urea product from the electrolyte.

Supplementary Table 1. The molar ratios of Pd to Cu in Pd_xCu_1-$Ni(OH)_2$ composite samples determined by ICP-MS.

Catalyst	Molar ratio of Pd:Cu
Pd_1Cu_1-$Ni(OH)_2$	1.1:1
Pd_2Cu_1-$Ni(OH)_2$	2.0:1
Pd_3Cu_1-$Ni(OH)_2$	3.0:1
Pd_4Cu_1-$Ni(OH)_2$	3.8:1
Pd_5Cu_1-$Ni(OH)_2$	5.1:1
Pd_6Cu_1-$Ni(OH)_2$	5.9:1

Supplementary Table 2. EXAFS fitting parameters at the Pd, Cu and Ni K-edge for various samples (S_0^2=1.0).

Sample	Path	N^a	R(Å)^b	σ^2(Å^2)^c	ΔE_0(eV)^d	R factor
Cu foil	Cu-Cu	12.00	2.54	0.0098	4.21	0.0076
CuO	Cu-O	6.00	1.95	0.0061	7.45	0.0163
	Cu-Cu	7.92	2.89	0.0153	2.22	
Pd foil	Pd-Pd	11.66	2.74	0.0051	-6.20	0.0033
Sample Cu	Cu-O	3.05	2.05	0.0157	9.07	0.0191
	Cu-Pd	10.71	2.61	0.0120	-5.09	
Sample Pd	Pd-Cu	1.35	2.62	0.0040	-8.64	0.0067
	Pd-Pd	7.87	2.70	0.0102	-6.15	
Ni foil	Ni-Ni	12	2.48	0.0062	5.9	0.0016

^{a}CN, coordination number; ^bR, distance between absorber and backscatter atoms; $^c\sigma^2$, Debye-Waller factor to account for both thermal and structural disorders; $^d\Delta E_0$, inner potential correction; R factor indicates the goodness of the fit. Fitting range: 3.0 ≤ k (/Å) ≤ 12.0 and 1.2 ≤ R (Å) ≤ 3.0 (Cu foil).; 3.0 ≤ k (/Å) ≤ 11.2 and 1.0 ≤ R (Å) ≤ 2.8 (CuO).; 3.0 ≤ k (/Å) ≤ 12.0 and 1.0 ≤ R (Å) ≤ 3.0 (Pd foil).;3.0 ≤ k (/Å) ≤ 15.7 and 1.0 ≤ R (Å) ≤ 3.0 (Sample Cu).; 3.0 ≤ k (/Å) ≤ 14.1 and 1.0 ≤ R (Å) ≤ 3.0(Sample Pd).

Supplementary Table 3. Urea electrosynthesis from C–N coupling.

Catalyst	N-source	Electrolyte	FE (%)	Potential	Yield rate	J_{urea}	Stability (h)	Electrode configuration	Ref.
Te-doped Pd	NO_2^-	0.1 M $KHCO_3$ + 0.01 M KNO_2	12.2	–1.1	/	0.10	5	H-Cell	S4
ZnO-V_o	NO_2^-	0.2 M $NaHCO_3$ + 0.1 M $NaNO_2$	23.3	–0.79	/	6.06	15	H-Cell	S5
Cu-TiO_2-V_o	NO_2^-	0.2 M $KHCO_3$ + 0.02 M KNO_2	43.1	–0.4	20.8	2.59	2	H-Cell	S6
Cd	NO_2^-	0.2 M $KHCO_3$ + 0.02 KNO_3	55	–0.6		/		GDE	S7
AuCu	NO_2^-	0.5 M $KHCO_3$ + 0.01 M KNO_2	24.7	–1.1	64.8	0.49		H-Cell	S8
Co-NiO_x @GDY	NO_2^-	0.01 M $NaNO_2$	64.3	–0.7	15.2	3.86		H-cell	S9
TiO_2-V_o/PdCu	N_2	0.1 M $KHCO_3$	8.9	–0.4	3.4	0.05	12	GDE	S10
Bi/$BiVO_4$	N_2	0.1 M $KHCO_3$	12.6	–0.4	5.9	0.48	10	H-Cell	S11
$BiFeO_3$/ $BiVO_4$	N_2	0.1 M $KHCO_3$	17	–0.4	4.9	3.06	10	H-Cell	S12
$Ni_3(BO_3)_2$	N_2	0.1 M $KHCO_3$	20.4	–0.5	9.7	0.33	20	H-Cell	S13
InOOH	N_2	0.1 M $KHCO_3$	21.0	–0.4	6.9	0.13	5	H-Cell	S14
Co-PMDA-2-mbIM	N_2		48.97	–0.5	14.5	0.98		H-cell	S15
Zn NBs	NO	0.2 M $KHCO_3$	11.3	–0.92	15.1	4.52	15	H-Cell	S16
Fe-Ni	NO_3^-	0.1 M $KHCO_3$+ 50 mM KNO_3	17.8	–1.5	20.2	8.01		H-Cell	S17
XC72R-AuPd	NO_3^-	0.075 M $KHCO_3$ +0.025 M KNO_3	15.6	–0.6	3.4	1.40		H-cell	S18
V_o-CeO_2-750	NO_3^-	/	/	–1.6	15.7	/	5	H-cell	S19
Fe(a)@C-Fe_3O_4/CNTs)	NO_3^-	0.1 M KNO_3+0.1 M KSO_4	22.4	–0.65	16.5	0.72		H-cell	S20
$Cu_{97}In_3$-C	NO_3^-	0.1 M $KHCO_3$ + 0.01 M KNO_3			13.1	/		H-cell	S21
Cu_1-CeO_2	NO_3^-	0.1 M $KHCO_3$ + 0.05 M KNO_3			52.8	/		H-cell	S22
Zn	NO_3^-	0.2 M $KHCO_3$ + 0.02 M KNO_3	35	–1.75	/	7.11		GDE	S23
Ni-Pc	NO_3^-	0.2 M $KHCO_3$ + 0.02 KNO_3	40	–1.5	/	4.40		GDE	S24
TiO_2/Nafion	NO_3^-	0.1 M KNO_3	40	–0.5	/	0.32	2.15	H-Cell	S25

$In(OH)_3$	NO_3^-	0.1 M KNO_3	53.4	–0.6	8.9	0.53	12	H-Cell	S3
V_o-InOOH	NO_3^-	0.1 M KNO_3	51.0	–0.4	9.9	0.26	24	H-Cell	S26
**Pd_4Cu_1-Ni(OH)$_2$**	**NO_3^-**	**0.1 M $KHCO_3$ + 0.1 M KNO_3**	**64.4**	**–0.5**	**60.4**	**2.30**	**380**	**GDE**	**This work**
**Pd_4Cu_1-FeNi(OH)$_2$**	**NO_3^-**	**0.1 M $KHCO_3$ + 0.1 M KNO_3**	**66.4**	**–0.6**	**436.9**	**7.05**	**1000**	**GDE**	**This work**

Note: The units for urea yield rate and urea partial current density are mmol $g_{cat.}^{-1}$ h^{-1} mA cm^{-2}, respectively.

Supplementary Table 4. Observed Raman frequencies and assignments for formamide and urea in Supplementary Fig. 31.

Formamide		Urea	
Freq, cm^{-1}	assignment	Freq, cm^{-1}	assignment
602	OCN bending	1000	ν(C-N)
1091	NH_2 rock		
1308	ν(C-N)		
1390	C-H in-plane bending		
1598	δNH_2		
1668	H bonded formamide		

Supplementary Table 5. The Gibbs free energy change (Δ*G*) of reaction for CO_2RR to CO on $Cu_1Pd(111)$ and Pd(111) at 0 V (vs. RHE).

Intermediates	$Cu_1Pd(111)$	Pd(111)
*+CO_2	0	0
*CO_2	0.32	0.34
*COOH	0.27	0.24
*CO	-0.89	-0.86
*+CO	0.70	0.70

Supplementary Table 6. The Gibbs free energy change (ΔG) of reaction for urea formation on $Cu_1Pd(111)$, Pd(111), Cu(111) and Ni(111) at 0 V (vs. RHE).

Intermediates	$Cu_1Pd(111)$	Pd(111)	Cu(111)	Ni(111)
$*NO_2$	0	0	0	0
$*NO_2H$	0.39	0.39	0.57	0.50
*NO	-1.87	-2.17	-0.91	-1.10
*HNO	-1.13	-1.02	-0.46	-0.01
$*H_2NO$	-1.37	-1.60	-1.46	-1.56
$*H_2NOH$	-1.56	-1.96	-1.34	-1.68
$*NH_2$	-3.44	-3.55	-3.55	-3.04
$*NH_2CO$	-3.37	-3.36	-2.93	-2.72
$*NH_2CONH_2$	-9.81	-9.82	-9.57	-9.50
$*+NH_2CONH_2$	-10.36	-10.33	-10.27	-10.03

Reference:

S1. M. Li, Y. Chen, W. Li, X. Li, H. Tian, X. Wei, Z. Ren, G. Han, Ultrathin anatase TiO_2 nanosheets for high-performance photocatalytic hydrogen production, *Small* **2017**, *13*, 1604115.

S2. A. N. Mansour, C. A. Melendres, Characterization of α-$Ni(OH)_2$ by XPS, *Surf. Sci. Spectra* **1994**, *3*, 255−262.

S3. C. Lv, L. Zhong, H. Liu, Z. Fang, C. Yan, M. Chen, Y. Kong, C. Lee, D. Liu, S. Li, J. Liu, L. Song, G. Chen, Q. Yan, G. Yu, Selective electrocatalytic synthesis of urea with nitrate and carbon dioxide, *Nat. Sustain.* **2021**, *4*, 868–876.

S4. Y. Feng, H. Yang, Y. Zhang, X. Huang, L. Li, T. Cheng, Q. Shao, Te-doped Pd nanocrystal for electrochemical urea production by efficiently coupling carbon dioxide reduction with nitrite reduction, *Nano Lett.* **2020**, *20*, 8282–8289.

S5. N. Meng, Y. Huang, Y. Liu, Y. Yu, B. Zhang, Electrosynthesis of urea from nitrite and CO_2 over oxygen vacancy-rich ZnO porous nanosheets, *Cell Rep. Phys. Sci.* **2021**, *2*, 100378.

S6. N. Cao, Y. Quan, A. Guan, C. Yang, Y. Ji, L. Zhang, G. Zheng, Oxygen vacancies enhanced cooperative electrocatalytic reduction of carbon dioxide and nitrite ions to urea, *J. Colloid Interface Sci.* **2020**, *577*, 109–114.

S7. M. Shibata, K. Yoshida, N. Furuya, Electrochemical synthesis of urea at gas-diffusion electrodes: III. Simultaneous reduction of carbon dioxide and nitrite ions with various metal catalysts, *J. Electrochem. Soc.* **1998**, *145*, 595–600.

S8. S. Liu, S. Yin, Z. Wang, Y. Xu, X. Li, L. Wang, H. Wang, AuCu nanofibers for electrosynthesis of urea from carbon dioxide and nitrite, *Cell Rep. Phys. Sci.* **2022**, *3*, 100869.

S9. D. Zhang, Y. Xue, X. Zheng, C. Zhang, Y. Li, Multi-heterointerfaces for selective and efficient urea production, *Nat. Sci. Rev.*, **2022**, *nwac209*.

S10. C. Chen, X. Zhu, X. Wen, Y. Zhou, L. Zhou, H. Li, L. Tao, Q. Li, S. Du, T. Liu, D. Yan, C. Xie, Y. Zou, Y. Wang, R. Chen, J. Huo, Y. Li, J. Cheng, H. Su, X. Zhao, W. Cheng, Q. Liu, H. Lin, J. Luo, J. Chen, M. Dong, K. Cheng, C. Li, S. Wang, Coupling N_2 and CO_2 in H_2O to synthesize urea under ambient conditions, *Nat. Chem.* **2020**, *12*, 717–724.

S11. M. Yuan, J. Chen, Y. Bai, Z. Liu, J. Zhang, T. Zhao, Q. Wang, S. Li, H. He, G. Zhang, Unveiling electrochemical urea synthesis by co-activation of CO_2 and N_2 with Mott–Schottky heterostructure catalysts, *Angew. Chem. Int. Ed.* **2021**, *60*, 10910–10918.

S12. M. Yuan, J. Chen, Y. Bai, Z. Liu, J. Zhang, T. Zhao, Q. Shi, S. Li, X. Wang, G. Zhang, Electrochemical C–N coupling with perovskite hybrids toward efficient urea synthesis, *Chem. Sci.* **2021**, *12*, 6048–6058.

S13. M. Yuan, J. Chen, Y. Xu, R. Liu, T. Zhao, J. Zhang, Z. Ren, Z. Liu, C. Streb, H. He, C. Yang, S. Zhang, G. Zhang, Highly selective electroreduction of N_2 and CO_2 to urea over artificial frustrated Lewis pairs, *Energy Environ. Sci.* **2021**, *14*, 6605–6615.

S14. M. Yuan, H. Zhang, Y. Xu, R. Liu, R. Wang, T. Zhao, J. Zhang, Z. Liu, H. He, C. Yang, S. Zhang, G. Zhang, Artificial frustrated Lewis pairs facilitating the electrochemical N_2 and CO_2 conversion to urea, *Chem. Catal.* **2022**, *2*, 309–320.

S15. M. Yuan, J. Chen, H. Zhang, Q. Li, L. Zhou, C. Yang, R. Liu, Z. Liu, S. Zhang and G. Zhang, Host-guest molecular interaction promoted urea electrosynthesis over precisely designed conductive metal-organic Frameworks. *Energy Environ. Sci*. **2022**, *15*, 2084–2095.
S16. Y. Huang, R. Yang, C. Wang, N. Meng, Y. Shi, Y. Yu, B. Zhang, Direct electrosynthesis of urea from carbon dioxide and nitric oxide, *ACS Energy Lett*. **2022**, *7*, 284−291.
S17. X. Zhang, X. Zhu, S. Bo, C. Chen, M. Qiu, X. Wei, N. He, C. Xie, W. Chen, J. Zheng, P. Chen, S. Jiang, Y. Li, Q. N. Liu, S. Wang, Identifying and tailoring C–N coupling site for efficient urea synthesis over diatomic Fe–Ni catalyst, *Nat. Commun*. **2022**, *13*, 5337.
S18. H.Wang,Y. Jiang, S. Li, F. Gou, X. Liu, Y. Jiang, W. Luo, W. Shen, R. He, M. Li, Realizing efficient C-N coupling via electrochemical co-reduction of CO_2 and NO_3^- on AuPd nanoalloy to form urea: Key C-N coupling intermediate, *Appl. Catal. B Environ.* **2022**, *318*, 121819.
S19. X. Wei, X. Wen, Y. Liu, C. Chen, C. Xie, D. Wang, M. Qiu, N. He, P. Zhou, W. Chen, J. Cheng, H. Lin, J. Jia, X. Fu, S. Wang, Oxygen vacancy-mediated selective C−N coupling toward electrocatalytic urea synthesis, *J. Am. Chem. Soc.* **2022**, *144*, 11530–11535.
S20. J. Geng, S. Ji, M. Jin, C. Zhang, M. Xu, G. Wang, C. Liang, H. Zhang, Ambient electrosynthesis of urea with nitrate and carbon dioxide over iron-based dual-sites, *Angew. Chem. Int. Ed.* **2023**, *62*, e2022109.
S21. Y. Liu, X. Tu, X. Wei, D. Wang, X. Zhang, W. Chen, C. Chen, S. Wang, C-bound or O-bound surface: which one boosts electrocatalytic urea synthesis? *Angew. Chem. Int. Ed.* **2023**, 10.1002/anie.202300387.
S22. X. Wei, Y. Liu, X. Zhu, S. Bo, L. Xiao, C. Chen, T. Nga, Y. He, M. Qiu, C. Xie, D. Wang, Q. Liu, F. Dong, C. Dong, X. Fu, S. Wang, Dynamic reconstitution between copper single atoms and clusters for electrocatalytic urea synthesis, *Adv. Mater.* **2023**, 2300020.
S23. M. Shibata, K. Yoshida, N. Furuya, Electrochemical synthesis of urea at gas-diffusion electrodes: IV. simultaneous reduction of carbon dioxide and nitrate ions with various metal catalysts, *J. Electrochem. Soc.* **1998**, *145*, 2348–2353.
S24. M. Shibata, N. Furuya, Electrochemical synthesis of urea at gas-diffusion electrodes: Part VI. Simultaneous reduction of carbon dioxide and nitrite ions with various metallophthalocyanine catalysts. *J. Electroanal. Chem.* **2001**, *507*, 177–184.
S25. D. Saravanakumar, J. Song, S. Lee, N. H. Hur, W. Shin, Electrocatalytic conversion of carbon dioxide and nitrate ions to urea by a titania–nafion composite electrode, *ChemSusChem* **2017**, *10*, 3999–4003.
S26. C. Lv, C. Lee, L. Zhong, H. Liu, J. Liu, L. Yang, C. Yan, W. Yu, H. H. Hng, Z. Qi, L. Song, S. Li, K. P. Loh, Q. Yan, G. Yu, A defect engineered electrocatalyst that promotes high-efficiency urea synthesis under ambient conditions, *ACS Nano* **2022**, *16*, 8213–8222.
